# Multiplexed Component Analysis to Identify Genes Contributing to the Immune Response during Acute SIV Infection

**DOI:** 10.1371/journal.pone.0126843

**Published:** 2015-05-18

**Authors:** Iraj Hosseini, Lucio Gama, Feilim Mac Gabhann

**Affiliations:** 1 Institute for Computational Medicine, Department of Biomedical Engineering, Johns Hopkins University, Baltimore, Maryland, United States of America; 2 Department of Molecular and Comparative Pathobiology, The Johns Hopkins School of Medicine, Baltimore, Maryland, United States of America; Harvard Medical School, UNITED STATES

## Abstract

Immune response genes play an important role during acute HIV and SIV infection. Using an SIV macaque model of AIDS and CNS disease, our overall goal was to assess how the expression of genes associated with immune and inflammatory responses are longitudinally changed in different organs or cells during SIV infection. To compare RNA expression of a panel of 88 immune-related genes across time points and among three tissues – spleen, mesenteric lymph nodes (MLN) and peripheral blood mononuclear cells (PBMC) – we designed a set of Nanostring probes. To identify significant genes during acute SIV infection and to investigate whether these genes are tissue-specific or have global roles, we introduce a novel multiplexed component analysis (MCA) method. This combines multivariate analysis methods with multiple preprocessing methods to create a set of 12 “*judges*”; each *judge* emphasizes particular types of change in gene expression to which cells could respond, for example, the absolute or relative size of expression change from baseline. Compared to bivariate analysis methods, our MCA method improved classification rates. This analysis allows us to identify three categories of genes: (a) consensus genes likely to contribute highly to the immune response; (b) genes that would contribute highly to the immune response only if certain assumptions are met – e.g. that the cell responds to relative expression change rather than absolute expression change; and (c) genes whose contribution to immune response appears to be modest. We then compared the results across the three tissues of interest; some genes are consistently highly-contributing in all tissues, while others are specific for certain tissues. Our analysis identified *CCL8*, *CXCL10*, *CXCL11*, *MxA*, *OAS2*, and *OAS1* as top contributing genes, all of which are stimulated by type I interferon. This suggests that the cytokine storm during acute SIV infection is a systemic innate immune response against viral replication. Furthermore, these genes have approximately equal contributions to all tissues, making them possible candidates to be used as non-invasive biomarkers in studying PBMCs instead of MLN and spleen during acute SIV infection experiments. We identified clusters of genes that co-vary together and studied their correlation with regard to other gene clusters. We also developed novel methods to faithfully visualize multi-gene correlations on two-dimensional polar plots, and to visualize tissue specificity of gene expression responses.

## Introduction

Infection by the human immunodeficiency virus (HIV) is characterized by a dramatic and progressive depletion of CD4+ T cells and a sustained state of chronic inflammation and immune activation. Disease progression appears to be directly related to early events during acute infection, including an intense and coordinated production of plasma cytokines (“cytokine storm”) that is not observed in other chronic viral infections, such as Hepatitis type B and C [1]. Studies using macaques infected with simian immunodeficiency virus (SIV) corroborate these findings (S1 Information), and provide insights on the complex network of immune regulatory genes that is triggered in response against the virus [2,3]. Because of the difficulties in establishing the precise time when an individual is infected by HIV, unravelling the effect of genes and their level of significance during acute SIV infection is key in understanding the mechanisms by which these viruses interact with the immune system. Using an SIV macaque model for AIDS and CNS disease, our group has been assessing how the expression of genes associated with immune and inflammatory responses are longitudinally changed in different organs or cells during SIV infection. Because of the large number of tissue samples and to be cost effective, we designed a set of Nanostring probes to measure the expression of 88 immune-related genes that are routinely analyzed in several diseases. These include genes from different families such as chemokines, chemokine receptors, interferons, type I interferon receptors, interleukins, cytokine receptors, interferon regulatory factors, and interferon-stimulated genes (S1 Table). In this paper, we propose to use a novel multivariate analysis method to identify significant genes affecting immune responses in three different lymphoid compartments during acute SIV infection.

Univariate analysis of the gene expressions alone or studying the correlation between gene expressions and output variables such as time since infection and SIV RNA in plasma provides limited success in interpreting the data. This may be due to several reasons. First, the changes in gene expressions are essentially caused by SIV infection. This suggests that the mRNA measurements, regardless of the biological functions of genes, should be correlated with time since infection or SIV RNA in plasma, leading to many “hits” that are not biologically significant. In addition, the data could be noisy and focusing on the co-variance as the only metric can be misleading. Second, it is generally thought that multiple genes work together to orchestrate the immune response during acute SIV infection. Therefore, we use multivariate analysis techniques, which can compensate for the correlations between multiple genes, to study all the genes simultaneously. These techniques, including principal component analysis (PCA), independent component analysis (ICA), and partial least squares (PLS) regression, have been used in various biological applications such as tumor classification [4], biomarker identification in traumatic brain injury [5], predicting age of cytotoxic T cells [6], and classification of yeast gene expression data into biologically meaningful groups [7]. The main differences between univariate and multivariate analysis methods are addressed in a recent review by Saccenti *et al*. [8]. Note that prior quantitative knowledge of how the changes in expression of each gene impact the immune response during acute SIV infection is not available. For example, the system may be more sensitive to changes in the absolute values of mRNA measurement for some genes, but more sensitive to relative changes for other genes. Previous multivariate analysis studies emphasize only one of these possibilities, and therefore selects preferentially for genes that satisfy the assumption—for example, selects for genes with high absolute changes, or only genes with high relative changes. Therefore, preprocessing the data to take into account various initial assumptions is a necessary step for performing a thorough study of the effect of genes on the immune response. Various normalization methods including mean-centering [9,10], autoscaling or unit-variance scaling [10,11], pareto scaling [12,13], maximum scaling [14], range scaling [14,15], vast scaling [16], and maximum likelihood scaling [17,18] have been used prior to multivariate analysis methods. The advantages and disadvantages of these different normalization strategies were discussed in detail in [13,19].

**Fig 1:**
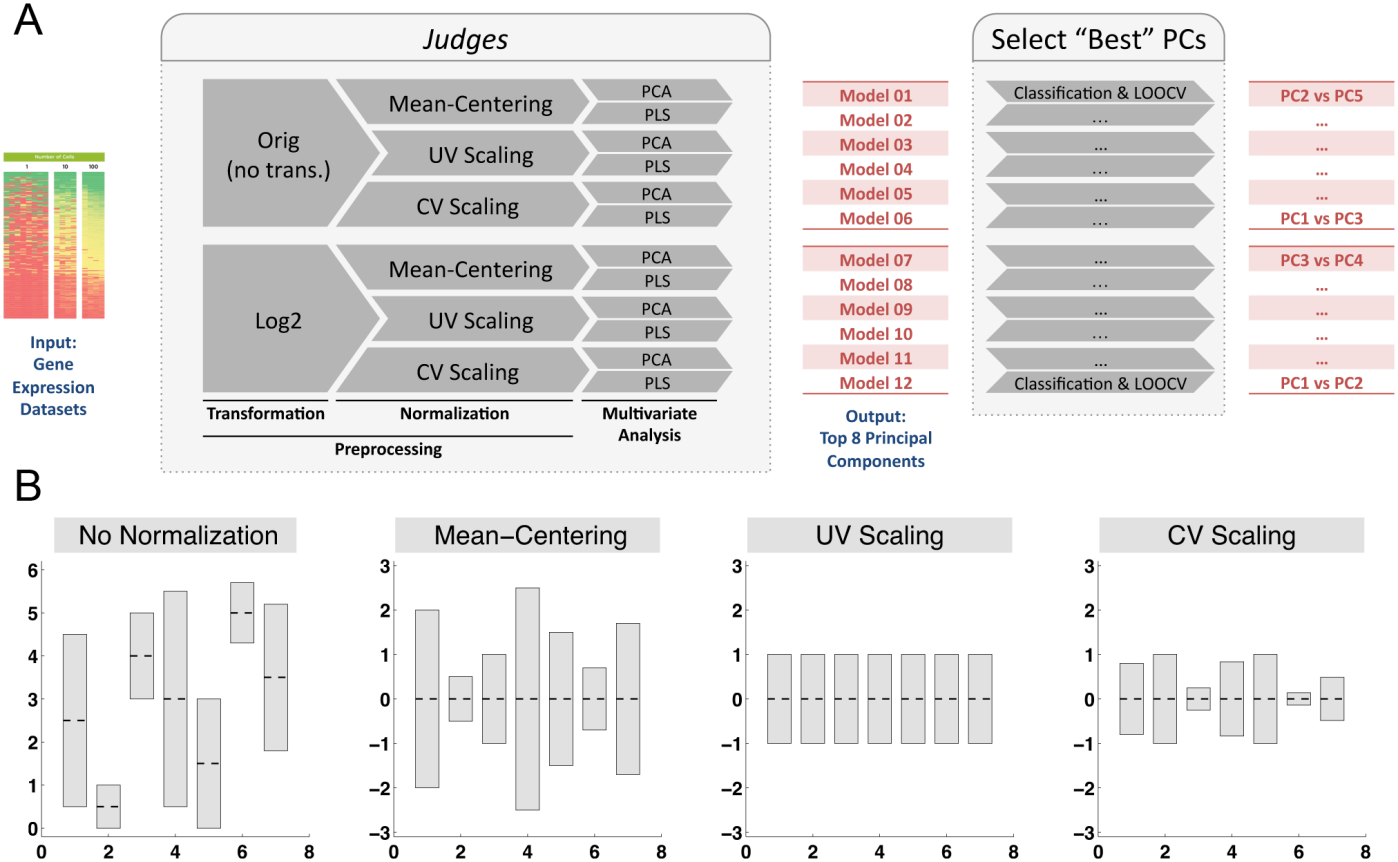
Schematic of multiplexed component analysis (MCA) algorithm for evaluating gene expression datasets. (A) Since there is no prior information on how the changes in gene expressions affect the immune response during acute SIV infection, we use an array of mathematical techniques to be able to observe the data from different viewpoints. A “*judge*” is defined as the combination of a transformation, a normalization technique and a multivariate analysis method. Each dataset is analyzed by 12 different *judges*, forming a Multiplexed Component Analysis (MCA). Each *judge* provides a model consisting of a set of principal components (PCs), which are used to classify datasets based on one of the two output variables: time since infection or SIV RNA in plasma (classification schemes). For each *judge*, the two PCs that provide the most accurate and robust classification are chosen for further analysis. (B) Normalization methods include mean-centering (MC), unit-variance scaling (UV), and coefficient of variation scaling (CV); each method results in a different representation of the data, emphasizing different characteristics of the original data set. The MC normalization method emphasizes the genes with the highest absolute variations; the UV normalization method gives equal weight to each gene in the dataset; the CV normalization method emphasizes the genes with the highest relative changes.

In this work, we present a multiplexed component analysis (MCA) technique in which we combine a variety of preprocessing techniques with two popular multivariate analysis methods to develop a set of twelve “*judges*” (Fig 1A). Preprocessing emphasizes specific features of a dataset by using an array of methods such as mean-centering, unit-variance scaling, or coefficient of variation scaling (CV), applied on the original or log-transformed data. Using a multiplexed set of preprocessing techniques ensures that we incorporate multiple possibilities for how gene expression changes affect the immune response, and therefore do not artificially include or exclude potentially significant genes. We use PCA [10,20–23] and PLS [24,25] as multivariate analysis techniques, which are powerful tools in studying datasets where the variables (88 genes) outnumber the observations (24 animals). Each of the twelve *judges* observes the data distinctively from others, and provides a set of uncorrelated principal components (PCs). We identify top contributing genes in each tissue by ranking the overall weights (loadings) of genes on the top two classifier PCs. Combining the ranking information from all the *judges*, we are able to identify genes that are consistently and statistically significantly ranked as top contributing genes. We also examine the relation between genes in the top two classifier PCs, to study the genes that co-vary together. Finally, we calculate the contribution of each gene to the classification in each tissue to evaluate whether mRNA measurements in PBMC can act as a possible surrogate of measurements in spleen and MLN.

## Results

### Data collection, preprocessing, and the twelve *judges*

In this study, we analyzed the RNA expression levels of 88 genes in spleen, mesenteric lymph node and PBMCs of macaques acutely infected with SIV. mRNA levels were quantified using Nanostring, a probe-based technique, and values were normalized by the geometric mean of four housekeeping genes (see S1 Method). The final counts were preprocessed as described next (and in more detail in S2 Method), and the preprocessed data were analyzed using PCA or PLS (more detail in S3 Method and S4 Method).

Preprocessing the data had two steps: transformation and normalization. Transformation of raw data can be advantageous when some of the variables in the dataset have extreme measurements (outliers), resulting in a non-normal distribution for these variables. The outliers may exert a large impact on the model and overshadow other measurements. For datasets with non-zero values, one method to alleviate the non-normality of the data is to perform log-transformation [26]. In this manuscript, we either use the original raw data (*Orig*) or perform log_2_-transformation on the data (*Log2*).

Normalization of the data is common because the typical amount and the range of expression for each gene in the datasets can vary substantially. This can significantly affect analyses attempting to identify which genes are key during the acute SIV infection. The type of normalization used alters the type of gene expression changes that are assumed to be significant, which in turn is related to how these gene expression changes can affect the immune response. In this work, we use three preprocessing methods: (1) Mean-centering (MC) subtracts the average value from each measurement to set the mean of the data to zero (Fig 1B). The MC normalization method emphasizes the genes with the highest absolute variations in mRNA measurements across animals; (2) Unit-variance scaling (UV) divides the mean-centered variables by their standard deviation, resulting in unit variance variables (Fig 1B). The UV normalization method is a popular method that gives equal weight to each variable in the dataset; (3) Coefficient of variation scaling (CV) divides each variable by its mean and subtracts one (Fig 1B). This gives each variable the same mean, but a variance equal to the square of the coefficient of variation of the original variable. This method emphasizes the genes with the highest relative changes in mRNA measurements. For a worked example illustrating the difference between the types of gene changes to which each normalization method is responsive, see S2 Method.

Each of our 12 *judges* is a combination of a preprocessing method (transformation and normalization) and a multivariate analysis technique, i.e. a *judge* can be represented by an ordered triple (*x*, *y*, *z*) where *x* takes its value from {*Orig*, *Log2*}, *y* takes its value from {*MC*, *UV*, *CV*}, and *z* takes its value from {*PCA*, *PLS*} (Fig 1A). Therefore, there are 12 distinct *judges* in our analysis. We use * to denote all the possible options for a particular triple element; for example, (*Log2*, *, *PCA*) defines all the *judges* that use log_2_-transformation and the PCA analysis method. In this work, the dataset for each tissue (spleen, MLN, PBMC) was analyzed by all 12 *judges*, forming the Multiplexed Component Analysis algorithm.

### Animals cluster into separate groups in the score plot

**Fig 2:**
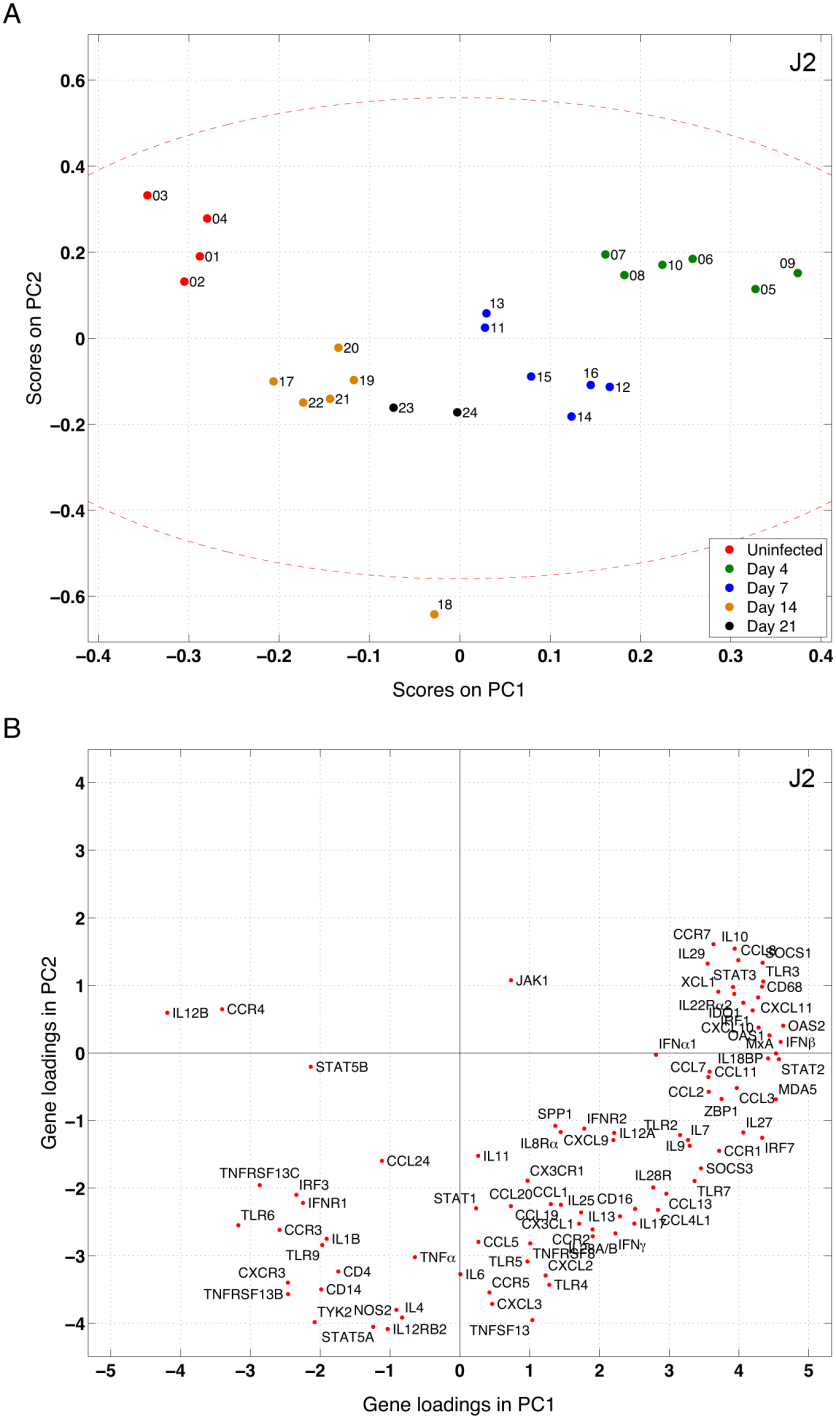
Multivariate gene expression is predictive of output variables: score and loading plots for the spleen dataset analyzed by *judge* 2—*J2*: (*Orig*, *UV*, *PCA*). (A) Score plot; each dot represents an observation (animal), projected onto PC1 and PC2. Although the PCA method is given no information on the time since infection, clearly animals cluster with their time points. The red dashed ellipse determines the 95% confidence interval, which is drawn using Hotelling's T^2^ statistic. (B) Gene loadings (weights) for the top two PCs. Genes that are highly loaded on PC1 and PC2 (i.e. far from the origin) contribute more to the scores in the score plot than other genes. Genes located in the same direction are highly correlated. The results for other *judges* testing the spleen dataset are shown in S3 Information (score plots) and Fig 3 (loading plots).

After PCA or PLS is performed on the preproccesed data, observations are projected onto a low dimensional space and are assigned new coordinates, called scores. Fig 2A shows a PC1-PC2 score plot of 24 observations (animals) in the spleen dataset analyzed by *judge* 2—*J2*: (*Orig*, *UV*, *PCA*). Together, these two principal components capture 57.5% of the variation in the dataset. Although PCA is an unsupervised method with no information on the time since infection, it is seen that dots with the same color (animals with the same time of infection) grouped together. The red dashed ellipse is drawn using Hotelling's T^2^ statistic [27] to determine the 95% confidence interval, which contains all the dots except animal #18. A circular pattern is seen in Fig 2A: uninfected animals (red dots) lie in the top left quadrant but they move to the top right quadrant 4 days after infection (green dots), and the bottom right quadrant at 7 days (blue dots), and settle in the bottom left quadrant (brown and black dots), possibly demonstrating a new steady state at 14–21 days. Fig 2B is the corresponding loading plot, where the weight of each gene on PC1 and PC2 is shown. The loading and score plots are closely linked such that genes that are highly loaded in a specific direction in the loading plot contribute more to the observations that are located in that direction in the score plot. For example, type I interferons (*IFNα1* and *IFNβ*) and interferon-stimulated genes (*MxA*, *OAS1*, *OAS2*) are rapidly and significantly upregulated during the first days of SIV infection and they share similar location with the 4-day group in the score plot. In general, genes that are far from the origin point (0, 0) in the loading plot, i.e. highly loaded on PC1 and PC2, contribute more to the scores in the score plots. The loading plots are used to find correlated genes through clustering genes that are located in a particular direction, provided that the two PCs constructing the loading plots satisfactorily approximate the matrix of the data [28]. For example, functionally related inflammatory genes such as type I interferons are located in the top right quadrant, which indicates the correlation between their expression profiles.

### The *judges* have distinct interpretations of the spleen

In this section, we focus in detail on the spleen dataset analyzed by all the 12 *judges*, as described in the methods; we applied the methods to the other tissues as well. Each *judge* emphasizes a unique type of change in gene expression, and hence the shapes of the gene clouds calculated by each *judge* are different, showing that different genes could be predicted to be significant depending on the underlying assumptions, which are different for each *judge*.

**Fig 3:**
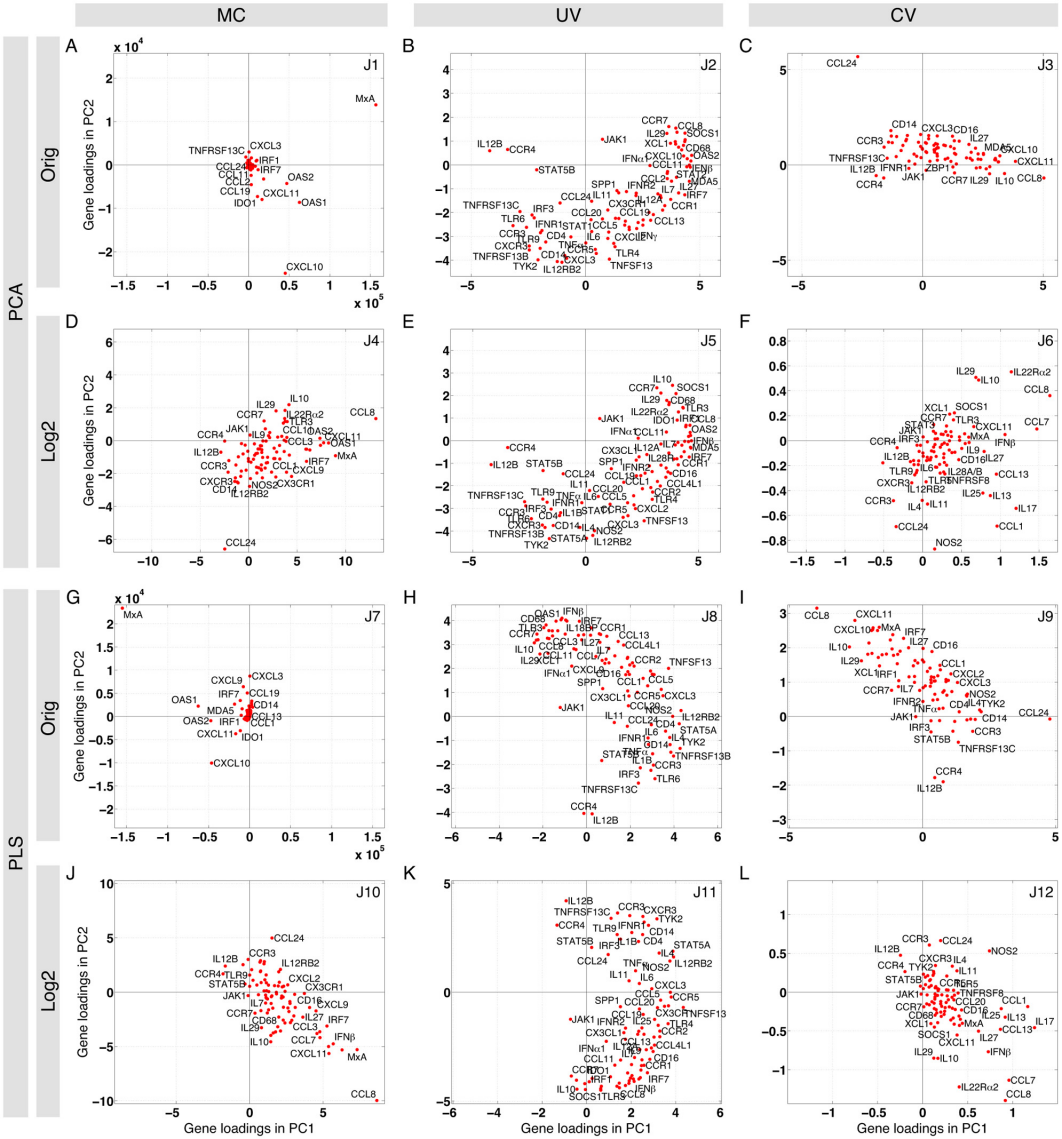
The 12 *judges* identify both consensus and *judge*-specific genes: loading plots of 12 *judges* for the spleen dataset. In our analysis, we either use the original data (the 1st and 3rd rows) or log_2_-transformed data (the 2nd and 4th rows). Before performing PCA (top two rows) or PLS (bottom two rows), preprocessing methods are applied on the data to make it mean-centered (MC), unit variance (UV)-scaled, or coefficient of variation (CV)-scaled (columns). For this figure, we use time since infection as the output variable for PLS. The loading plots on PC1 and PC2 are shown for each *judge*. In the first column (MC), we observe that there are a few highly loaded genes in each plot and the rest of genes are located in a dense cloud. In the second column (UV), there is no single gene with a significantly high loading and the cloud of genes is more spread out. In the third column (CV), we see a combination of the main features of the previous columns: a few highly loaded genes are observed and the gene clouds are more spread. The score and loading plots for the 12 *judges* in other datasets (and other classification schemes) are available in S3 Information.

In Fig 3, the loading plots for the first two components are shown for the 12 *judges*. The loading plot constructed by *J1*: (*Orig*, *MC*, *PCA*) shows both *MxA* and *CXCL10* are loaded higher than other genes. For *J7*: *(Orig*, *MC*, *PLS)*, where PCA is replaced by PLS, we see that, the gene cloud is approximately mirrored compared to the gene cloud of *judge 1*. Other high-loading genes for these two *judges* include *OAS1*, *OAS2*, *CXCL11*, and *IDO1*. Comparing no transformation (*Orig*) with log_2_-transformation (*Log2*) in *judges 1* and *4*, we observe that *judge 4* is less dominated by a small number of highly-loaded genes than *judge 1* (the scales on the axes are different). A common feature of *judges 1*, *4*, *7*, and *10* is that the MC is the normalization method. Thus, if we assume that changes in the absolute value of gene expressions have significant impacts on the immune response, genes such as *MxA*, *CCL8*, and *CXCL10* are highly contributing to the immunological events observed during acute SIV infection. Indeed, *MxA* is one of the most reliable surrogates for the measurement of type I interferon response both *in vitro* and *in vivo* [29], and *CCL8* and *CXCL10* are important chemoattractants for monocytes and activated lymphocytes, respectively [30].

Unlike (*, *MC*, *) in the 1st column, no single gene with a significantly high loading is seen in the 2nd column, constructed by (*, *UV*, *). Instead, we observe a group of genes that have higher loadings than others. This is somewhat expected since all the genes have equal variance when the UV scaling is performed. Comparing *J2*: *(Orig*, *UV*, *PCA)* and *J5*: *(Log2*, *UV*, *PCA)*, we observe that there is a slight rotation between the two gene clouds, while they are similar in terms of the distance of genes from the origin and their relative location. If PC2 in *judge 8* is multiplied by -1, the same scenario will be observed between *judges 8* and *11*, both of which use the UV normalization method. This indicates that UV scaling may alleviate the issue of non-normality and therefore log_2_-transformation has a lesser effect in this case.

The CV scaling method, used in the 3rd column, preprocesses genes to have their variance equal to the square of the coefficient of variation of the original genes. Therefore, it lies somewhere between the UV scaling method, which gives equal variance to each variable, and the MC normalization method, which does not modify the variance of variables at all. Here, we also observe that the 3rd column of *judges*, (*, *CV*, *), shares features with both the first and second columns, i.e., a few highly loaded genes as well as a spread cloud of genes. The preprocessing methods clearly impact the shape of the gene clouds constructed by PC1 and PC2, and hence changing the loading (importance) of genes under each assumption. In the next section, we define metrics to select the best pair of PCs for each *judge* to perform further analysis.

### The choice of top classifier PCs varies between the *judges*

The score plots provided by the PCA and PLS methods are used to cluster observations into separate groups based on the information on time since infection or SIV RNA in plasma. For each *judge*, dataset (tissue) and classification scheme (time since infection *or* SIV RNA in plasma), our goal is to find a score plot that provides the most accurate and robust classification of observations and to study the gene loadings in the corresponding loading plot. For each *judge*, we look at 28 score plots generated by all the combinations of two of the top eight PCs. This is because in all cases a high degree of variability, at least 76% and on average 87%, is captured by the top eight PCs (S2 Information). Next, we perform centroid-based classification and cross validation to obtain classification and LOOCV rates, indicative of the accuracy and the robustness of the classification on a given score plot, respectively. The PCs representing the highest accuracy and robustness are chosen as the top two classifier PCs for that *judge* (S2 Table). PC1 and PC2 are the most commonly chosen classifier PCs, comprising 75% and 51% of all pairs, respectively. This is expected, as PC1 and PC2 capture the highest amount of variability among PCs. The PC1-PC2 pair is chosen in 25 out of 72 cases, followed by PC1-PC3 and PC1-PC4, each chosen in 9 cases.

**Fig 4:**
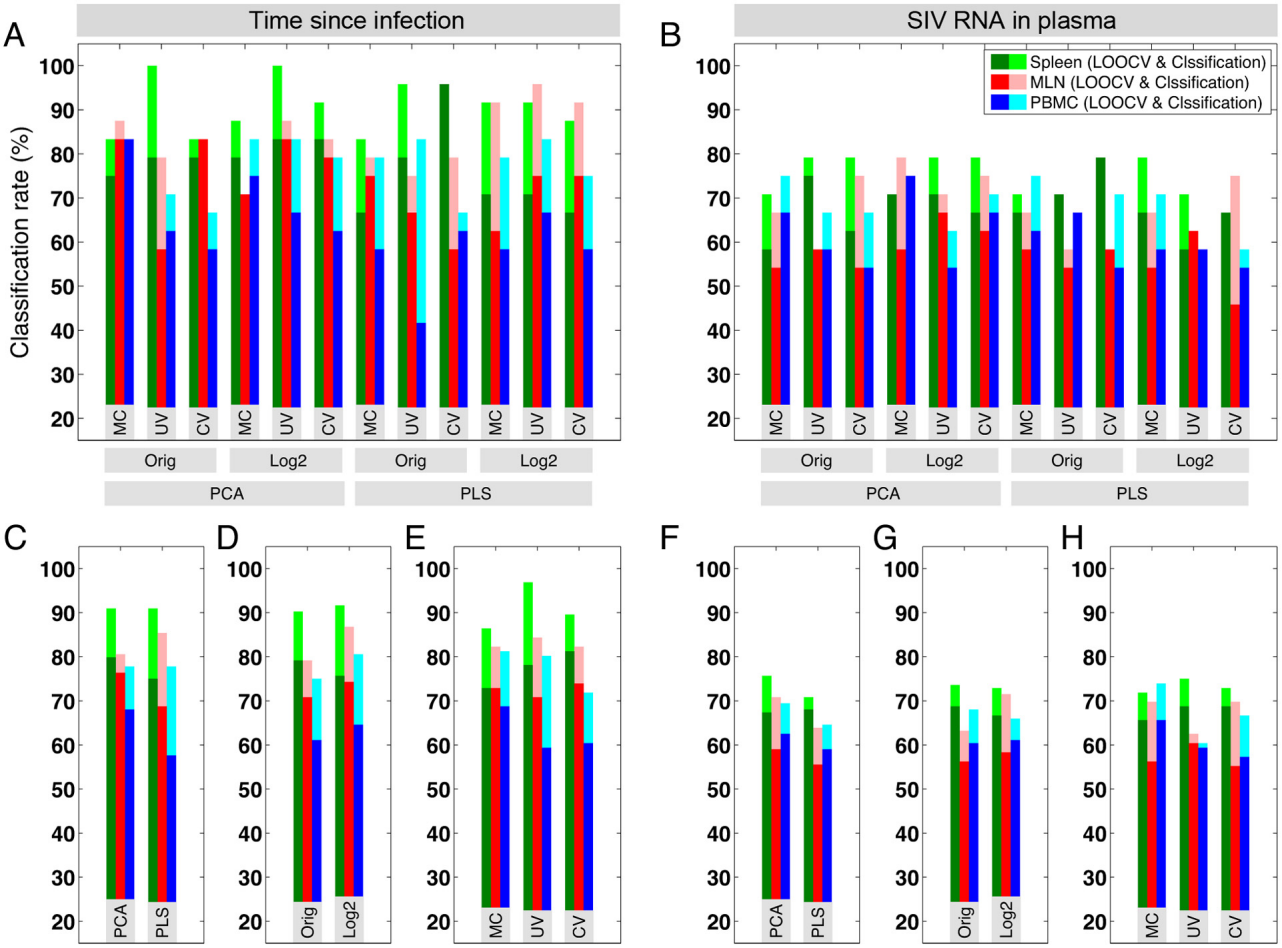
Classification and cross validation in all datasets and for both classification schemes. The classification and LOOCV rates for the top classifier PCs are shown for each *judge* for classifications based on (A) time since infection and (B) SIV RNA in plasma. Light and dark colors represent the classification and the LOOCV rates, respectively. (C-H) The average classification and LOOCV rates are also shown for *judges* using a common feature, i.e. *Orig* vs. *Log2*, *MC* vs. *UV* vs. *CV*, and *PCA* vs. *PLS*. In general, we observe that clustering based on SIV RNA in plasma is less accurate and less robust than the classification based on time since infection.

The results of clustering for both classification schemes are shown in the score plots in S3 Information and summarized in Fig 4. In most cases for time since infection (Fig 4A), the classification rates are higher than 75% (mean = 83.9%) and the LOOCV rates are higher than 60% (mean = 70.9%). For SIV RNA in plasma in most cases (Fig 4B), classification rates are higher than 60% (mean = 69.2%) and the LOOCV rates are higher than 54% (mean = 61.9%). We observe that clustering based on SIV RNA in plasma is generally less accurate and less robust than the classification based on time since infection. This may suggest that measuring SIV RNA in plasma alone does not provide a good indicator for the changes in immunological events during SIV infection due to the complex interactions between the virus and the immune system. Indeed, during HIV infection, markers for cellular activation are better predictors of disease outcome than plasma viral load [31].

In order to find whether there is a particular transformation, or preprocessing, or multivariate analysis that systematically provides more accurate and robust results than others, we calculated the average classification and LOOCV rates for *judges* that have a common feature, i.e. *Orig* vs. *Log2*, *MC* vs. *UV* vs. *CV*, and *PCA* vs. *PLS* (Fig 4C–4H). In our datasets, the overall conclusion is that each of the *judges* has merit and can outperform others in some cases. It would be difficult to argue that one *judge* is clearly better than others when we consider both classification and LOOCV rates. Since each *judge* observes the data from a distinct viewpoint and we want to consider various assumptions on how the immune response is affected by the changes in gene expressions, we combine their opinions to identify significant genes during acute SIV infection.

In general, after the classification and cross validation are performed, the *judges* need to be evaluated based on their accuracy and robustness. If a *judge* has a low accuracy compared to others, that *judge* can be removed from further analysis. Alternatively, more accurate *judges* can be given higher weights when the results are combined. In this application, all the *judges* have high and approximately similar accuracy and robustness and hence we give them equal weights when we combine the results. Note that although the *judges* have similar accuracy, each of them analyzes data differently and assigns distinguishably different loadings to the genes (loading plots in S3 Information).

### CCL8 is identified as the top “contributing” gene by all the *judges*

Genes that are highly loaded (distant from the origin) contribute more to the scores that were used for classification, and hence are considered as top “contributing” genes. To find these genes, we calculate the distance of each gene from the origin in the loading plots (loading plots in S3 Information) and rank the values with the highest rank equivalent to the maximum distance, i.e. the highest contribution. Therefore for a given dataset and a classification scheme, each gene is assigned a rank (highest ≡ 1; lowest ≡ 88) from each *judge*, resulting in a total of 12 ranks for each gene.

**Fig 5:**
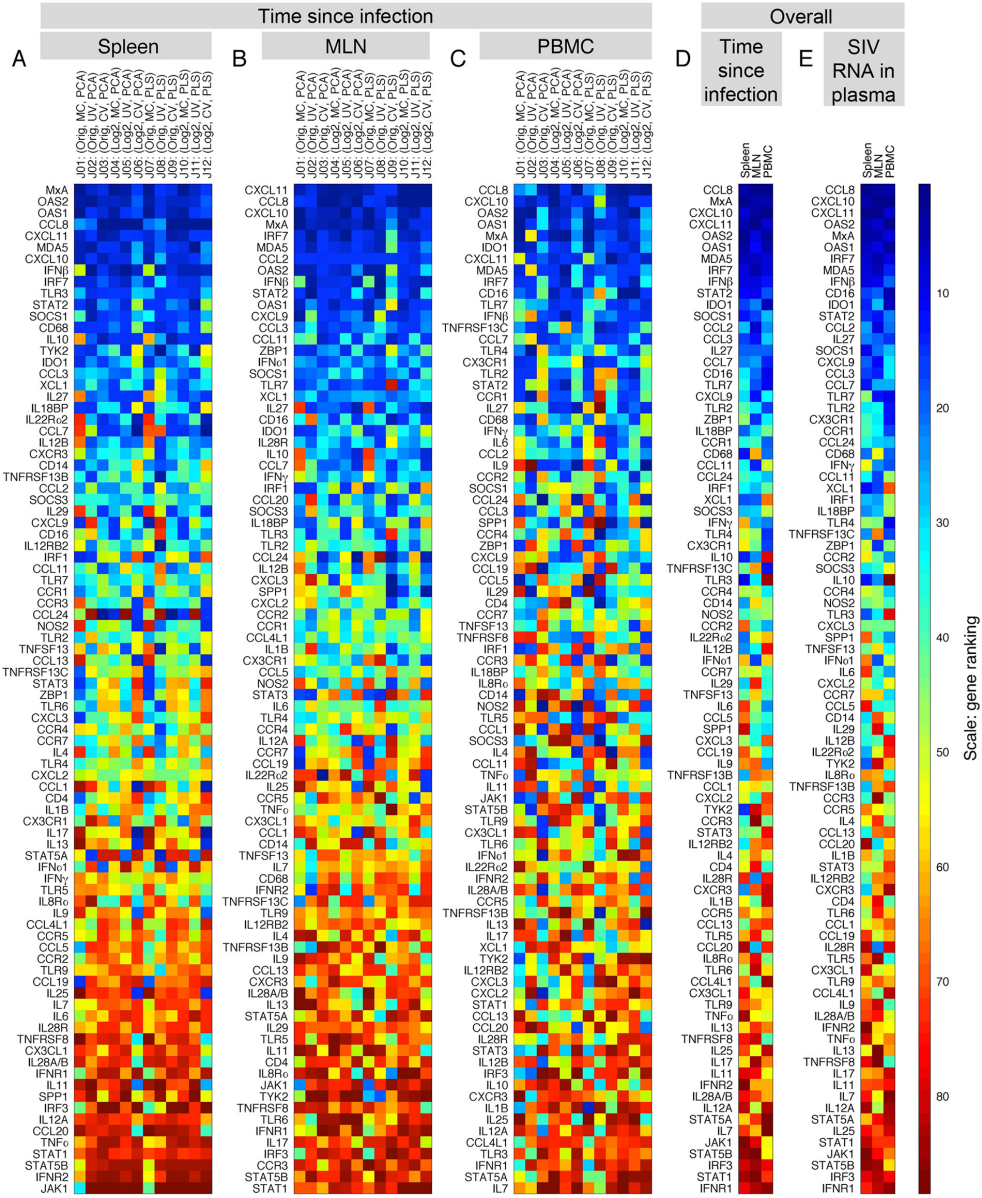
Identification of tissue-specific and global genes: gene rankings across *judges* and datasets (tissues). The highly loaded genes contribute more to the scores that are used for classification, and hence are considered as the top “contributing” genes. To study genes based on their contribution, we calculate the distance of each gene from the origin in the loading plots and rank the distance values in a descending order with the highest rank equivalent to the maximum distance, i.e. the highest contribution. For a given dataset, each gene is assigned a rank (highest ≡ 1; lowest ≡ 88) from each *judge*, resulting in a total of 12 ranks for each gene. Then, we calculate the average of twelve ranks for each gene and sort the results from the high-ranking genes (dark blue) to the low-ranking genes (dark red) in the (A) spleen, (B) MLN and (C) PBMC datasets. This leads to an overall rank for each gene in each of the datasets. (D) We calculate the average value of the three overall ranks and sort the results in a descending order of contribution. We observe that *CCL8*, followed by *MxA*, *CXCL10*, *CXCL11*, *OAS2*, and *OAS1* are ranked as the top contributing genes in all datasets. S4 Information shows the equivalent results for SIV RNA in plasma as the classifier.

The first level of analysis is whether any of the genes are ranked consistently higher or lower than the other genes, across all *judges*. To answer this, we create a 88×12 gene ranking table where rows and columns correspond to genes and *judges*, respectively. Using the Friedman test, we obtained extremely small *p*-values (S3 Table), suggesting that in all three tissues and for both classification schemes there is at least one gene that is consistently ranked higher or lower than others. The genes are sorted based on the average of their 12 ranks in Fig 5A–5C (time since infection) and panels A-C in S4 Information (SIV RNA in plasma). To find the overall contribution of genes, the genes are also sorted based on the average of their three overall ranks (Fig 5D–5E). *CCL8* is ranked as the highest contributing gene in both classification schemes. Albeit with a different order of contribution, *CCL8* is followed by *CXCL10*, *CXCL11*, *MxA*, *OAS2*, and *OAS1* in the two classification schemes. These genes always appear among the top eleven contributing genes in all tissues and for both classification schemes. These genes are all stimulated by type I interferon, suggesting that the cytokine storm we here identify in lymphoid tissues—and that is also observed in the plasma of patients during acute HIV infection—reflects a systemic innate immune response against viral replication [1,32]. While there are genes that contribute highly to all three tissues, among the transcripts analyzed in this project we cannot identify a single gene that consistently appears in the *lowest* eleven contributing genes.

To evaluate our MCA method, we compared its ranking results with those of other methods including the Pearson correlation (S5 Information), the Spearman correlation [33,34] (S6 Information), One-way analysis of variance (ANOVA) (S7 Information), and the significance analysis of microarrays (SAM) [35] (S8 Information) methods, all of which are used to rank the genes. Note that t-statistics and fold-change methods are also used in literature, but they are limited to classifications based on two groups. For each method, we selected the top five genes in each dataset and built decision trees to classify the observations using the selected genes. In most cases, the generated trees overfitted the dataset, and hence we pruned the trees and chose the sub-tree with the lowest cross validation error rate. The results indicate that, in 11 out of 12 cases, the top genes selected by MCA have substantially better classification power than those selected by the Pearson or Spearman correlation methods (panels A and C in S9 Information). The classification results of the SAM and ANOVA methods are similar to those of the MCA method. Furthermore, the Spearman's rank correlation coefficients, measuring the degree of similarity between the rankings of the MCA and other methods, indicate high correlations between the MCA and SAM methods (panels B and D in S9 Information). We also showed that in most cases the classification power top 5 average-ranked genes selected by all the *judges* is equally well or better than that of the top 5 genes selected by each individual *judge* (S10 Information) or that top 5 average-ranked genes selected by the *judges* with log2-transformation (S11 Information).

**Fig 6:**
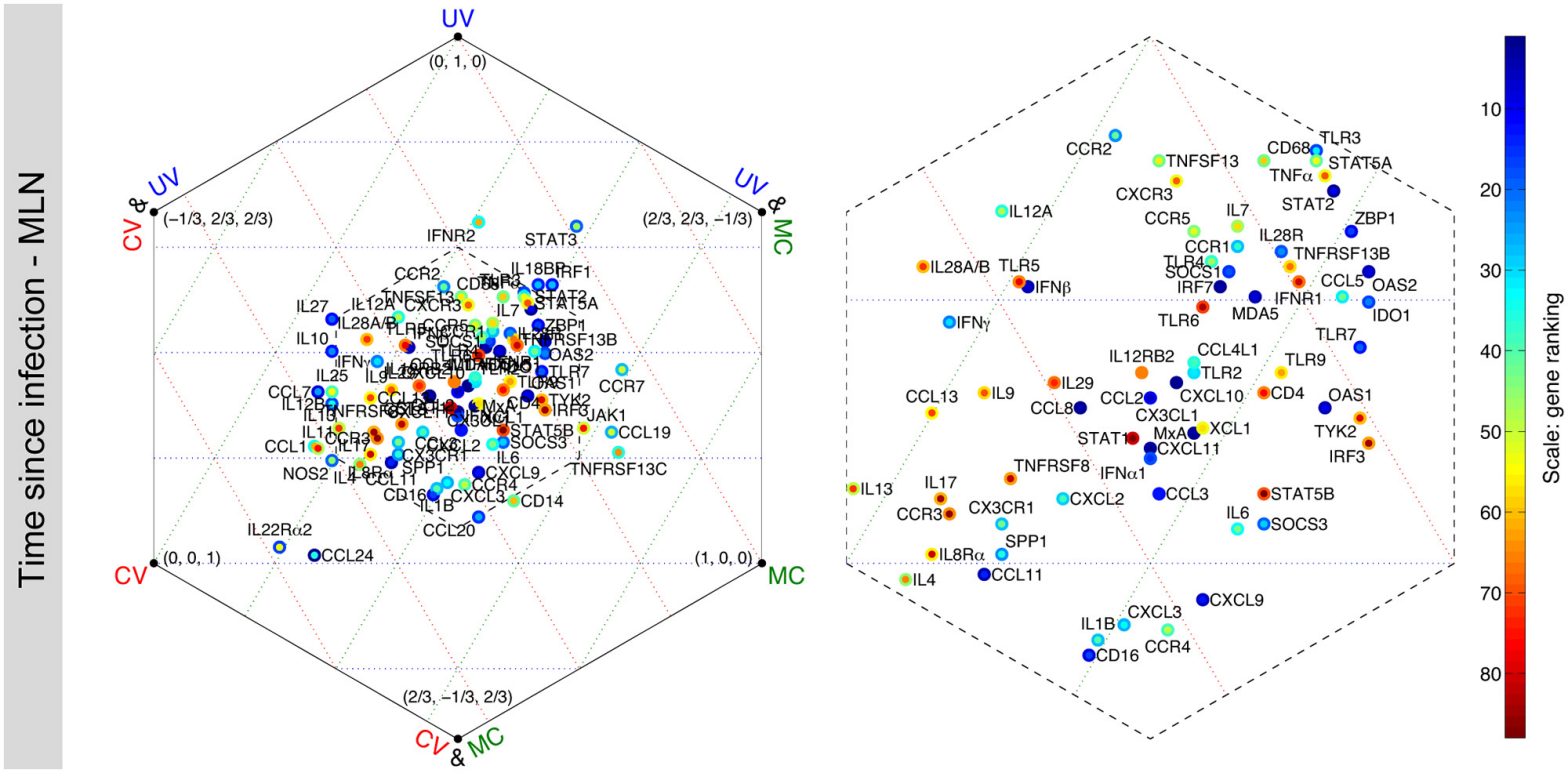
*Judge*-specificity of genes: relative importance of each gene using each normalization method, for time since infection in the MLN dataset. In each hexagonal plot, three main vertices represent MC-, UV-, and CV-based *judges*. Genes close to one of these vertices are relatively more important to that class of *judge*. Three auxiliary vertices denote CV&UV, CV&MC, and UV&MC. For example, genes that are close to CV&MC have equal importance to both CV- and MC-based *judges*. Genes at the center have approximately similar importance to each class of the *judges*. The coordinates are formatted as the relative gene importance, *C*_*UV*_, *C*_*MC*_, *C*_*CV*_, taking values in the range [-1/3, 1] and satisfy *C*_*UV*_ + *C*_*MC*_ + *C*_*CV*_ = 1 (see S6 Method for further explanation of coordinates). The inner color of each dot represents the average of the three ranks given by each class of the *judges* (obtained from Fig 5B), whereas the outer color represents the minimum (best) of the three ranks. The congested regions in the center of the left hexagonal plots are shown in greater detail on the right. Results for all tissues and classification schemes are shown in S13 Information.

The level of agreement between *judges* on the gene contributions varies substantially among genes. Similar colors across a row, such as *CXCL11* and *CCL2* in Fig 5B, show a high degree of consensus among *judges*, while there is a significant amount of disagreement between *judges* on rows with mixed colors, such as *CCL24* in Fig 5A. To measure the degree of consensus, we calculated the range and the standard deviation of the 12 ranks for each gene (S12 Information). For a given gene, there is more agreement between *judges* when both the standard deviation and the range take low values. Typically, the high contributing genes tend to be located in the left bottom corner of figures in S12 Information, suggesting that there is a high degree of agreement between *judges* on the contribution of these genes. For both classification schemes, we observe that there is a greater degree of agreement between *judges* in the MLN dataset than in spleen and PBMC. This can be visually seen in Fig 5 and the figure in S4 Information, where the gene rankings in the MLN dataset show the most consistency. Furthermore, we evaluated how genes were assigned differential rankings by the *judges* with a common feature, specifically, MC- vs. UV- vs. CV-based *judges*. The average of 4 ranks given by each class of the *judges* was calculated. This results in three ranks for each gene, representing the importance of that gene to each class of the *judges*. To identify how different *judges* analyzed the datasets, we created a metric of the relative importance of each gene (see S6 Method). The results are shown in hexagonal plots (Fig 6 and the figures in S13 Information), where genes in the center have equal importance to all three classes of the *judges*. The proximity of a gene to a vertex indicates that the gene has more importance to the class or classes of the *judges* noted at that vertex. The inner color of each dot represents the average of the ranks, whereas the outer color represents the minimum of the three ranks. The congested region in the center of the hexagon houses most of the genes and is amplified on the right-hand plot. For example, genes in the center such as *CXCL11*, *CCL8*, *CXCL10*, and *MxA* have approximately the same blue color for the inner and outer circles, showing that these genes are important to all three classes and the level of importance to each class is the same. On the other hand, *CCL24* has moderate importance when the decision of all the *judges* are combined, but it has a relatively high importance to CV-based *judges*. This suggests that *CCL24* is one of the genes with the highest amount of change relative to the mean value. Note that if a gene is only important to CV-based *judges*, then it is likely to be biologically relevant only if high relative changes are the trigger for downstream effect. Such a gene would be ignored if only UV- or MC-based methods were used.

### Gene rankings are more statistically significant in the MLN dataset

**Fig 7:**
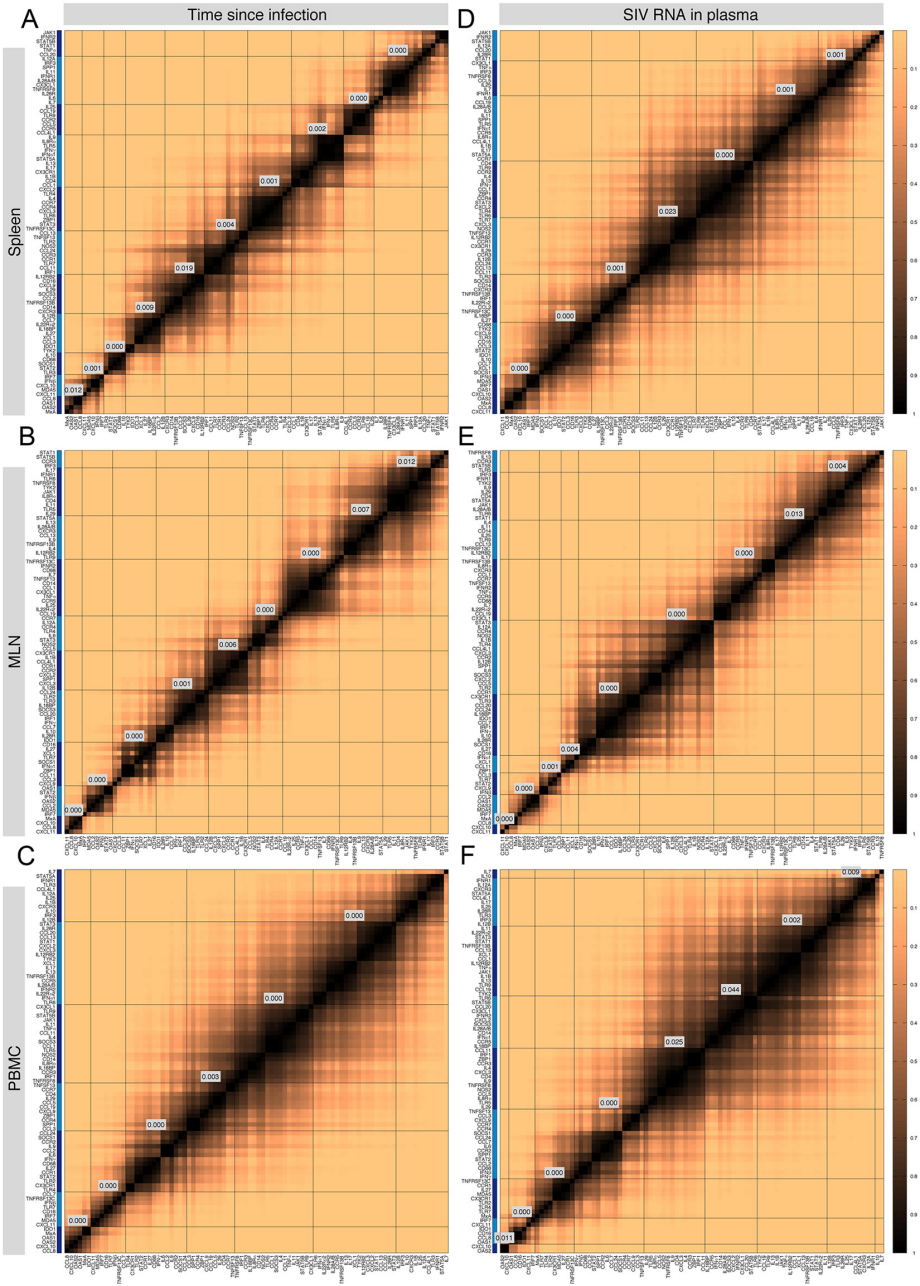
Quantifying significance of gene ranking: *p*-value heatmap of the paired t-tests of gene rankings in all datasets and for both classification schemes. In our analysis, we perform paired t-tests of gene rankings in the spleen (the 1st row), MLN (the 2nd row), and PBMC (the 3rd row) datasets. The results of the tests, *p*-values, range from 1 (black) to 0 (the light copper color). Lower *p*-values suggest a more statistically significant difference between the contribution of genes. On the bottom and left axes, genes are listed from the highest average rank (the left bottom corner) to the lowest as seen in Fig 5 and the figure in S4 Information. The clusters, colored alternately dark and light blue along the vertical axis, determine the genes that are significantly different from genes in other clusters; the labels display the *p*-value of the paired t-tests between the cluster below the label and the cluster right to it. High resolution images of the panels are shown in S14 Information.

We study the statistical significance of the gene contributions by running a paired t-test for every two rows (genes) of the 88×12 table to evaluate the null hypothesis that the two genes have equal contribution against the alternative hypothesis that one gene contributes significantly higher than the other one. If the *p*-value of the test takes sufficiently small values, it shows that one of the genes has a significantly higher contribution (Fig 7). Using linkage analysis (dendrograms), we identified clusters of genes that are statistically ranked higher than other succeeding gene clusters (α = 0.05). For example in Fig 7A, the highest contributing group of genes consists of *MxA*, *OAS2*, *OAS1*, and *CCL8*. In this group, the sharpest statistical difference is between *MxA* and *OAS1* with a *p*-value of 0.55, suggesting that none of the genes in this group are significantly more contributing than others. Similarly, in the second top contributing gene cluster, the lowest *p*-value, 0.23, belongs to the paired t-test between *CXCL11* and *IRF7*, meaning that the genes in this group are also not statistically significantly different. Instead, when we compare these two top gene clusters, we obtain a *p*-value of 0.012, meaning that the first gene cluster is significantly more contributing than the second gene cluster. For both classification schemes, the diagonal dark region for the MLN dataset is narrower than the other panels and the transition from the dark color to the light copper color is the sharpest. In agreement with our previous observations (compare Fig 5A–5C), this suggests that the gene rankings in the MLN dataset are more statistically significant than in the other two datasets. We note that *p*-values of paired t-tests between consecutive single genes did not take sufficiently small values to show statistically significant difference among them. Instead, we were able to identify gene clusters that were statistically different compared to each other. mRNA measurements from more animals could lead to lower *p*-values, smaller gene clusters and more statistically significant gene rankings. High resolution images of the panels of Fig 7 are shown in S14 Information.

### Polar plots provide a complete picture of the genes in the datasets

**Fig 8:**
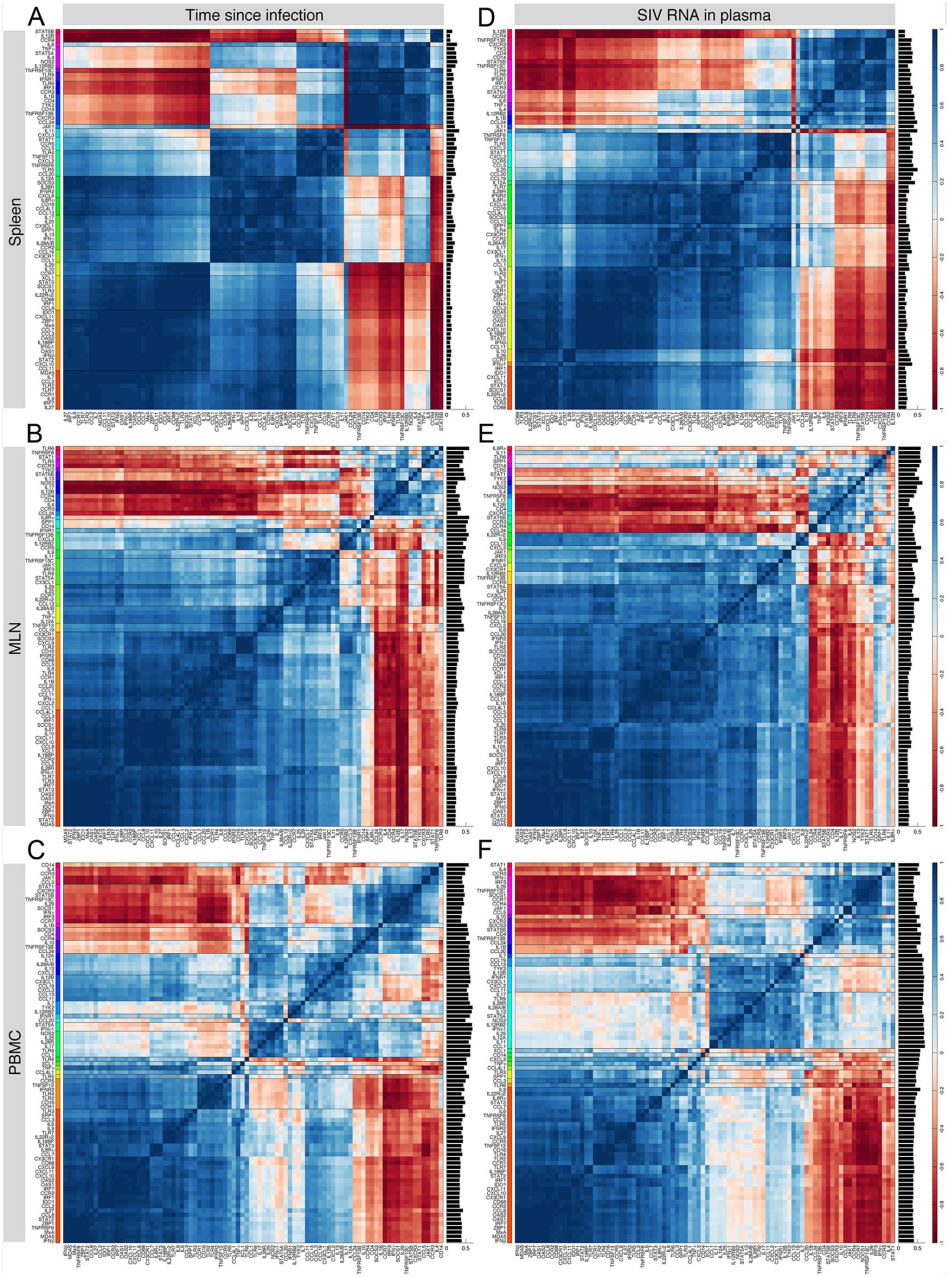
Average correlation coefficient matrices in all datasets, for both classification schemes. For each of the loading plots obtained from the 12 *judges*, we construct a matrix of correlation coefficients. Then, we calculate the average correlation coefficient matrix from the 12 matrices for a given dataset and a classification scheme. Dark blue and red colors represent positive and negative correlations, respectively, whereas light colors represent no correlation. For each pair of genes, we calculated the standard deviation of the 12 correlation coefficients, resulting in 88 values for each gene. The mean of these values, indicative of the level of agreement between *judges*, is shown in a bar chart on the right hand side of each panel. Smaller values suggest higher degrees of agreement between *judges* on the correlation of a gene with other genes. Genes that have approximately similar correlation patterns in the dataset are grouped into 20 gene clusters (shown in different colors along the vertical axis). High resolution images of the panels are shown in S15 Information.

In the loading plots, we assign a vector to each gene from the origin to its location and study the correlation between genes using the cosine of the angle between their vectors, resulting in a matrix of size 88×88 (loading plots in S3 Information). This is possible because the columns of the score matrix are orthonormal and the top two classifier PCs provide an accurate and robust classification of the observations, and hence sufficiently approximate the dataset [28]. The angular correlation coefficients obtained this way do not necessarily match the *pairwise* correlation coefficients calculated using mRNA measurements in the dataset. Instead, they are calculated in the context of all other genes on planes that closely approximate the dataset. The average of 12 correlation coefficient matrices (one for each *judge*) for a given dataset and a classification scheme is shown in Fig 8, where each row or column shows the correlation coefficients between a specific gene and other genes. For each pair of genes, we calculated the standard deviation of the 12 correlation coefficients, resulting in 88 values for each gene. The mean of these values is calculated for each gene and shown in the bar chart on the right hand side of each correlation matrix. Smaller values of the mean for a gene imply higher degrees of agreement between *judges* on the correlation of that gene with other genes. For example in Fig 8A, the *judges* have the lowest degree of consensus about the correlation of *IL11* with other genes. For both classification schemes, the *judges* have a high degree of agreement on the gene correlations in the spleen dataset (Fig 8A and Fig 8D). This is followed by the MLN and PBMC datasets, respectively. Using linkage analysis (dendrograms), we identified 20 clusters comprising genes with approximately similar correlation patterns in the dataset. Interestingly, interferon-stimulated genes (*MxA*, *OAS1*, *OAS2*) always appear in the same group and in close proximity to type I interferon genes (*IFNα1* and *IFNβ*), suggesting correlated behavior during acute SIV infection. High resolution images of the panels of Fig 8 are shown in S15 Information.

To visualize the relative position of each gene compared to the other genes, we next perform PCA on the average correlation coefficient matrix and construct the loading plot using the first two PCs scaled by the square root of their eigenvalues (S16 Information). Since the first two PCs capture more than 70% of the variance, they can create a plane that closely approximates the matrix, and hence the cosine of the angle between any two genes is approximately equal to the corresponding correlation coefficient in the matrix [28]. To validate this assumption, we calculated the angular correlation coefficients matrices from these plots, which provide a good approximation of the average correlation coefficient matrices with differences between some genes (compare Fig 8 and the figure in S17 Information). We measured the confidence on the angular position of a gene relative to others by calculating the mean-square-difference (MSD) between rows of the average correlation coefficient matrices in Fig 8 and their corresponding matrices in S17 Information. If the MSD of a gene takes small values, it suggests there is high confidence on the angular position of that gene in the loading plot.

**Fig 9:**
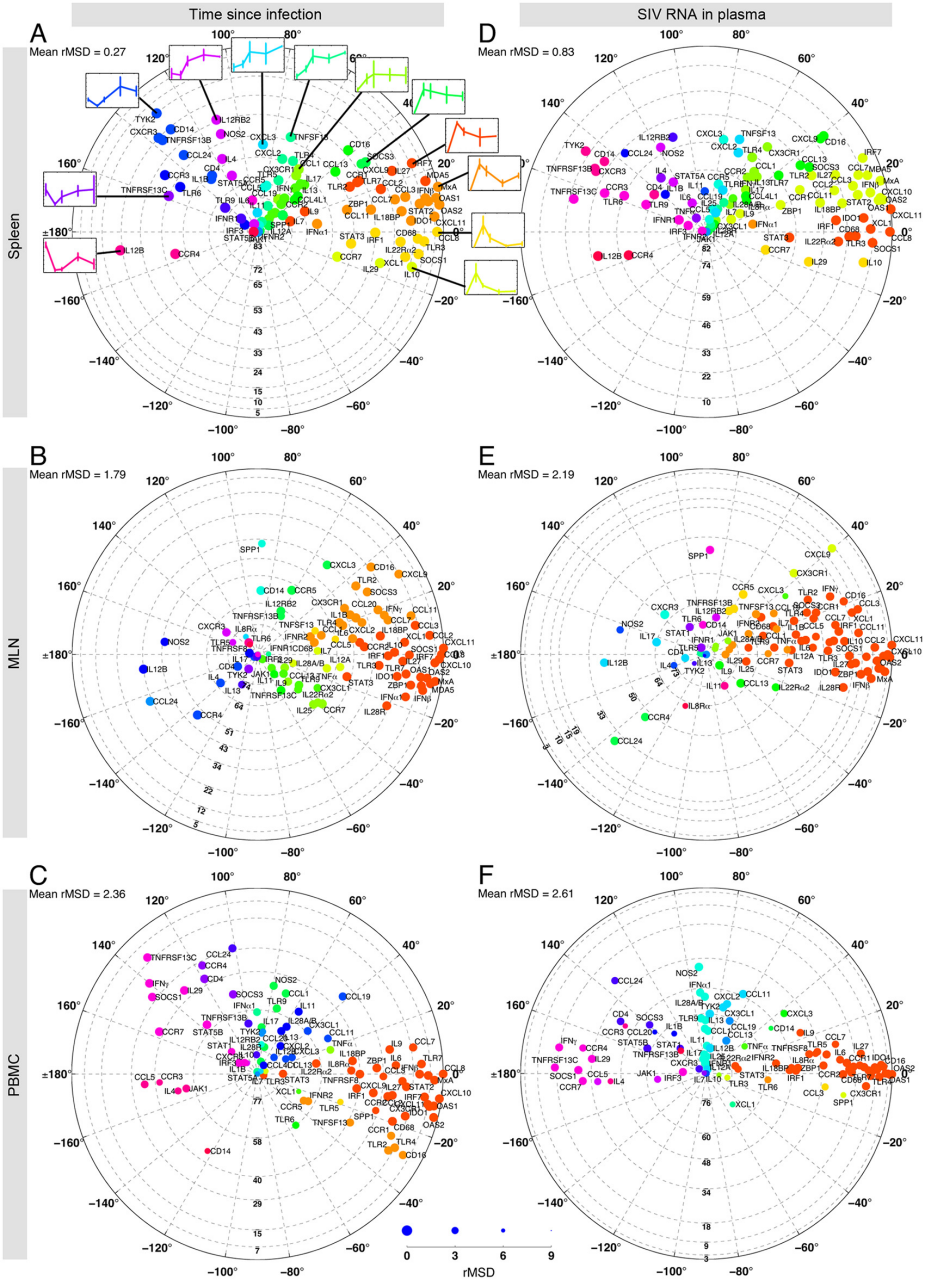
Correlations among genes simplified on a polar plot, illustrating MSD values and the ranking information. The distance from the origin indicates the overall contribution of the genes in the dataset, obtained from Fig 5 and the figure in S4 Information. Therefore the high-ranking genes are located close to the perimeter while low ranking genes are located at the center. The radial grid lines define the cluster of genes that consist of significantly more (less) contributing genes than their lower (upper) neighboring clusters. These clusters are the dark and light blue clusters shown in Fig 7. The angular position of genes is extracted from the loading plots constructed by the first two eigenvectors of the average correlation coefficient matrix (S16 Information). To make the comparisons easier, the clouds of genes are rotated such that *CCL8*, the top contributing gene, is located at zero degrees. The genes are color-coded to match the gene clusters shown on the left hand side of each panel in Fig 8. Genes with the same color show similar patterns of correlation with other genes. The radius of each dot is linearly inversely proportional to the square root of MSD. The relationship between the dot size and the value of rMSD is shown on a scale at the bottom, where the largest and smallest circles correspond to rMSD = 0 and 9, respectively. In panel A, specific examples of gene expression dynamics typical of each cluster are shown. The complete set of gene expression dynamics is available in S18 Information.

Polar plots summarize correlation information, MSD values and gene rankings in one place (Fig 9). The distance from the origin indicates the overall contribution of the genes in the dataset, obtained from Fig 5 and the figure in S4 Information. The angular position of genes is extracted from the loading plots constructed by the first two eigenvectors of the average correlation coefficient matrices (S16 Information). The radial grid lines define the clusters obtained in Fig 7, each of which contains genes that are significantly more contributing than the genes in the lower neighboring cluster. Also, genes with the same color have similar patterns of correlation with other genes (the colors match the gene clusters shown in Fig 8). We plotted the expression profiles of representative genes from these clusters, showing the dynamic mRNA expression profiles as we move around the plot. Finally, the radius of each dot is linearly inversely proportional to the square root of MSD (rMSD), i.e. there is more confidence on the angular position of larger dots. We generally observe that the dots in the spleen dataset for classification based on time since infection have a larger size compared to other cases. This is because the first and second PCs capture more than 96% of the variance in the average correlation coefficient matrix (panel A in S16 Information). The complete gene expression profiles are shown in S18 Information. Polar plots overview the information that can be obtained for any given gene from previous figures. For example, we observe that in the Spleen, MLN, and PBMC datasets, *CCL8*, *MxA*, *CXCL10*, *CXCL11*, *OAS2* and *OAS1* are located close to the perimeter in the top two clusters, meaning that they are all top contributing genes and their contribution is statistically significantly higher than that of other genes; they are clearly grouped in the same angular direction, suggesting strong correlations exist among them; they are all represented by large dots, implying high confidence on their locations relative to the other genes; and they are upregulated during the first days of SIV infection and their expression goes down after 4 days p.i.

Note that we could not directly combine the information on the angular position of genes in the loading plots provided by the *judges*. This is because if a PC is multiplied by -1, the new vector is still a principal component; however, all the relative positions of genes change in the loading plot. To avoid this problem, we converted the information on the angular position of genes to the correlation coefficients for each *judge*, took the average of the correlation coefficient matrices and converted it back using PCA to visualize positions of genes relative to each other. A schematic of the algorithm for obtaining polar plots is given in S19 Information.

### Top contributing genes have approximately equal contributions to all tissues

**Fig 10:**
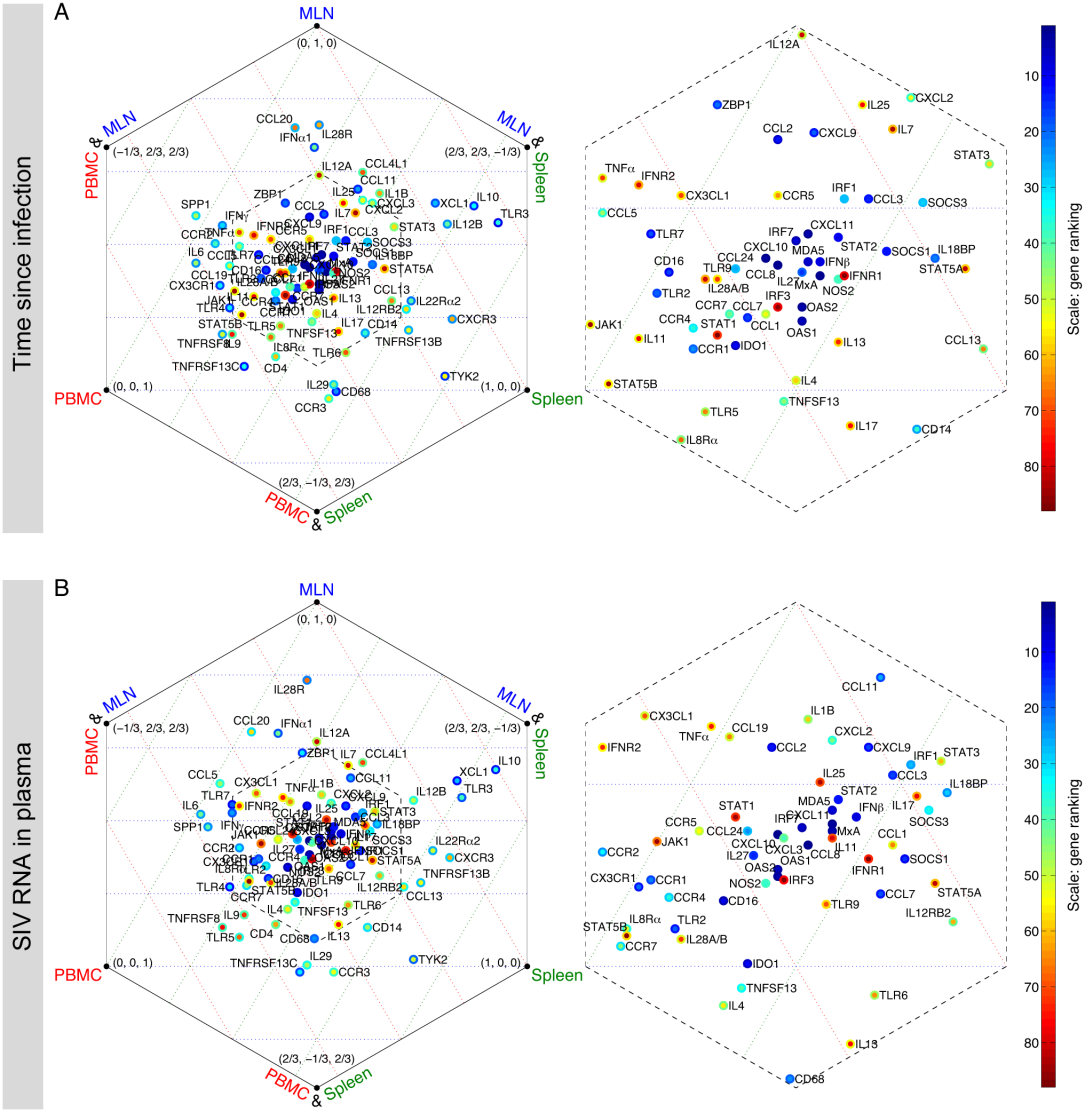
Tissue-specificity of genes: relative contribution of each gene to each tissue. In each hexagonal plot, three main vertices represent Spleen, MLN, and PBMC. Genes close to one of these vertices show a strong contribution to the corresponding tissue. Genes at the center contribute approximately equally to each tissue. The inner color of each gene shows its overall rank in all tissues (Fig 5D–5E), while the outer color represents the minimum of each gene’s three ranks in the tissues.

Since genes contribute differently to each tissue, we measure the relative contribution of each gene to identify tissue-specific genes (see S6 Method). The results are shown in hexagonal plots (Fig 10), where genes in the center contribute equally to all tissues. The proximity of a gene to a vertex indicates that the gene contributes more to the tissue(s) noted at that vertex than to other tissues. The inner color of each dot represents the average contribution of the gene, whereas the outer color represents the highest contribution (lowest rank) of that gene. The common genes are seen close to the center of the hexagon, while the tissue-specific genes are located close to the vertices and near the edges. The congested region in the center of the hexagon houses most of the genes. To see this region more clearly, it is amplified on the right-hand plot. For both classification schemes, we observe the top contributing genes such as *CCL8*, *MxA*, *CXCL10*, *CXCL11*, *OAS2*, and *OAS1* lie in the center of the plot with approximately the same blue color for the inner and outer circles, indicating their equal contribution to all tissues (Fig 10). This suggests that type I interferon responses are quite similar in the three compartments and that these genes could be used as biomarkers to be measured in PBMCs instead of spleen and MLNs during acute SIV infection. This can be tested by classifying the observations using the mRNA measurements of these genes in PBMCs and by evaluating whether that classification is as accurate as the classifications using measurements in spleen or MLN. To this end, we built decision trees using the top seven highly contributing genes and chose the sub-trees with the lowest cross validation error rates in all tissues and for both classification schemes (S4 Table). For time since infection and SIV RNA in plasma, the classification rates in the PBMC dataset are 87.5% and 83.3%, greater than or equal to the classification rates in spleen and MLN. This suggests that an analysis of gene expression in the more accessible PBMC can be used as a surrogate to understand the immunological events happening in the less accessible spleen and lymph nodes during acute SIV infection. However, each tissue has unique expression profiles, e.g. *XCL1*, a relatively high-contributing gene, contributes highly to spleen and MLN compared to PBMC, and hence analysis of selected top contributing tissue-specific genes could greatly inform about the mechanisms related to SIV infection in those tissues.

## Discussion and Conclusions

Acute HIV infection is characterized by an exponential increase in plasma viremia with subsequent viral dissemination to lymphoid and non-lymphoid organs. As the innate immune system responds to viral replication, the expression of inflammatory cytokines in the plasma also rapidly increases, leading to a positive feedback where newly-induced activated cells allow for more viral infection. This hypercytokinemia is known as "cytokine storm", and it is not unique to HIV [36]. Other pathogens may also cause strong immune responses that lead to tissue damage, organ dysfunction and death. For instance, severe acute lung injury with respiratory failure can be observed after SARS-CoV and influenza infections, and are caused by cytokine storms in the lung alveoli and peripheral blood [36]. In HIV infection, this inflammatory response is not fatal but may cause irreparable impairment to the immune system, leading to massive CD4+ T cell depletion and chronic immune activation [1]. A similar cytokine storm is observed during acute infection in the brain of SIV-infected macaques [2], indicating that even immune-privileged organs are not shielded from the damage that such responses may cause during HIV and SIV infection. Understanding the pathways and components of these immunological events is essential for the development of therapeutic strategies aimed at reducing their harmful effects. Similar acute phase studies cannot be performed in HIV-infected patients for several reasons, including lack of precision regarding the exact time of transmission, limited access to organ biopsies, and HIV genotypical diversity [37]. Therefore, SIV macaque models represent a viable and efficient alternative to human studies, despite the biological differences between HIV and SIV [3,38].

In this study we used an accelerated and consistent macaque model of AIDS and HIV-associated neurocognitive disorders to analyze the expression of immune-related genes in three different lymphoid compartments during acute SIV infection. mRNA levels were quantitated by Nanostring, a novel technology that allows for the measurement of a large number of transcripts without reverse transcription or DNA amplification. Fluorescent bar-coded probes specifically hybridize with mRNAs that are then counted by a powerful scanner. The technique involves little sample manipulation and generates results faster, presented in a simple spreadsheet format. The Nanostring panel in this study was designed to understand how immune responses are longitudinally developed in different organs or cells during SIV infection. The panel includes genes that are commonly analyzed during inflammation and viral infection, and has been used to evaluate the longitudinal level variances in individual cytokines during SIV infection. Thus, the panel gives us insight into the host response to acute infection.

Studies that attempt to analyze changes of gene expressions over time or only examine bivariate correlations between two genes or a gene and a clinical parameter such as SIV RNA in plasma can result in limited (and often flawed) conclusions. This can be due to several reasons including lack of prior information on how changes in gene expressions affect the immune response, noisy measurements, and contribution of many genes, each of which has a minor impact but when considered together can create a significant response. In addition, after animals are infected by SIV, the changes in gene expressions are presumably caused by SIV infection. One can expect the mRNA measurements, regardless of their biological functions, to be correlated with SIV clinical parameters. This suggests that drawing conclusions based on only bivariate correlations can be misleading. Therefore, multivariate analysis techniques are more appropriate tools to study a set of genes simultaneously.

Here, we introduced a novel multiplexed component analysis (MCA) technique to simultaneously analyze mRNA measurements under different assumptions for how the gene expression changes affect the immune response during acute SIV infection. In this method, mRNA measurements were studied by 12 *judges*, each of which consists of three successive modules: 1) transformation (*Log2* or *Orig*), 2) preprocessing (*MC*, *UV*, or *CV*) and 3) multivariate analysis (*PCA* or *PLS*). The preprocessing module aims to emphasize specific features of the dataset, e.g. the MC normalization method emphasizes biological responses in which the immune response is affected by the genes with the highest absolute variations in expression across animals, whereas the CV normalization method puts emphasis on responses in which the relative changes in gene expressions are more important. Note that other normalizations, transformations and multivariate techniques could be combined to create more *judges*; however, the goal in this method is to have unique *judges* that observe the data from distinct viewpoints and hence the techniques that have similar effects on the data should not be included in the same analysis.

Each of the twelve *judges* provides a distinct set of uncorrelated principal components (PCs), capturing the directions in the data with maximum variance. From each set, we select two PCs that provide the most accurate and robust classification of the data in each of the classification schemes: time since infection and SIV RNA in plasma. These selection criteria result in PCs with gene loadings that robustly classify the animals at different stages of the disease. Our hypothesis is that highly loaded genes, which contribute the most to the classification, are those whose levels of expression are most profoundly affected during acute SIV infection and therefore warrant further study. While the MCA technique does not by itself provide mechanistic insight into how these genes function in the immune system, it provides an impartial platform to compare genes and highlight those with the highest level of contribution during acute SIV infection, globally in the immune system or locally in specific tissues; and it can further be combined with mechanistic information about the immune response dependence on specific gene expression changes. Also, the MCA method can be used in genome-wide studies, where the number of genes is significantly higher. The transformation and normalization modules do not change in such applications. Also, the PCA and PLS methods are essentially dimension reduction algorithms and hence can be readily applied to large datasets to identify genes with significant contributions. One should note that the sets of significant genes selected by individual *judges* might be different when the number of genes is high, and hence extra attention should be paid when the gene ranking results from the judges are combined. In our datasets, the top eight PCs were enough to capture more than 76% of the variation within the dataset. When the number of genes increases, more than eight PCs may be needed to capture sufficient variance within the dataset.

We can combine the opinions of all the *judges* to sort genes based on their overall rank. As discussed above, the *judges*’ agreement on the gene rankings differs for each gene. When there is a high level of agreement among the *judges* for a gene, it suggests that the gene is accurately ranked, regardless of how the changes in gene expressions affect the immune response. On the other hand, there are genes that receive high ranks from some *judges* and low ranks from the others. This suggests that the specific way that gene expression changes are translated to the immune response matters, and that these genes can hold less or more significance, which in turn generates new hypotheses for future experiments. The results also demonstrate differential ranking of some genes according to specific lymphoid compartments. *IFNα1*, for instance, is highly ranked in MLN but not in PBMCs or spleen. We hypothesize that this is due to the highly abundant population of *IFNα*-producing dendritic cells, which are responsible for antigen presentation and T cell activation in lymph nodes [39]. Similarly, *CD68*, a bona fide marker for macrophage activation ranks higher in spleen, an organ rich in macrophages [40]. An important point to make is that all three tissues here analyzed comprise mobile cell types, and therefore are subject to numerical changes in cell subpopulations during infection. Thus, changes in gene expressions do not reflect only transcription modulation, but also cell trafficking. Interestingly, three of the highest-ranking genes, *CCL8*, *CXCL10* and *CXCL11*, are chemoattractants of cells susceptible to SIV infection (*CCL8* for monocytes and *CXCL10* and *CXCL11* for activated lymphocytes) [41,42], and may be directly responsible for the trafficking of SIV-infected cells to organs and subsequent establishment of viral reservoirs during acute infection. Similar multi-gene analyses of cell type-specific transcripts may lead to methods for the precise quantitation of leukocytes in lymphoid compartments, and their contribution to inflammatory responses during pathological conditions.

One of the main advantages of our methodology is to provide a diverse set of perspectives on the evaluation of cellular and molecular events during infection in different tissues. For instance, gene-ranking analysis informs about the overall aspects of the immune response, but also identifies signature genes that are singularly relevant to cellular mechanisms in specific lymphoid compartments. In this report, similar high ranking genes in spleen, MLN and PBMC reveal a systemic and concomitant type I interferon response during acute SIV infection, despite the diversity in cell populations in each tissue and the particular pathways by which cell phenotypes respond to viral infection. Therefore, the synchronous changes in gene expressions appear to be driven mostly by the crosstalk between cells and cytokines that are constantly trafficking through tissues than by viral replication *per se* [32].

Nonetheless, ranking gives somewhat limited information on how genes relate to each other and how transcription is longitudinally modulated in each tissue. Therefore, by combining the information on the angular position of genes provided by all the *judges* and depicting the results in polar plots (Fig 9), it is possible to identify genes with similar regulation patterns and evaluate whether these same genes are equally regulated in other lymphoid compartments. As an example, all putative interferon-stimulated genes (ISG) are grouped together in all three compartments, indicating a common regulatory process. On the other hand, based on the spleen results alone, it could be suggested that the transcription activator *STAT5A* is directly involved in the regulation of *IL4*, but this is not observed in the other two tissues, suggesting either tissue-specific regulation or an aliasing effect, and these are computationally-derived hypotheses for further study.

Finally, this methodology allows for the combination of results from three related but independent analyses into one cogent hexagonal plot (Fig 10), displaying the relative contribution of each gene to the overall changes in each compartment. This powerful visualization tool can be used to identify genes that uniquely and significantly contribute to immune responses in specific tissues, and also genes that could be selected as general inflammatory markers to be investigated during acute infection. The model suggests that evaluation of a small selected panel of ISGs and chemokines in PBMCs may be sufficient to assess systemic inflammatory responses triggered by viral infection in secondary lymphoid tissues. On the other hand, *IL10* and *XCL1* appear to be highly significant in spleen and MLN but not in PBMCs, and therefore examining the levels of these cytokines in the blood may not provide accurate information regarding immunological events in lymphoid organs. The expression profiles of these genes in spleen and MLNs are strongly correlated, while they have little to no correlation with the expression profiles in PBMCs.

Note that mRNA samples in our study were isolated from different animals euthanized at 4, 7, 14, and 21 days post infection. Therefore, the obtained measurements at various time points do not constitute a longitudinal study. For example, the gene expression data points at days 4 and 7 are not inherently connected, but instead represent samples from populations of animals infected with SIV for 4 and 7 days, respectively. Therefore, the data at day 4 cannot be readily used to predict the gene expressions at day 7. In addition, there is a fundamentally different relationship between the input variables (mRNA measurements) and each of the two classification schemes. While time since infection as an output variable is intrinsically independent of the mRNA measurements, SIV RNA in plasma is completely dependent on the changes in gene expressions, as both inflammatory response genes and SIV are constantly in direct or indirect interactions in the immune system and hence cause changes in mRNA counts and SIV RNA in plasma. This, in addition to other factors, may partially explain why classification based on time since infection is more accurate than classification based on SIV RNA in plasma.

For most viral infections, the acute phase is a time of drastic physiological and immunological changes, especially at the beginning of adaptive immune responses. Further similar studies performed in samples collected at later time points, when infection is already established, would help to evaluate the relationship between cytokine expression and viral replication.

## Methods

### Animals and ethics statement

All animal studies were approved by the Johns Hopkins University Institutional Care and Use Committee (IACUC protocol #PR12M310), and all procedures followed the guidelines of the Weatherall Report, the USDA Animal Welfare Act, and the Guide for the Care and Use of Laboratory Animals. Twenty-four juvenile pigtailed macaques (*Macaca nemestrina*) were studied before and during acute SIV infection. Twenty of these animals were dual-inoculated with the molecular clone SIV/17E-Fr and the immunosuppressive swarm SIV/DeltaB670, as previously described [43]. Groups of six infected macaque were euthanized at 4, 7, and 14 days post infection (p.i.), and two animals were euthanized at 21 days p.i. Four animals were mock-inoculated and used as uninfected controls. The 4-day time point is included as the earliest time point that the virus could be found in the brain of infected macaques. All animals were considered SPF (specific pathogen free) before enrolling in our study, and were tested negative for SIV, Simian type D retrovirus, Herpesvirus simiae (B virus), Simian foamy virus and simian T-cell leukemia virus. Uninfected and infected macaques were housed in different rooms to prevent cross-contamination. Assigned veterinarians and trained technicians monitored the animals twice daily for signs of distress, including diarrhea, weight loss, and opportunistic infections, in order that early humane endpoints could be performed if necessary. Macaques were housed in facilities that are fully accredited by the Association for the Assessment and Accreditation of Laboratory Animal Care, International (AAALAC), and fed a balanced commercial macaque chow (Purina Mills). Throughout the experiments, animals were housed in cages providing 6 square feet of space with visual and auditory contact of conspecifics, and received environmental enrichment, such as manipulanda and novel foodstuffs. Before euthanasia, all macaques were perfused with sterile saline solution. Euthanasia was performed under veterinary supervision using an overdose of intravenous sodium pentobarbital while under deep ketamine sedation (10 mg/kg intramuscular), followed by perfusion with 1X PBS prior to tissue harvest. Organs, including spleen and mesenteric lymph node (MLN), were harvested, sectioned, and frozen at -80°C. Blood was collected for the isolation of peripheral blood mononuclear cells (PBMC) using a Percoll protocol (GE Healthcare Life Sciences, Pittsburgh, USA), and cell pellets were frozen and stored at -80°C.

### RNA isolation from spleen, MLN, and PBMC

Total RNA was isolated from frozen spleen, MLN, and PBMCs using the RNeasy kit (Qiagen, Valencia, CA, USA), according to the manufacturer’s protocol. Samples were eluted in 60 μL of RNase-free water and frozen at -80°C until time to be analyzed.

### Quantification of SIV virions in plasma

Plasma was collected during euthanasia, and SIV RNA was isolated using the QIAamp Viral RNA Mini Kit (Qiagen). Analysis was performed by qPCR as previously described [44].

### NanoString nCounter gene expression system

Nanostring technology uses molecular "barcodes" (a string of fluorescent dyes that uniquely identify a specific transcript) and potent microscopic imaging to quantify genes of interest after a hybridization protocol. The technique does not require reverse transcription or DNA amplification, and provides high reproducibility and sensitivity for the detection of multiple transcripts [45]. CodeSets for 92 macaque genes (S1 Table), including four housekeeping genes, were designed according to the company's specifications, based on rhesus macaques (*Macaca mulata*) annotated sequences. In addition to the target-specific CodeSets, the kit also includes six positive probes for quality control and seven negative controls whose sequences were obtained from the External RNA Controls Consortium and are confirmed to not hybridize with mammalian genes. Isolated RNA was quantitated by spectrophotometry, and 250 ng of each sample was sent for hybridization and consecutive quantitation to the Johns Hopkins Deep Sequencing and Microarray Core. RNA counts were normalized by the geometric mean of four housekeeping genes: *actin*, *GAPDH*, *HPRT*, and *PBGD*. Therefore, we used mRNA measurements from 88 genes as input variables in our analysis (for additional information see S1 Method). The data sets supporting the results of this article are available in the NCBI Gene Expression Omnibus (GEO) database, [ID: GSE51488, http://www.ncbi.nlm.nih.gov/geo/query/acc.cgi?acc=GSE51488].

### Preprocessing of data, multivariate analysis methods, and the *judges*

The gene expression datasets are first preprocessed using a transformation and a normalization method (as described in the Results section and in S2 Method). We analyze each preprocessed set of data, using both Principal Component Analysis (PCA) and Partial Least Squares regression (PLS). For PCA, we use the *princomp* function in Matlab. The two important outputs of this function are: 1) the loadings of genes onto each PC, which are the coefficients (weights) of the genes that comprise the PC; and 2) the scores of each PC for each observation, which are the projected data points in the new space created by PCs. We impose orthonormality on the columns of the score matrix obtained by the *princomp* function and scale the columns of the loading matrix accordingly such that the score matrix multiplied by the transposed loading matrix still results in the original matrix of the data. This is necessary to study the correlation between genes in the dataset in a loading plot, provided that the two constructing PCs closely approximate the matrix of the data [28].

PLS regression is a method to find fundamental relations between input variables (mRNA measurements) and output variables (time since infection or SIV RNA in plasma) by means of latent variables called components [24,25]. In this work, we use the *plsregress* function in Matlab to perform PLS regression. This function returns PCs (loadings), the amount of variability captured by each PC, and scores for both the input and output variables. The columns of the score matrix returned by the *plsregress* function are orthonormal. Therefore one can study the correlation between genes in the dataset using the gene loadings in the loading plots. Additional information about PCA and PLS can be found in S3 Method and S4 Method.

We define a *judge* as the combination of a preprocessing method (transformation and normalization) and a multivariate analysis technique (Fig 1A), as described in the Results section. In this work, each dataset, i.e. spleen, MLN, or PBMC, was analyzed by all 12 *judges*, forming a Multiplexed Component Analysis algorithm. Instructions on how to download the Matlab files for visualization and the MCA method can be found in S5 Method.

### Classification and cross validation

In our analysis, we use a centroid-based clustering technique. We use two variables to cluster the animals into distinct groups: (1) time since infection; and (2) SIV RNA in plasma (copies/ml) (panel D in S1 Information). These variables thus define the 'classification schemes' discussed in the text. In score plots, we calculate the cluster centroids as averages of the scores within clusters. Observations are classified into clusters using shortest distance between observation and centroid. The true class information for each observation is known prior to the analysis, and if the cluster is assigned correctly, the classification is successful; otherwise it has failed. We perform the classification for all the 24 observations and calculate the classification rate.

For classification, all the data is used to train the model and the same data is classified into clusters. It is critical to measure the robustness of the classifiers to predict unknown observations. To do this, the dataset is divided into two sub-datasets: “training” and “test”. We use leave-one-out cross validation (LOOCV), in which a single observation from the dataset is selected as the test dataset. The remaining observations are used as the training dataset to build the model and to calculate the centroid for each cluster. Then, the test observation is projected onto the low-dimensional space created by the model and assigned to the nearest cluster. If the cluster is assigned correctly based on the prior class information, classification has succeeded; otherwise it has failed. We perform the cross validation for all 24 observations and calculate the estimated LOOCV rate.

## Supporting Information

S1 Method NanoString nCounter gene expression system. (DOCX)

S2 Method Normalization methods. (DOCX)

S3 Method Principal component analysis (PCA). (DOCX)

S4 Method Partial least squares (PLS) regression. (DOCX)

S5 Method Matlab codes. (DOCX)

S6 Method Visualizing the relative contribution of genes in hexagonal plots. (DOCX)

S1 Information Cytokine levels and SIV RNA in plasma versus time since infection. (DOCX)

S2 Information Percentage of variance captured by the top PCs. (DOCX)

S3 Information Score plots, loading plots and results of classification. (DOCX)

S4 Information Gene rankings across datasets for classification based on SIV RNA in plasma. (DOCX)

S5 Information Gene rankings by the Pearson correlation method. (DOCX)

S6 Information Gene rankings by the Spearman correlation method. (DOCX)

S7 Information Gene rankings by the one-way ANOVA method. (DOCX)

S8 Information Gene rankings by the significance analysis of microarrays (SAM) method. (DOCX)

S9 Information Classification results and evaluation of ranking similarity for the Pearson correlation, Spearman correlation, ANOVA, SAM and MCA methods. (DOCX)

S10 Information Classification results for the individual *judges*. (DOCX)

S11 Information Classification results for the *judges* with log2-transformation. (DOCX)

S12 Information Consensus plots showing the degree of agreement among *judges* on the contribution of genes in all datasets and for both classification schemes. (DOCX)

S13 Information *Judge*-specificity of genes: relative importance of each gene using each normalization method. (DOCX)

S14 Information *p*-value heatmap of the paired t-tests of gene rankings in all datasets and for both classification schemes (high-resolution images of the panels of Fig 7). (DOCX)

S15 Information Average correlation coefficient matrices in all datasets, for both classification schemes (high-resolution images of the panels of Fig 8). (DOCX)

S16 Information The loading plots of average correlation coefficient matrices shown in Fig 8. (DOCX)

S17 Information The correlation coefficient matrices obtained from the loading plots in S16 Information. (DOCX)

S18 Information Gene expression profiles in all datasets and for both classification schemes. (DOCX)

S19 Information Schematic of algorithm to plot polar plots in Fig 9. (DOCX)

S1 Table List of genes measured by Nanostring technology. (DOCX)

S2 Table List of top two classifier PCs for each of the *judges*. (DOCX)

S3 Table Friedman test results for gene rankings. (DOCX)

S4 Table Classification results using the top seven highly contributing genes. (DOCX)
