# Supplementary material for: Multiplexed Component Analysis to Identify Genes Contributing to the Immune Response during Acute SIV Infection
Source: PLoS One. 2015 May 18;10(5):e0126843. doi: 10.1371/journal.pone.0126843 (PMC4436129; doi:10.1371/journal.pone.0126843)
Supplement: S1 Method — (DOCX) [file pone.0126843.s001.docx]

# Method S1. NanoString nCounter® gene expression system

Note that the four housekeeping genes used in our experiments have measurements that are orders of magnitude different: Actin (mean = 97053.6), GAPDH (24761.6), HPRT (1056.3), and PBGD (259.2). And therefore in this case, use of geometric mean is more appropriate than the arithmetic mean or median, which are suited when variables take relatively similar values. In the PBMC dataset, we replaced the measurements taking a value of zero with a value of one after normalization. These include CCL11(1), CCL24(1), CX3CL1(1), IL9(2), IL13(1), IL28A/B(1), and NOS2(1). The number in parentheses represents the number of modified measurements out of 24 measurements for each gene. The modifications avoid problems caused by log-transformation on mRNA counts equal to zero. In the spleen and PBMC datasets, we observed few measurements taking values lower than the average value of the negative controls. Although we did not change these measurements, it is worth mentioning the genes with such features. These include CCL7(1), IL13(1) and IL17(1) in the spleen dataset and CCL1(1), CCL24(2), IL11(1), IL12A(1), IL13(1), IL25(2), IL9(1), NOS2(2), SPP1(1) in the PBMC dataset. The number in parentheses represents the number of measurements taking values higher than zero and lower than the average value of the negative controls.
