# Supplementary material for: Multiplexed Component Analysis to Identify Genes Contributing to the Immune Response during Acute SIV Infection
Source: PLoS One. 2015 May 18;10(5):e0126843. doi: 10.1371/journal.pone.0126843 (PMC4436129; doi:10.1371/journal.pone.0126843)
Supplement: S2 Method — (DOCX) [file pone.0126843.s002.docx]

# Method S2. Normalization methods

Mean-centering (MC) repositions the mean of the data to zero, by subtracting the average value of the variable from each measurement. If the data is not mean-centered before PCA/PLS, the first PC will be approximately in the direction of the mean of the data instead of being in the direction of maximum variance. Thus, genes that have similar distributions with different mean values will be equivalent after MC is performed on the dataset (Fig. 1B). In the next sections, we will discuss that PCA and PLS attempt to find directions in the data with maximum variance. Therefore, in the MC-preprocessed datasets, genes with a higher variance tend to get higher loadings than other genes, i.e. these genes will be farther from the origin point (0, 0) in the loading plots, and therefore contribute more to the scores in the score plots. The MC normalization method emphasizes the genes with the highest absolute variations in mRNA measurements across animals.

The MC method may overestimate the influence of variables with a larger variance in the PCA/PLS models. To give equal weight to each variable in the model, the UV scaling method divides each mean-centered measurement by the standard deviation of the variable, resulting in unit variance for all the variables (Fig. 1B). With UV scaling, the importance of higher-variance variables is reduced, while that of lower-variance variables is increased. Thus while the impetus to use the UV method is to avoid skewing the analysis towards high-variance variables, this method introduces its own skewing. As PCA and PLS extract the direction of maximum variance, it would be difficult to predict what genes would get the highest loadings in the PCA/PLS models.

When the MC normalization method is used, the assumption is that changes in the absolute value of mRNA measurements affect the immune response. In contrast, when UV scaling is performed, the range of measurements for each variable is completely ignored and all the variables are given the same weight in modeling. This implies that no variable can dominate other variables in the model due to its large variance. To find a middle ground between these two cases, we apply another preprocessing method where each variable is divided by its mean. Then we subtract one from the results to make the data mean-centered (Fig. 1B). In this case, each new variable will have a variance equal to the square of the coefficient of variation of the original variable. The coefficient of variation can be viewed as a normalized measure of variation because it measures the variability in the data with regard to the mean value. In our work, this method is called coefficient of variation (CV) scaling. The method emphasizes the genes with the highest amount of change relative to the mean value and will generally lead to a higher loading in the PCA/PLS models for genes with a high coefficient of variation.

We use an example to clarify the difference between the normalization methods. Let’s assume that we have gene expressions for three genes. Depending on the normalization method, these genes can be of less, more or equal importance compared to others.

Gene A: [ 90, 100, 110]

Gene B: [990, 1000, 1010]

Gene C: [900, 1000, 1100]

If the data is normalized by the MC method, Genes A and B will have equal importance and their relative changes will be smaller than that of Gene C.

Gene A: [ -10, 0, 10]

Gene B: [ -10, 0, 10]

Gene C: [-100, 0, 100]

If the data is normalized by the CV method, Genes A and C will have equal importance and their relative changes will be larger than that of Gene B.

Gene A: [ -0.1, 0, 0.1]

Gene B: [-0.01, 0, 0.01]

Gene C: [ -0.1, 0, 0.1]

If the data is normalized by the UV method, Genes A, B, and C will all have equal importance.

Gene A: [-1, 0, 1]

Gene B: [-1, 0, 1]

Gene C: [-1, 0, 1]

As seen, the importance of a change in gene expression can be interpreted differently by different normalization methods.
