# Supplementary material for: Multiplexed Component Analysis to Identify Genes Contributing to the Immune Response during Acute SIV Infection
Source: PLoS One. 2015 May 18;10(5):e0126843. doi: 10.1371/journal.pone.0126843 (PMC4436129; doi:10.1371/journal.pone.0126843)
Supplement: S3 Method — (DOCX) [file pone.0126843.s003.docx]

# Method S3. Principal component analysis (PCA)

PCA is a mathematical method used to convert an original set of correlated variables into a smaller set of uncorrelated variables, called principal components (PCs). One way to calculate PCs is to apply singular value decomposition (SVD) on the matrix of the measurements. PCA is a popular tool for applications such as dimension reduction and information extraction in complex datasets. The first PC captures the largest [variability](https://en.wikipedia.org/wiki/Variance) (the major trend) in the data. Each subsequent PC is then orthogonal to the previous PCs and captures the largest variability remaining in the data. Therefore, each succeeding PC has a lower amount of information (variance) compared to the preceding PCs. Depending on the application, one can remove PCs that capture low amount of information (variability) to reduce the dimensionally of the data. We impose orthonormality on the columns of the score matrix obtained by the *princomp* function in Matlab and scale the columns of the loading matrix accordingly such that the score matrix multiplied by the transposed loading matrix still results in the original matrix of the data. This is necessary to study the correlation between genes in the dataset using the genes loadings in a loading plot. In addition, the two PCs constructing the loading plot must be a good approximation of the matrix of the data [1]. This normalization of the score matrix is in agreement with the PLS method introduced in the next section.

# References

1. Gabriel KR (1971) The biplot graphic display of matrices with application to principal component analysis. Biometrika 58: 453-467.
