# Supplementary material for: Multiplexed Component Analysis to Identify Genes Contributing to the Immune Response during Acute SIV Infection
Source: PLoS One. 2015 May 18;10(5):e0126843. doi: 10.1371/journal.pone.0126843 (PMC4436129; doi:10.1371/journal.pone.0126843)
Supplement: S4 Method — (DOCX) [file pone.0126843.s004.docx]

# Method S4. Partial least squares (PLS) regression

PLS regression is a method to find fundamental relations between input (predictor) variables and output (dependent) variables by means of latent variables called components [1,2]. PLS regression is an extension of PCA where it simultaneously projects both the input and output [variables](http://en.wikipedia.org/wiki/Predicted_variable) into a low dimensional space and finds a linear regression between the results. In this work, the input variables are the gene expressions (mRNA measurements) and the output variable, also known as the classification scheme, is either time since infection or SIV RNA in plasma. Unlike PCA, which only finds directions with major variations in the input space, PLS maximizes the covariance between the input and output variables, i.e. it finds directions in the input space that correspond to the directions with major variations in the output space. Therefore, PCA and PLS are called unsupervised and supervised methods, respectively. Compared to the standard regression, PLS regression is useful in cases where the number of observations (24 animals) is smaller than the number of input variables (88 genes). The columns of the score matrix returned by the *plsregress* function in Matlab are orthonormal. Therefore one can study the correlation between genes in the dataset using the gene loadings in the loading plots.

# References

1. Wold S, Sjöström M, Eriksson L (2001) PLS-regression: a basic tool of chemometrics. Chemometrics and Intelligent Laboratory Systems 58: 109-130.

2. Wold S, Ruhe A, Wold H, Dunn I, WJ (1984) The collinearity problem in linear regression. The partial least squares (PLS) approach to generalized inverses. SIAM Journal on Scientific and Statistical Computing 5: 735-743.
