# Supplementary material for: Multiplexed Component Analysis to Identify Genes Contributing to the Immune Response during Acute SIV Infection
Source: PLoS One. 2015 May 18;10(5):e0126843. doi: 10.1371/journal.pone.0126843 (PMC4436129; doi:10.1371/journal.pone.0126843)
Supplement: S5 Method — (DOCX) [file pone.0126843.s005.docx]

# Method S5. Matlab codes

The Matlab files for visualization and the implementation of our MCA method are available at <http://gforge.icm.jhu.edu/gf/project/mca_siv/docman/?subdir=140>. We used the source code in [1] to draw the polar plots. We modified the source code in [2] to create the hexagonal plots.

# References

1. Hanselman D (2012) Comprehensive Polar Plots. MATLAB Central File Exchange. Retrieved Mar. 21, 2013 (<http://www.mathworks.com/matlabcentral/fileexchange/38855)>.

2. Sandrock C (2007) Ternplot. MATLAB Central File Exchange. Retrieved Feb. 15, 2013 (<http://www.mathworks.com/matlabcentral/fileexchange/2299)>.
