# Supplementary material for: Multiplexed Component Analysis to Identify Genes Contributing to the Immune Response during Acute SIV Infection
Source: PLoS One. 2015 May 18;10(5):e0126843. doi: 10.1371/journal.pone.0126843 (PMC4436129; doi:10.1371/journal.pone.0126843)

# Method S6. Visualizing the relative contribution of genes in hexagonal plots

In a general scenario, each gene has three different overall ranks, ranging between 1 and *L* = 88. These three ranks could represent the “*importance*” to each class of the *judges* or represent the gene’s “*contribution*” to each tissue. The two cases follow the same mathematical formalism; here we will focus on the latter case, in which the three ranks are denoted by (*R_S_*, *R_M_*, *R_P_*), representing the gene’s contribution to spleen, MLN and PBMC, respectively.

Since genes contribute differently to each tissue, we measure the relative contribution of genes to identify which ones are tissue-specific and which ones contribute equally to all tissues. To do so, first we examine two extreme cases: 1) if *R_S_* = *R_M_* = *R_P_* for a particular gene, that gene is contributing equally to all tissue; 2) if *R_S_* = 1, *R_M_* = 88, and *R_P_* = 88 for a gene, this means that particular gene fully contributes to spleen, while it has very small contributions to MLN and PBMC. In addition, note that two hypothetical genes ranked (1, 10, 10) and (71, 80, 80) should not have the same relative contributions. While both genes have a higher contribution to spleen than MLN or PBMC, the relative contribution of the former is higher than the latter. We propose the following equations to convert gene overall ranks to relative gene contributions to each tissue.

|  |
| --- |
|  |

Where *R_M_* represents the baseline contribution of a gene to all tissues. The terms *R_M_* – *R_X_* and *C_X_* represent the specific contribution and the relative contribution to tissue *X*, respectively. Note that *C_S_* + *C_M_* + *C_P_* = 1 (the term *L* – 1 in the denominator ensures that
-2/3 ≤ *r_x_* ≤ 2/3). If *C_S_* = *C_M_* = *C_P_* = 1/3, this means that the gene is contributing equally to all tissues. The farther that a gene gets from (1/3, 1/3, 1/3), there is more contribution to one tissue and less contribution to the other two. For example, (1, 88, 88) and (1, 1, 88) will be respectively converted to (1, 0, 0) and (2/3, 2/3, -1/3) using the mathematical formulation. We plot the relative gene contributions on a hexagonal plot, where (1/3, 1/3, 1/3) is the center of the hexagonal. The hexagonal plot has three main vertices representing Spleen, MLN, and PBMC and three auxiliary vertices denoting PBMC&MLN, PBMC&Spleen, and MLN&Spleen. The arrows in figure below show the directions in which the relative contribution of a gene decreases for each of the main vertices. For example, as we move from the main vertex Spleen to the auxiliary vertex PBMC&MLN, the relative contribution to spleen decreases linearly from 1 to -1/3. Grid lines are drawn perpendicular to the arrows and are used to help calculate the coordinates for a given point. To do so, one should draw parallel lines to the grid lines, find the intersection of those lines with the corresponding arrows and then calculate the distance from the corresponding main vertices.


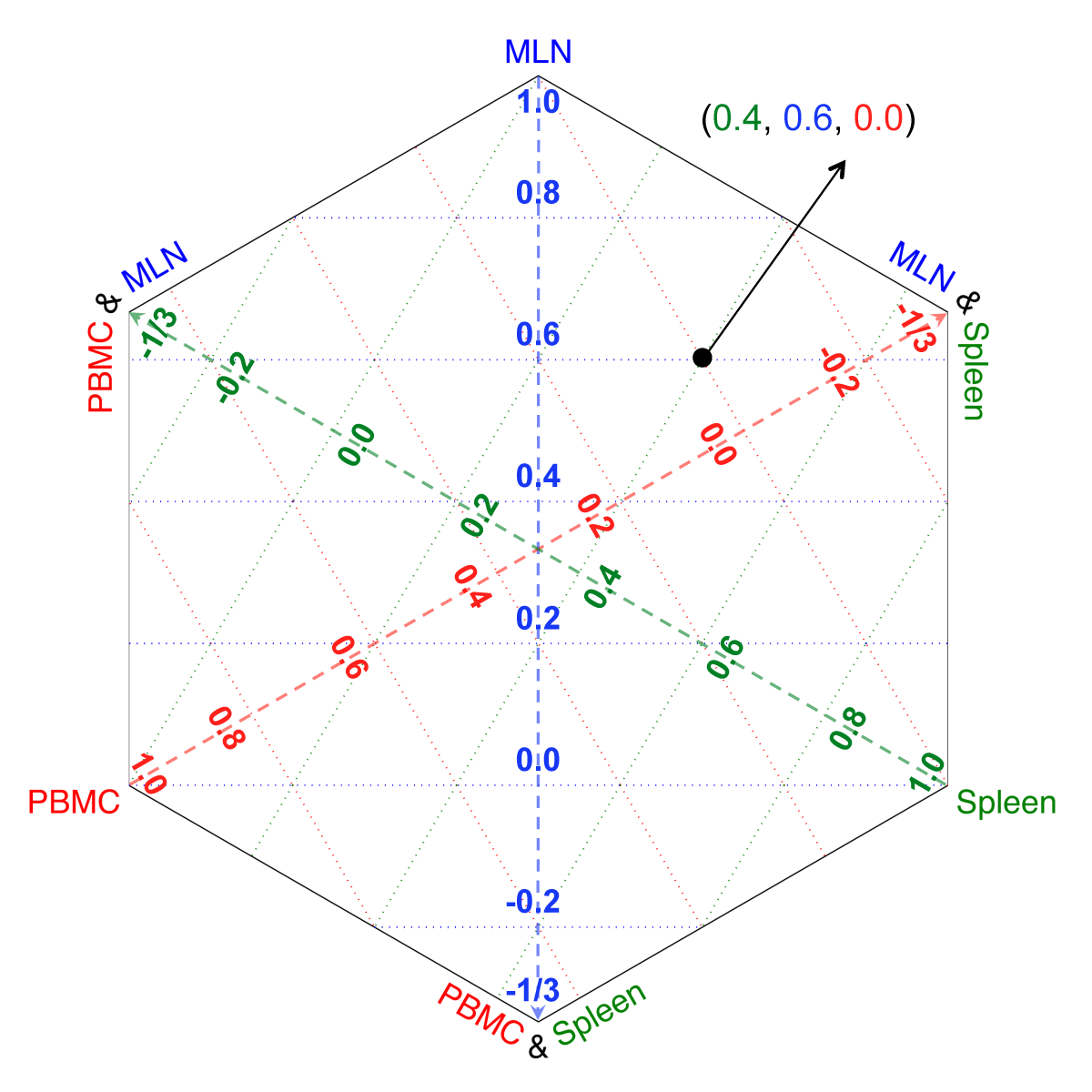

Supplement: S6 Method — (DOCX) [file pone.0126843.s006.docx]
