# Supplementary material for: Multiplexed Component Analysis to Identify Genes Contributing to the Immune Response during Acute SIV Infection
Source: PLoS One. 2015 May 18;10(5):e0126843. doi: 10.1371/journal.pone.0126843 (PMC4436129; doi:10.1371/journal.pone.0126843)
Supplement: S1 Information — (DOCX) [file pone.0126843.s007.docx]

# Figure S1. Cytokine levels and SIV RNA in plasma versus time since infection

The mean changes of cytokine mRNA levels in (A) Spleen, (B) MLN, (C) PBMC, relative to baseline in uninfected animals over time in groups of animals acutely infected with SIV. We used the cubic spline method to interpolate between the data points. Note that mRNA samples in our study were isolated from different animals euthanized at various time points and hence do not constitute a longitudinal study. (D) Viral load in plasma at 4, 7, 14, and 21 days post infection.

**
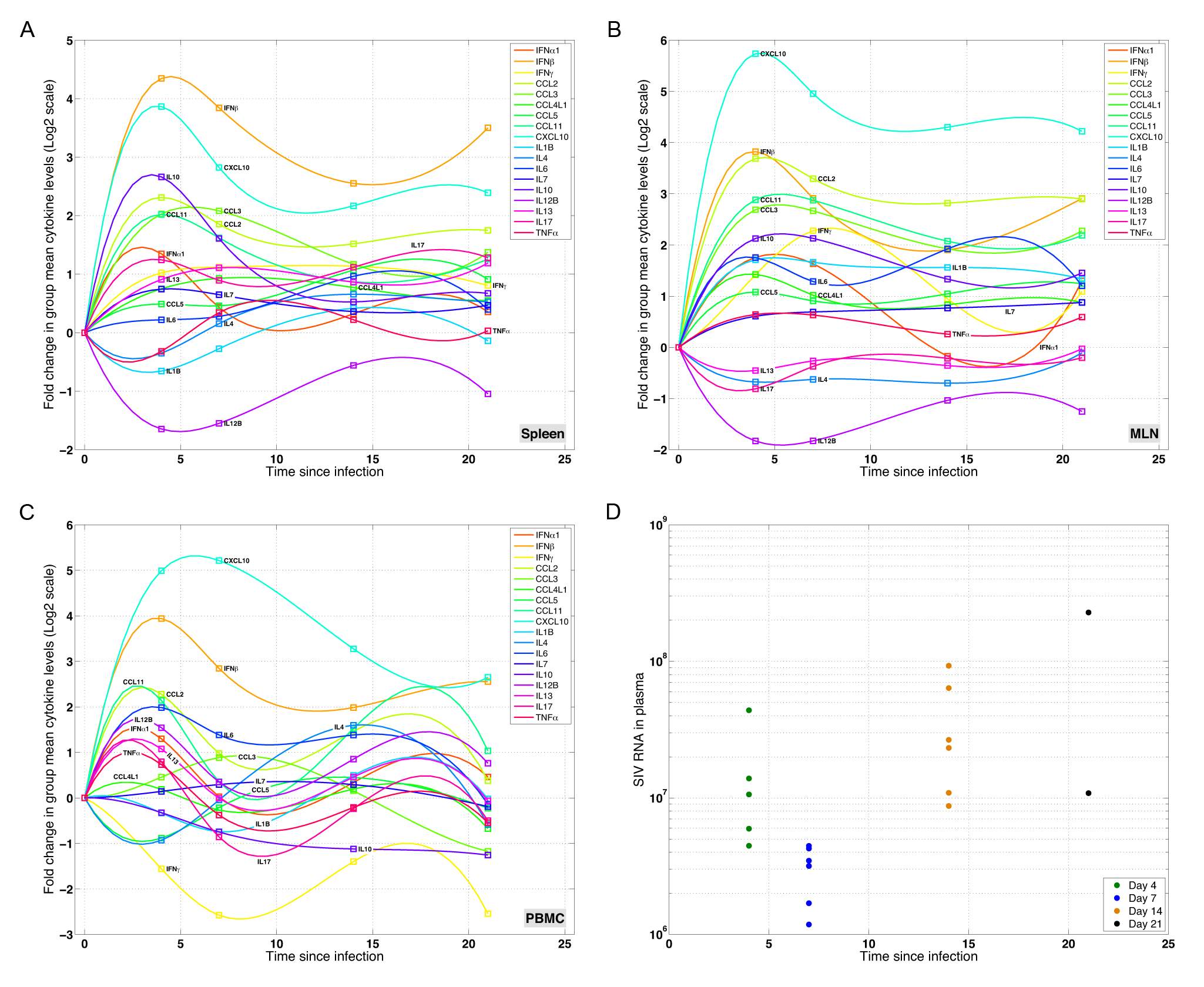
**
