# Supplementary material for: Multiplexed Component Analysis to Identify Genes Contributing to the Immune Response during Acute SIV Infection
Source: PLoS One. 2015 May 18;10(5):e0126843. doi: 10.1371/journal.pone.0126843 (PMC4436129; doi:10.1371/journal.pone.0126843)

# Figure S2. Percentage of variance captured by the top PCs

The total amount of variance captured by the top 4 PCs (dark colors) and the top 8 PCs (light colors) are shown for classification based on (A) time since infection and (B) SIV RNA in plasma in the spleen (green), MLN (blue), and PBMC (red) datasets. In all cases, at least 76% of the variance is captured by the top eight PCs.


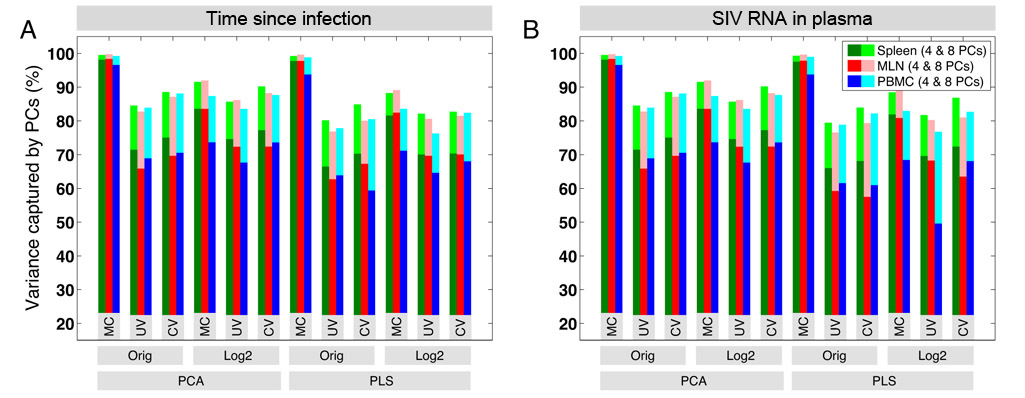

Supplement: S2 Information — (DOCX) [file pone.0126843.s008.docx]
