# Supplementary material for: Multiplexed Component Analysis to Identify Genes Contributing to the Immune Response during Acute SIV Infection
Source: PLoS One. 2015 May 18;10(5):e0126843. doi: 10.1371/journal.pone.0126843 (PMC4436129; doi:10.1371/journal.pone.0126843)

# Figures S3-S14. Score plots, loading plots and results of classification

Score plots provided by the *judges* are used to cluster observations into separate groups using a specific classification scheme (Figs. S3-S8). For each *judge*, we study 28 score plots generated by all the combinations of two of the top eight PCs. In each score plot, we perform centroid-based classification and leave-one-out cross validation (LOOCV) to obtain classification and LOOCV rates. The classification and LOOCV rates are indicative of the accuracy and the robustness of the classification on a given score plot, respectively. The PCs representing the highest accuracy and robustness are chosen as the top two classifier PCs for that *judge*. The classification and LOOCV rates (in parentheses) are written in the right bottom corner of each subplot. The top two classifier PCs are shown above the rates (Figs. S3-S8). After choosing the top two classifier PCs for each *judge*, loading plots are constructed by the chosen PCs (Figs. S9-S14).

**Figure S3. Score plots and results of classification based on time since infection in the spleen dataset**

**
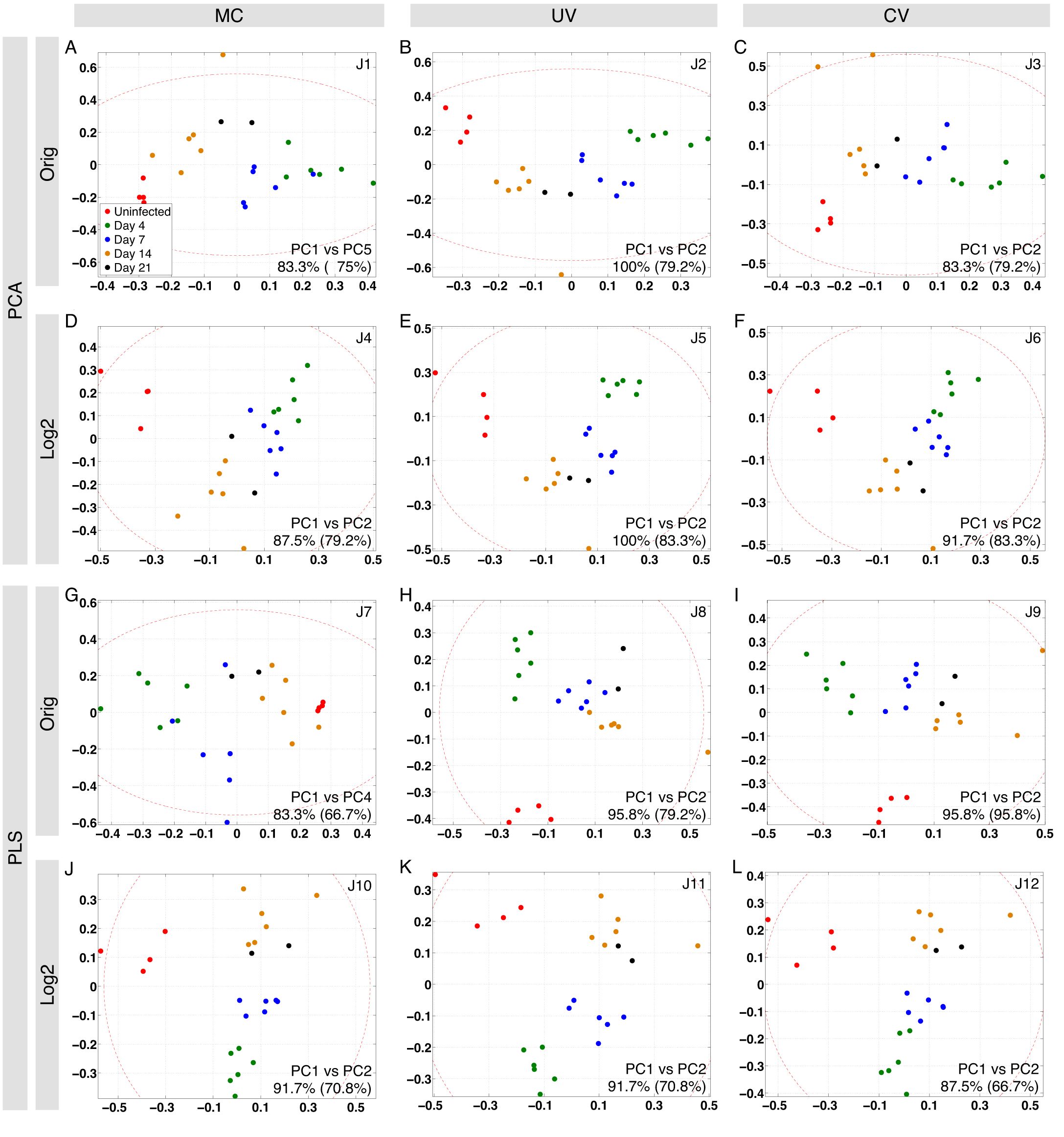
Figure S4. Score plots and results of classification based on time since infection in the MLN dataset**

**
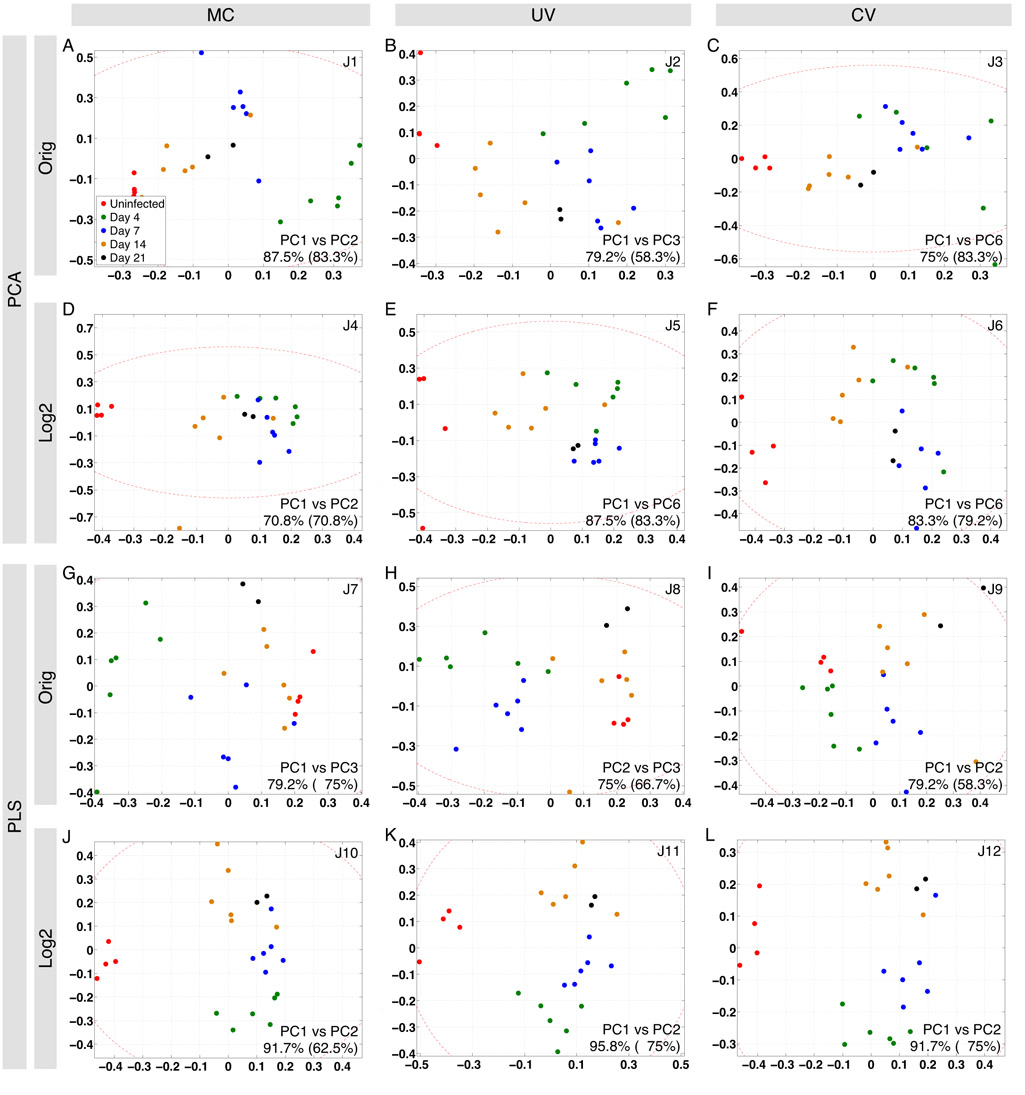
Figure S5. Score plots and results of classification based on time since infection in the PBMC dataset**


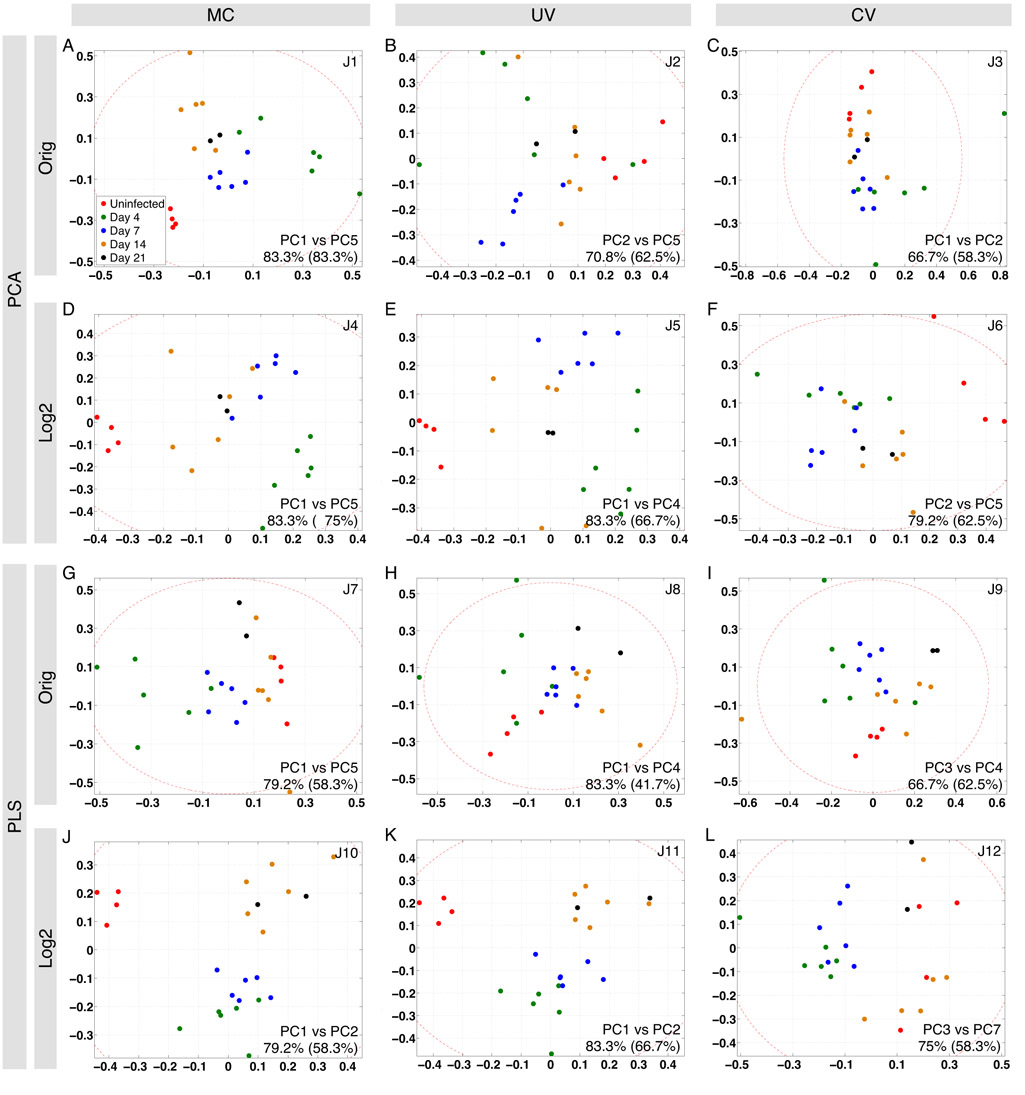


**Figure S6. Score plots and results of classification based on SIV RNA in plasma in the spleen dataset**


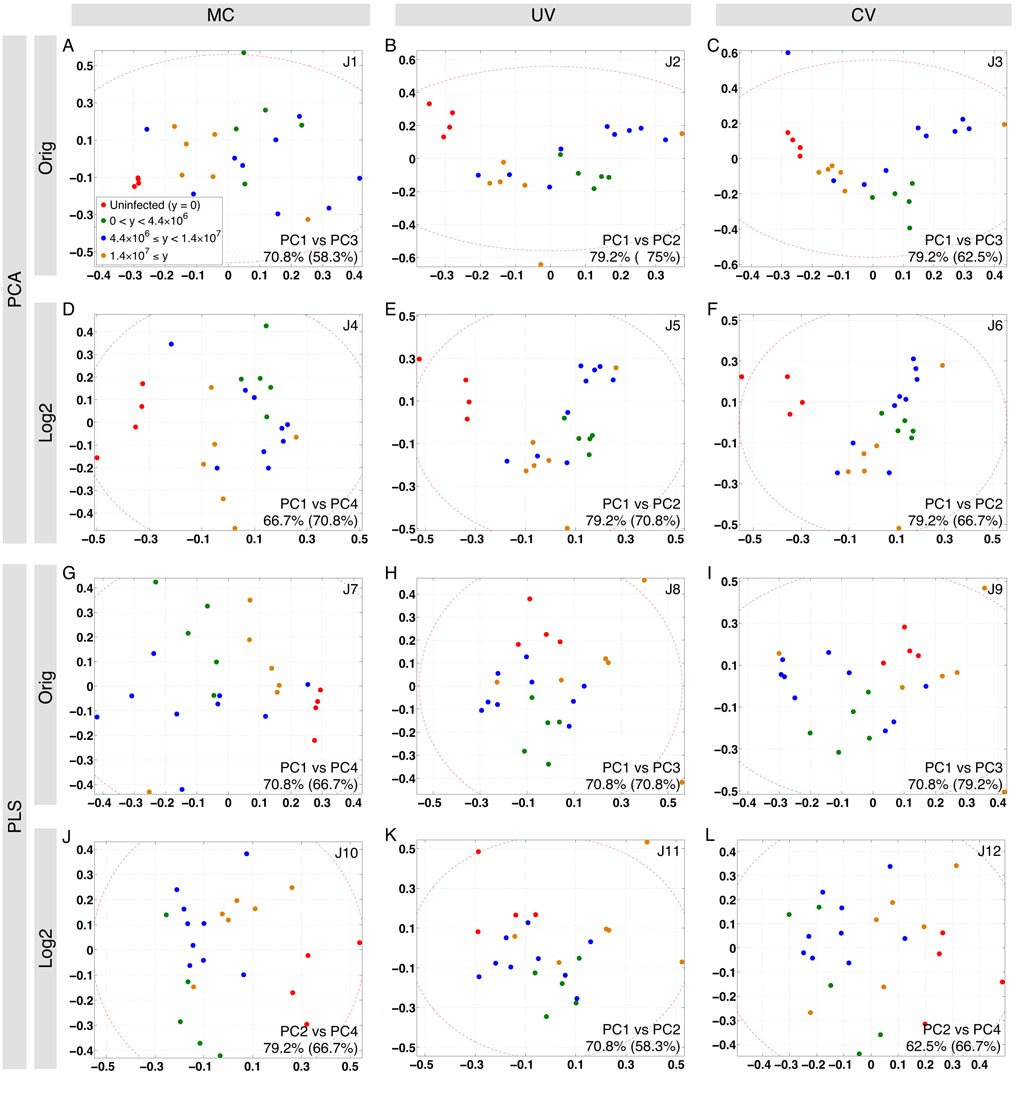


**Figure S7. Score plots and results of classification based on SIV RNA in plasma in the MLN dataset**


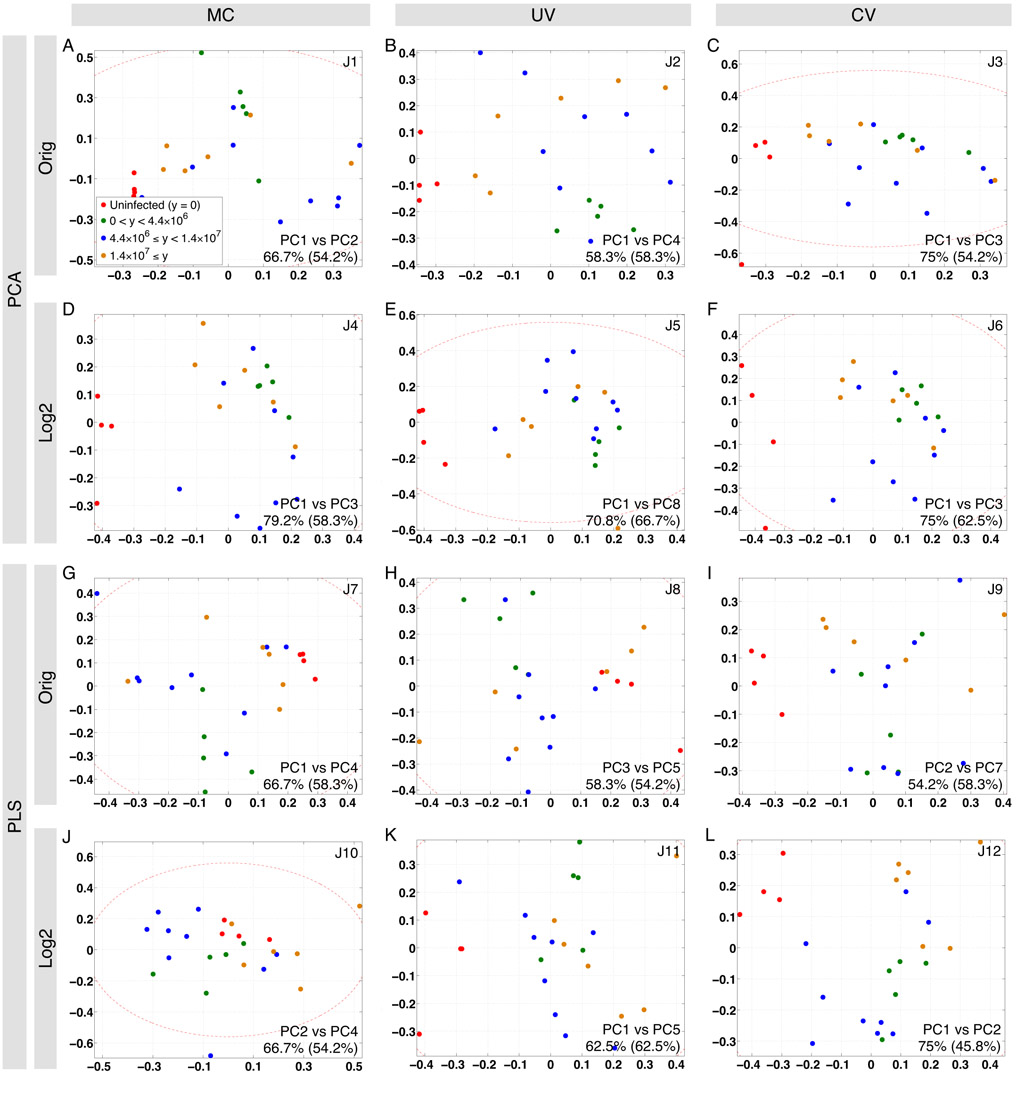


**Figure S8. Score plots and results of classification based on SIV RNA in plasma in the PBMC dataset**


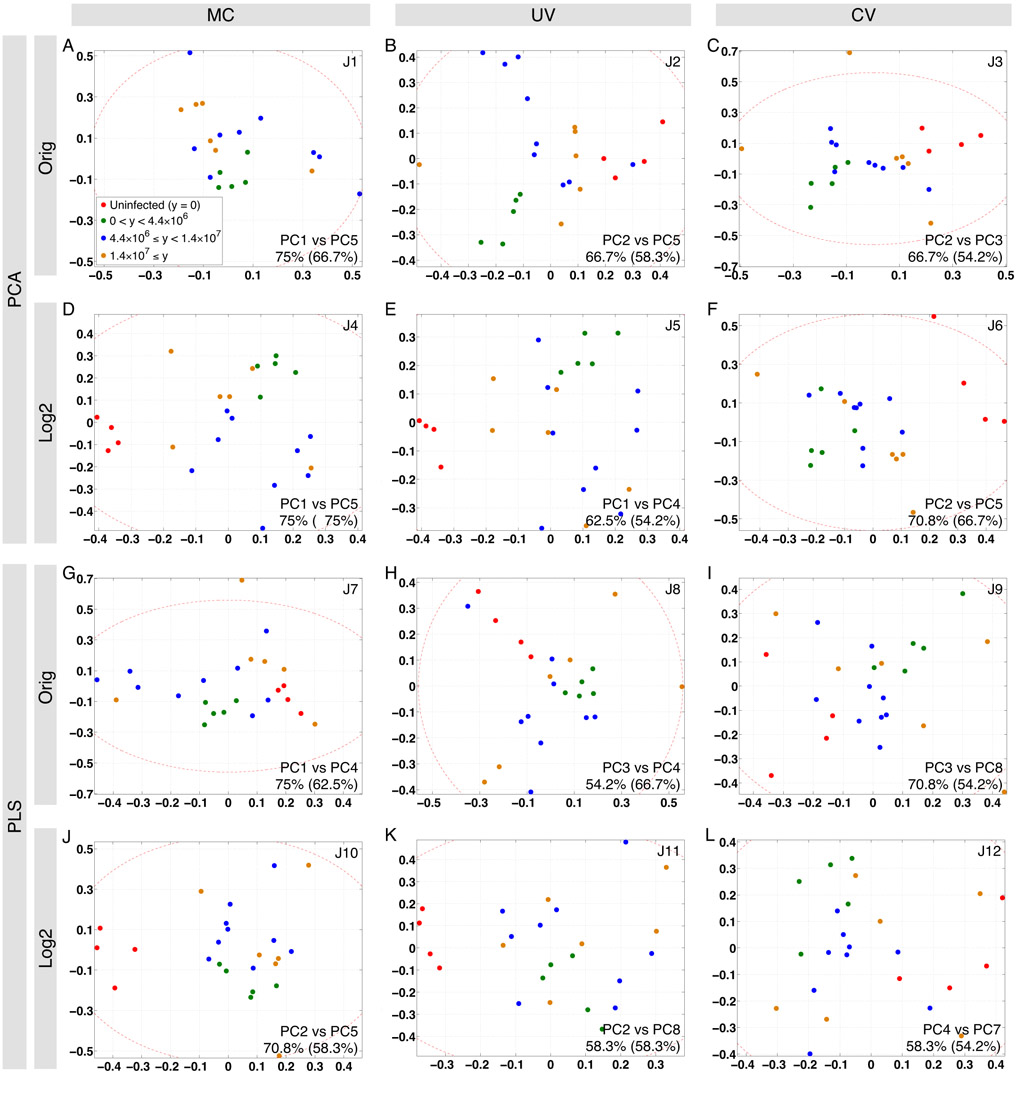


**Figure S9.** **Loading plots constructed by the top two classifier PCs chosen by classification based on time since infection in the spleen dataset**


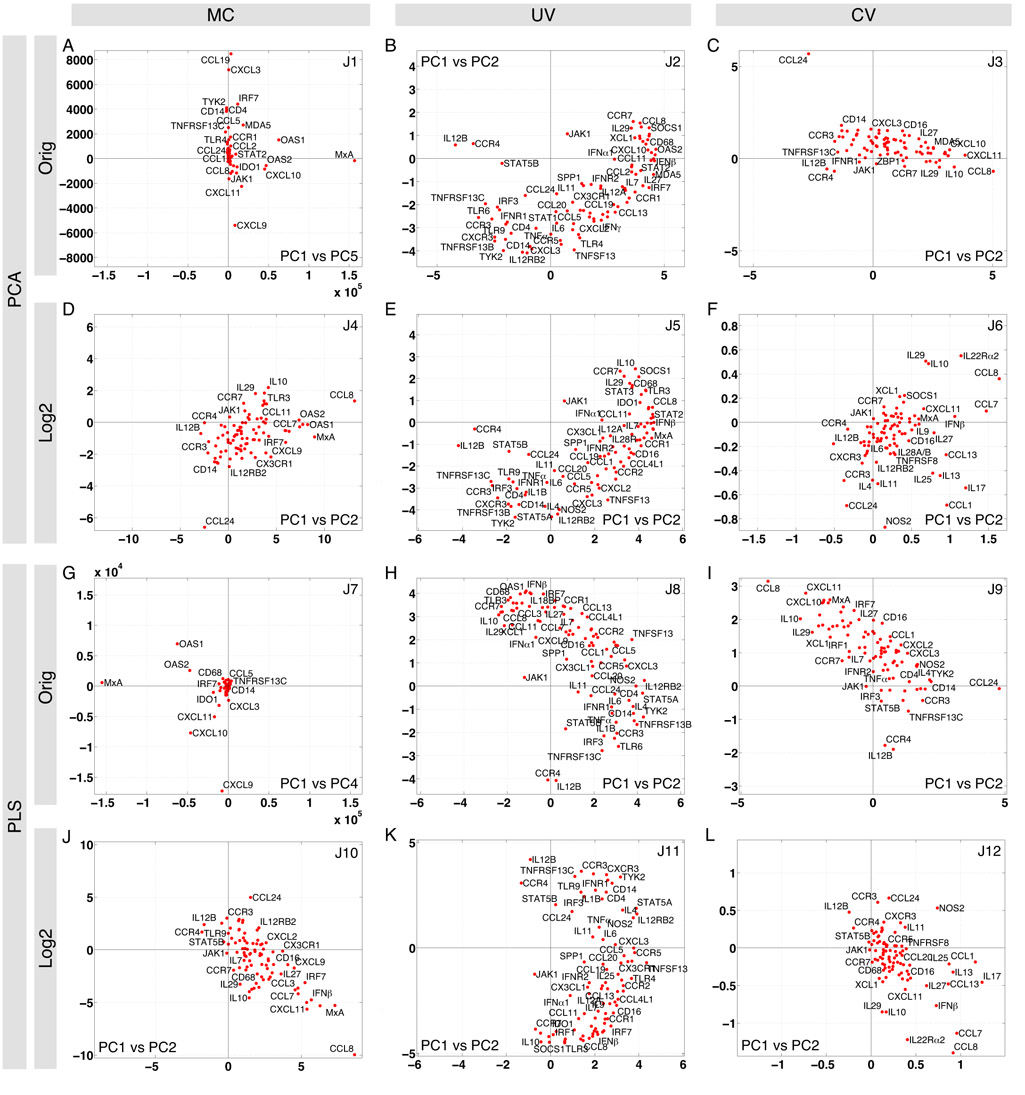
**Figure S10.** **Loading plots constructed by the top two classifier PCs chosen by classification based on time since infection in the MLN dataset**


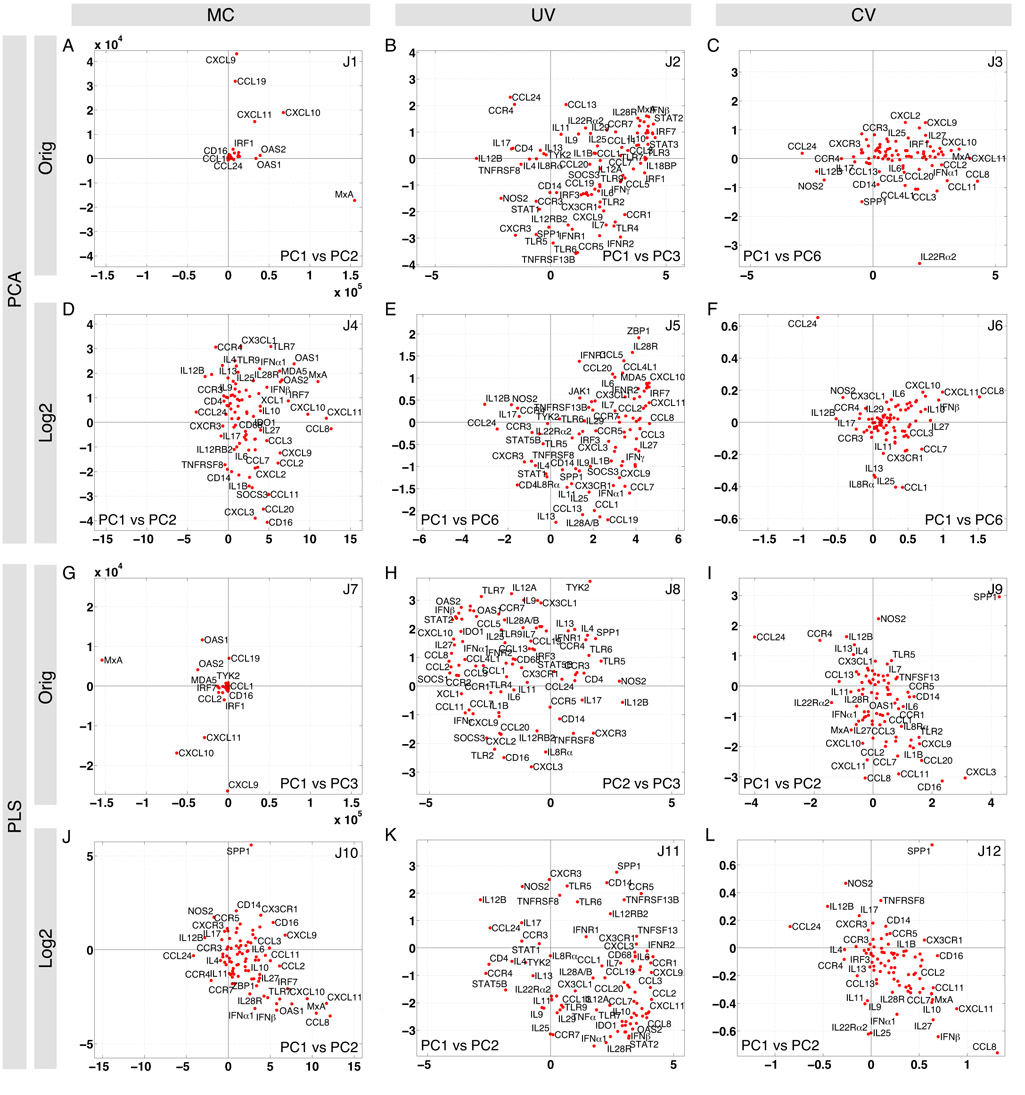
**Figure S11.** **Loading plots constructed by the top two classifier PCs chosen by classification based on time since infection in the PBMC dataset**


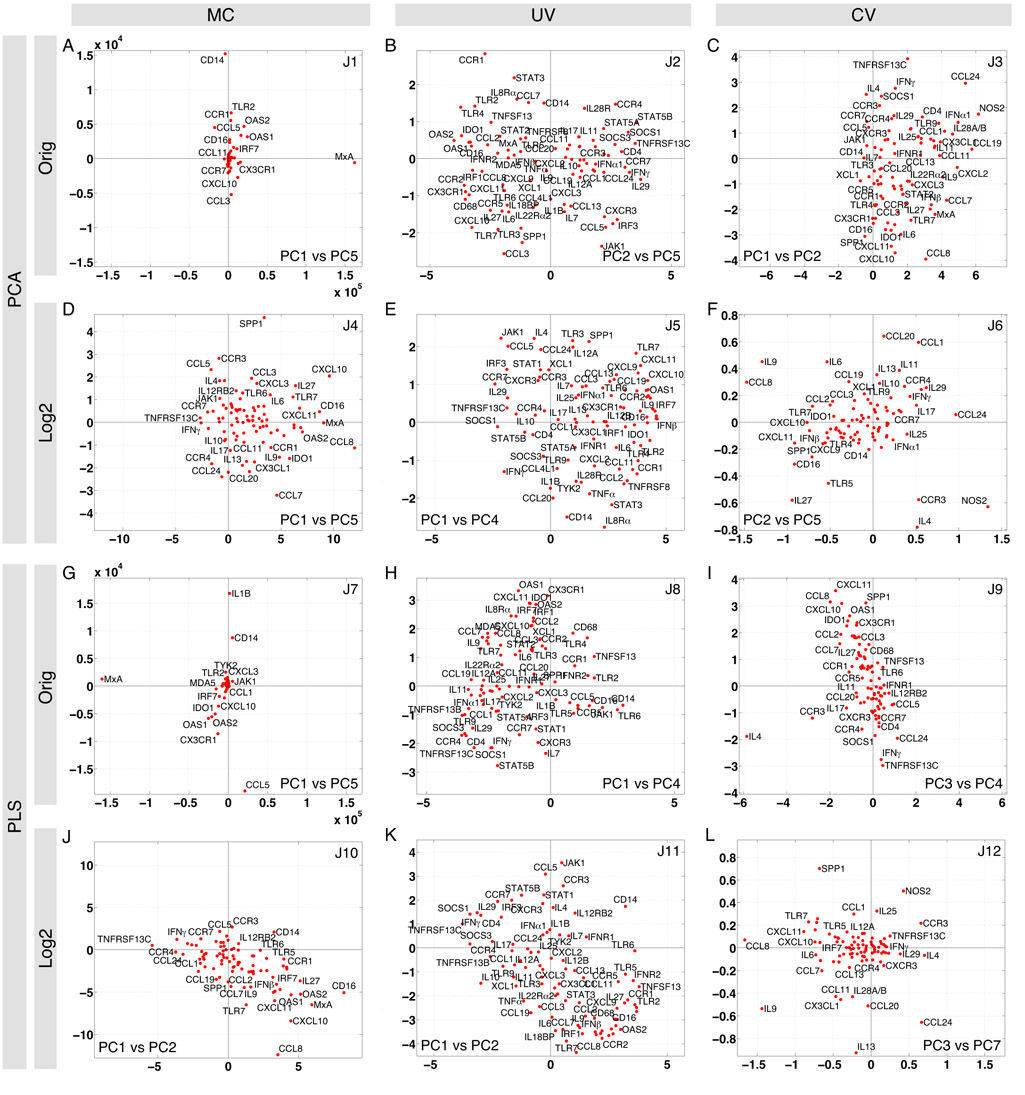
**Figure S12.** **Loading plots constructed by the top two classifier PCs chosen by classification based on SIV RNA in plasma in the spleen dataset**


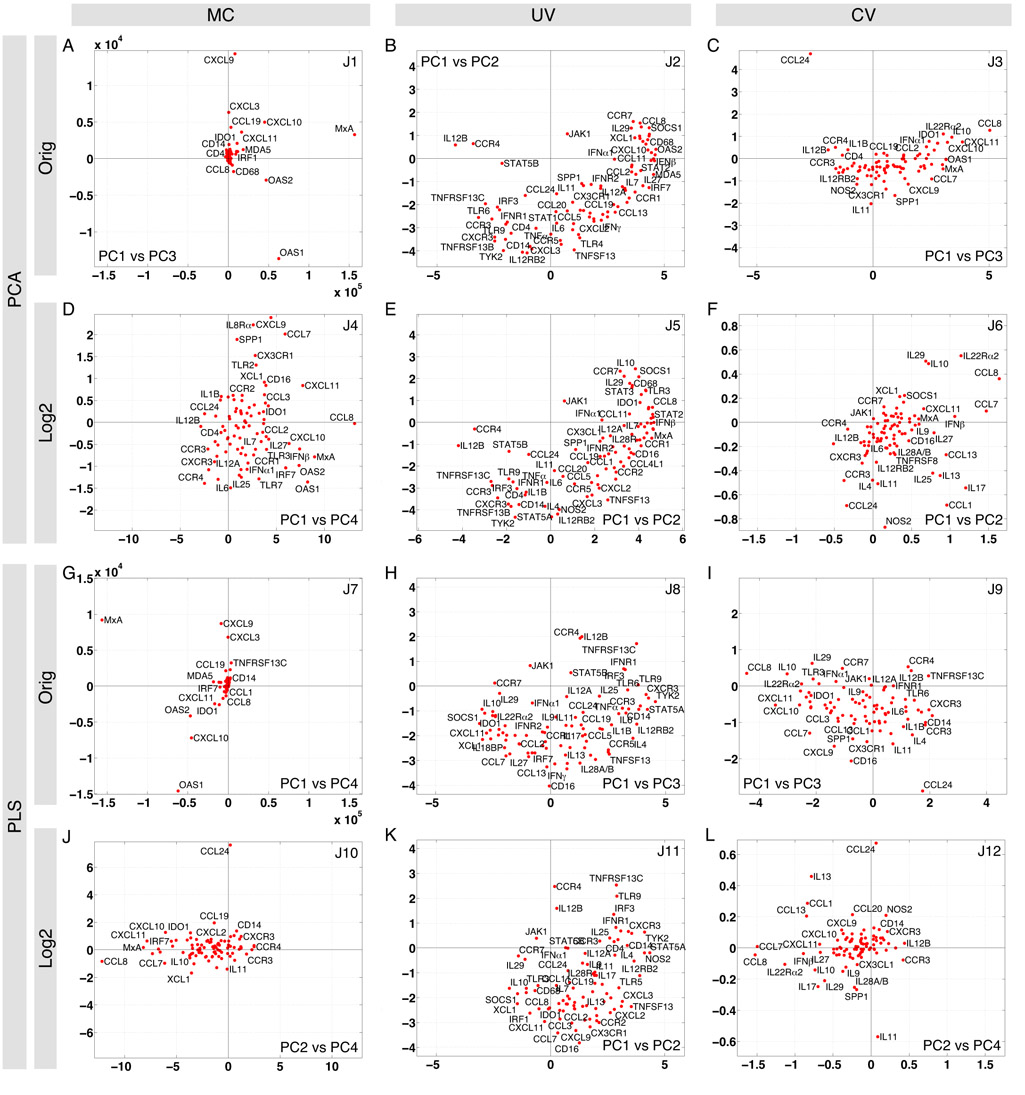
**Figure S13.** **Loading plots constructed by the top two classifier PCs chosen by classification based on SIV RNA in plasma in the MLN dataset**


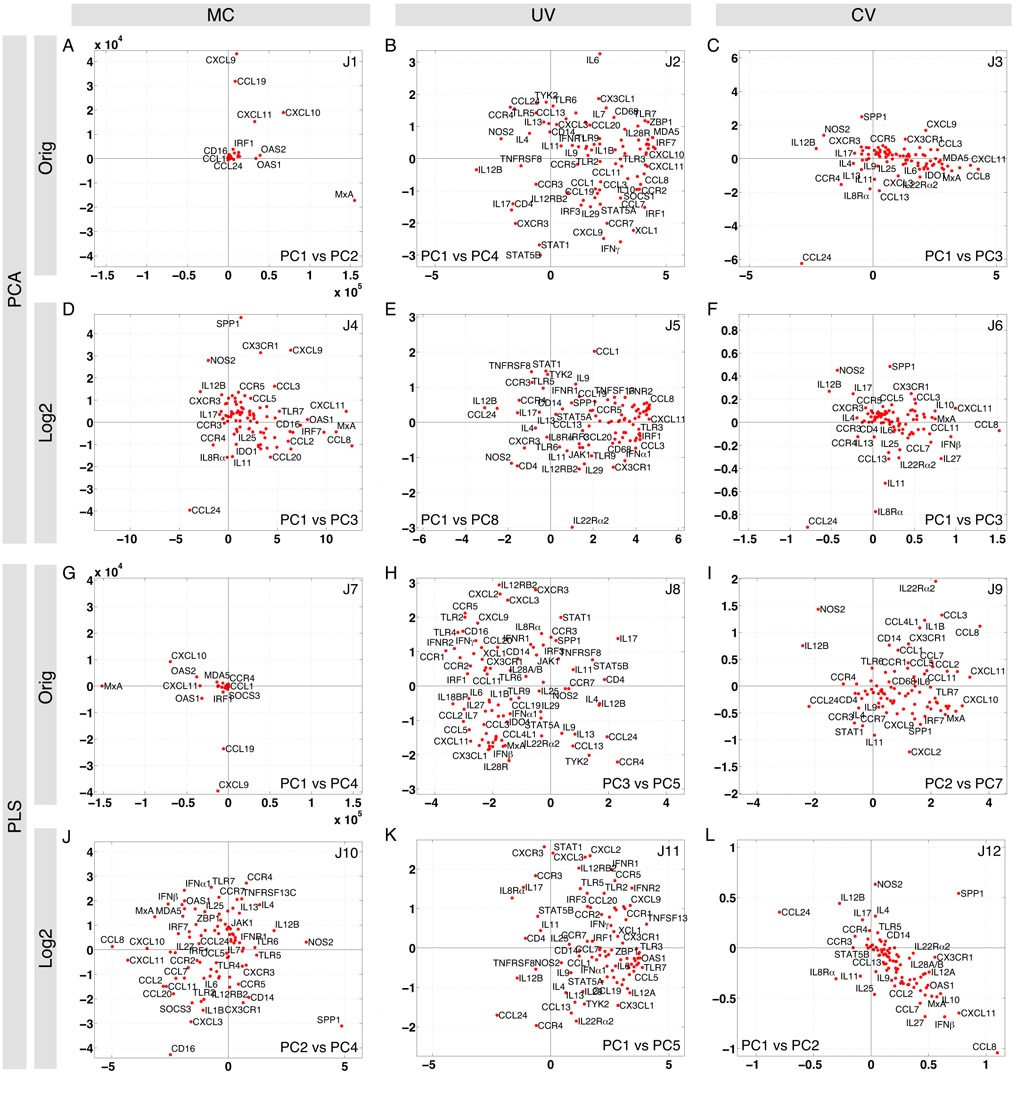
**Figure S14.** **Loading plots constructed by the top two classifier PCs chosen by classification based on SIV RNA in plasma in the PBMC dataset**


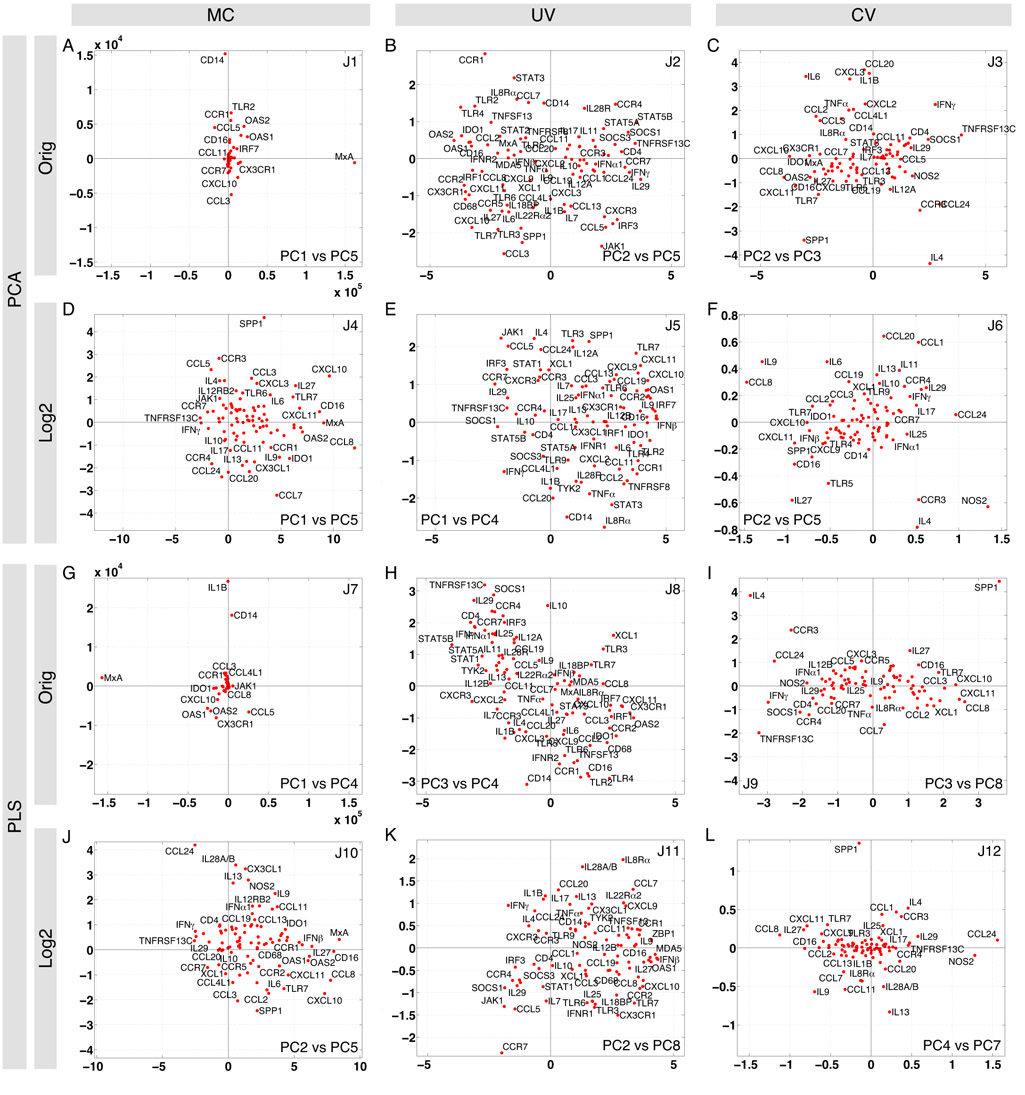

Supplement: S3 Information — (DOCX) [file pone.0126843.s009.docx]
