# Supplementary material for: Multiplexed Component Analysis to Identify Genes Contributing to the Immune Response during Acute SIV Infection
Source: PLoS One. 2015 May 18;10(5):e0126843. doi: 10.1371/journal.pone.0126843 (PMC4436129; doi:10.1371/journal.pone.0126843)

# Figure S15. Gene rankings across datasets for classification based on SIV RNA in plasma. (Fig. S15D is the same as Fig. 5E)


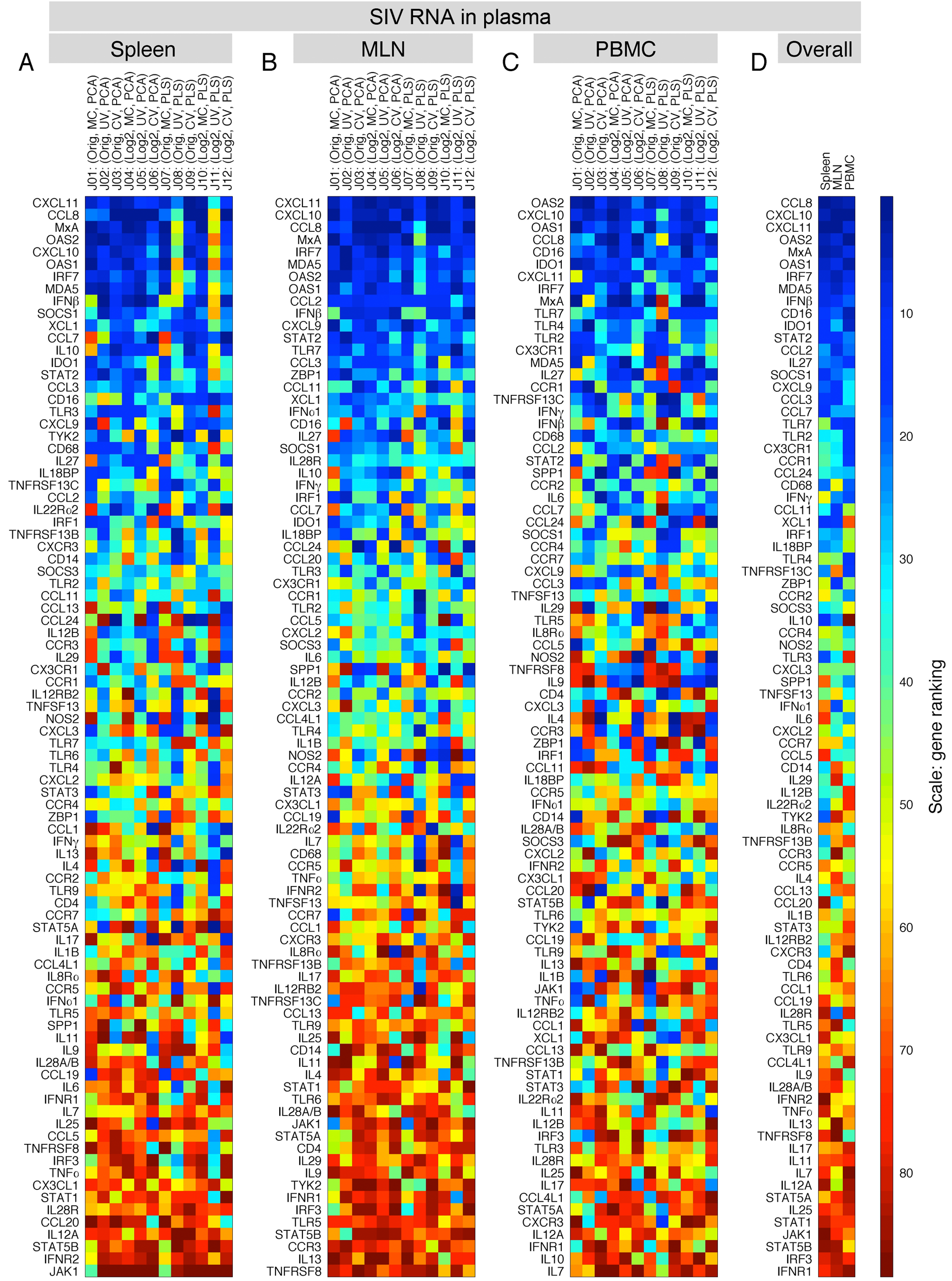

Supplement: S4 Information — (DOCX) [file pone.0126843.s010.docx]
