# Supplementary figures and images for: Multiplexed Component Analysis to Identify Genes Contributing to the Immune Response during Acute SIV Infection
Source: PLoS One. 2015 May 18;10(5):e0126843. doi: 10.1371/journal.pone.0126843 (PMC4436129; doi:10.1371/journal.pone.0126843)

# Figure S16. Gene rankings by the Pearson correlation method


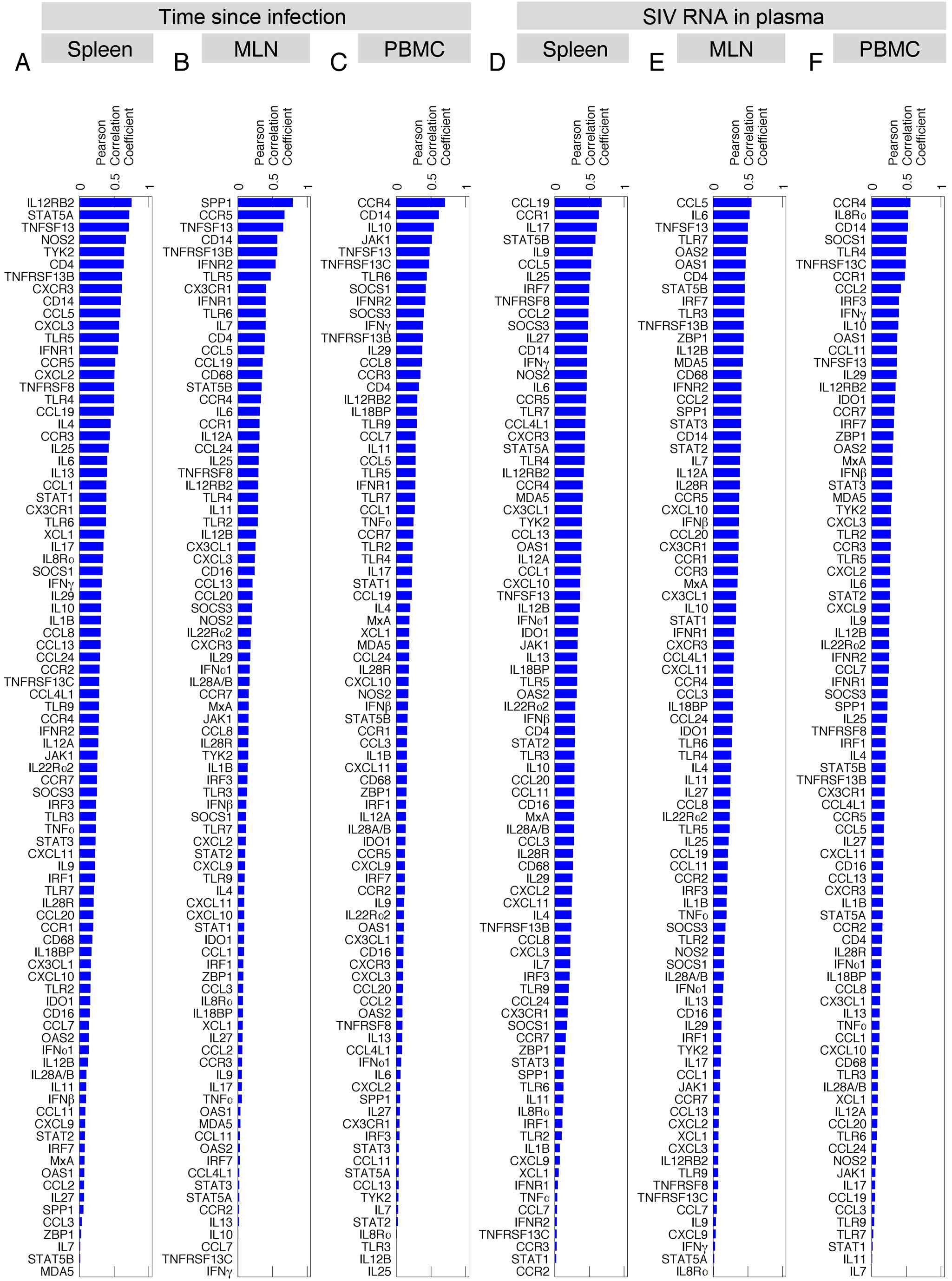

Supplement: S5 Information — (DOCX) [file pone.0126843.s011.docx]

# Figure S17. Gene rankings by the Spearman correlation method


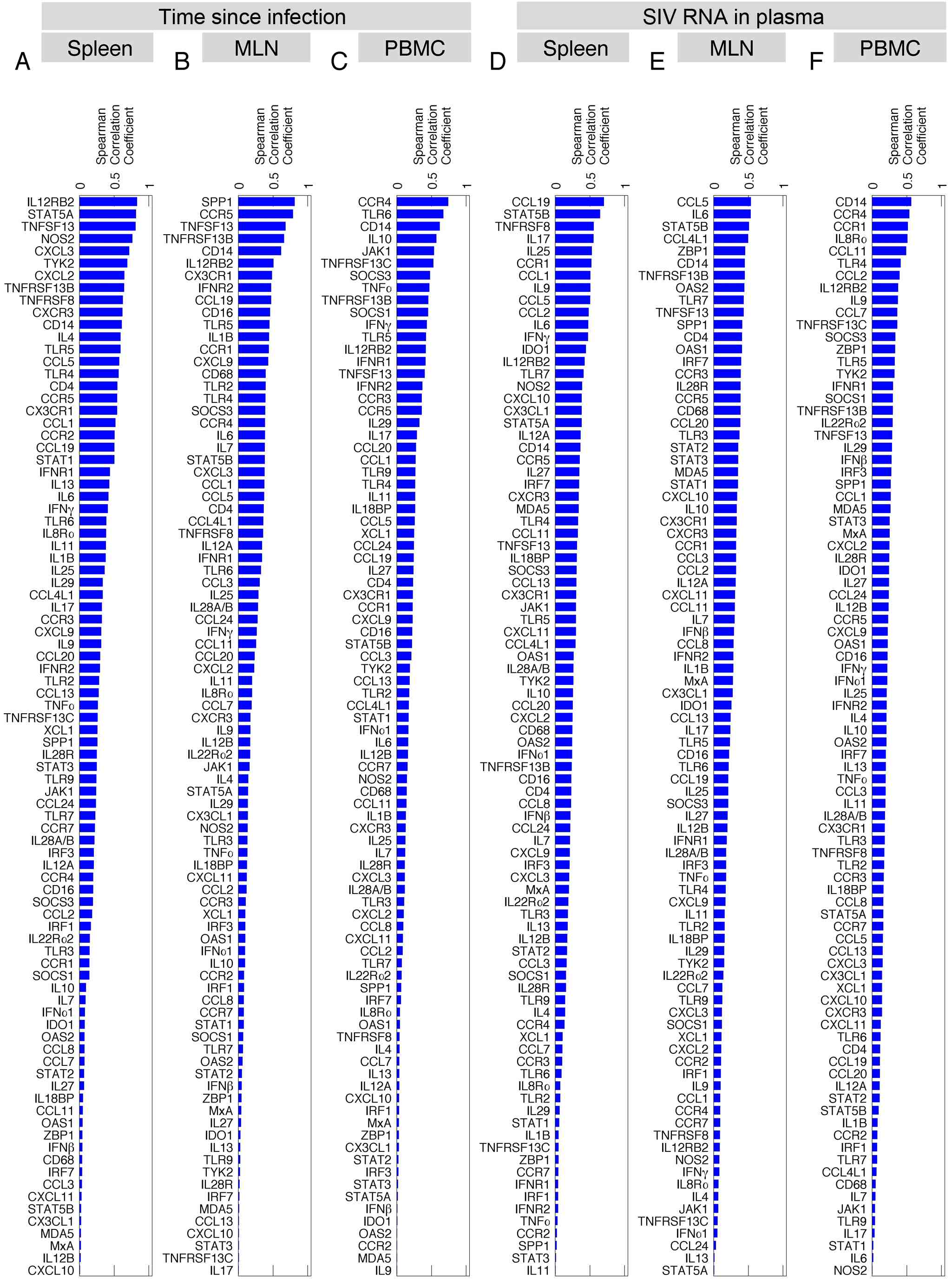

Supplement: S6 Information — (DOCX) [file pone.0126843.s012.docx]

# Figure S18. Gene rankings by the one-way ANOVA method


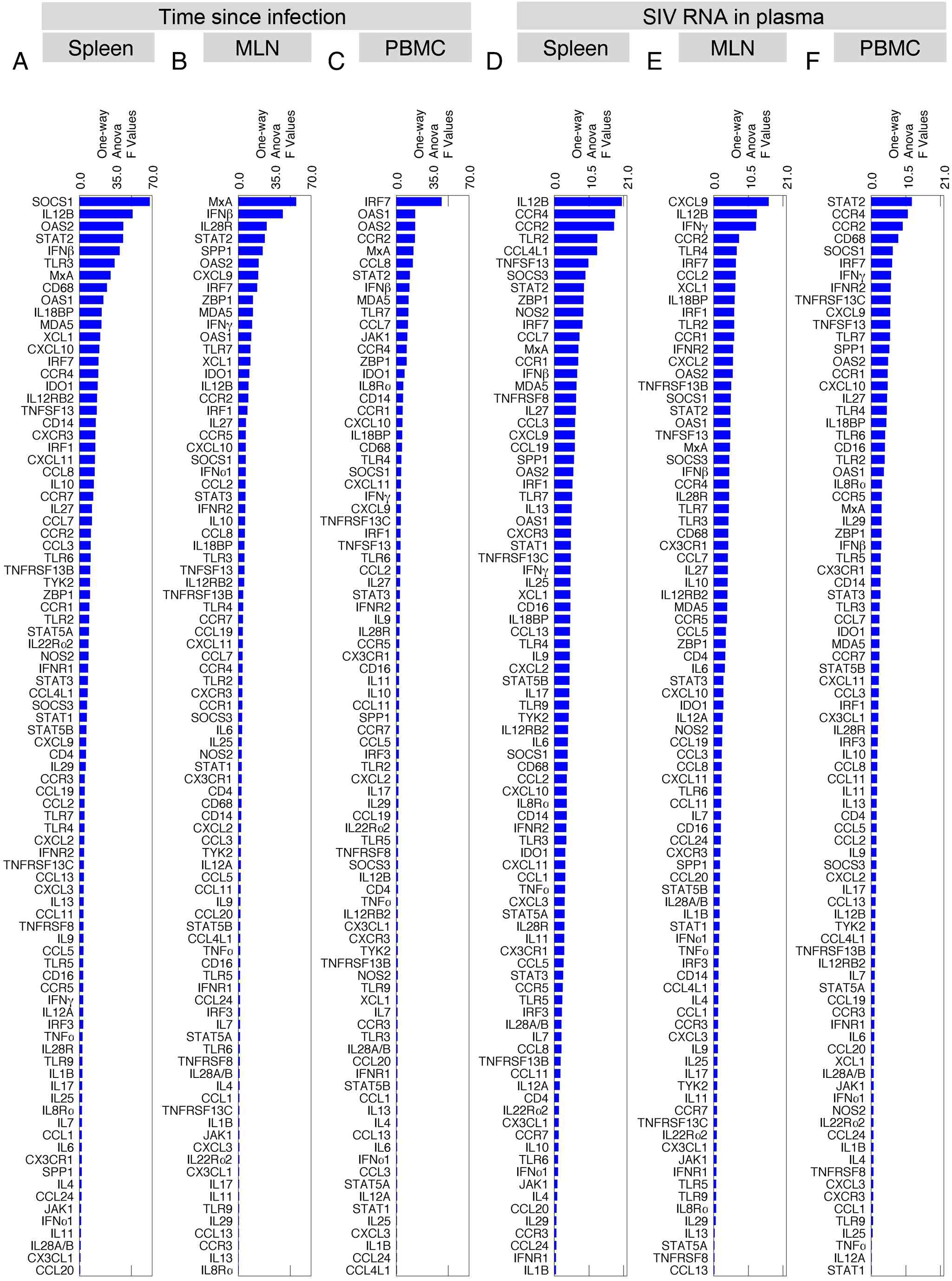

Supplement: S7 Information — (DOCX) [file pone.0126843.s013.docx]

# Figure S19. Gene rankings by the significance analysis of microarrays (SAM) method


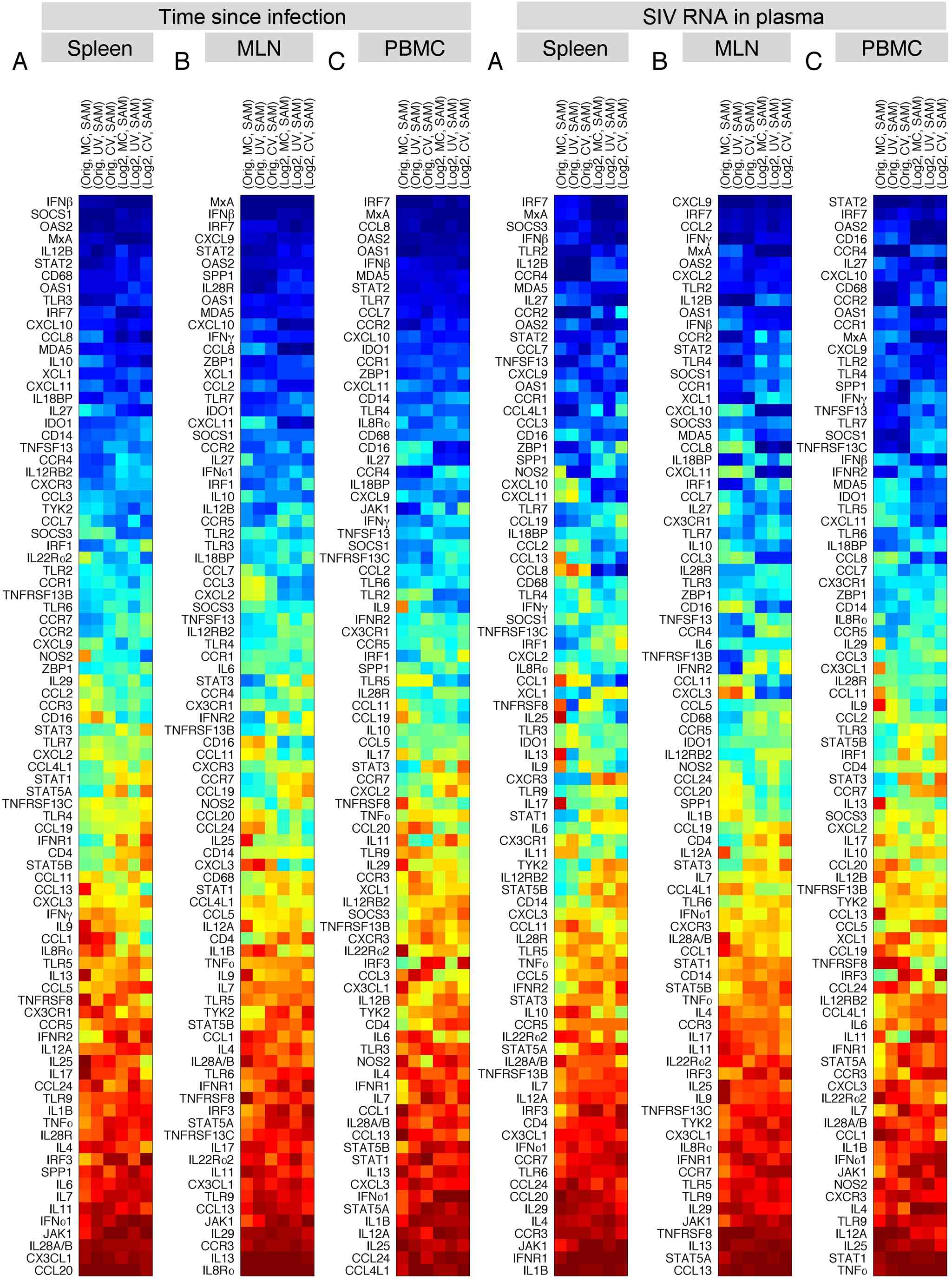

Supplement: S8 Information — (DOCX) [file pone.0126843.s014.docx]

# Figure S46. The loading plots of average correlation coefficient matrices shown in Fig. 8

**
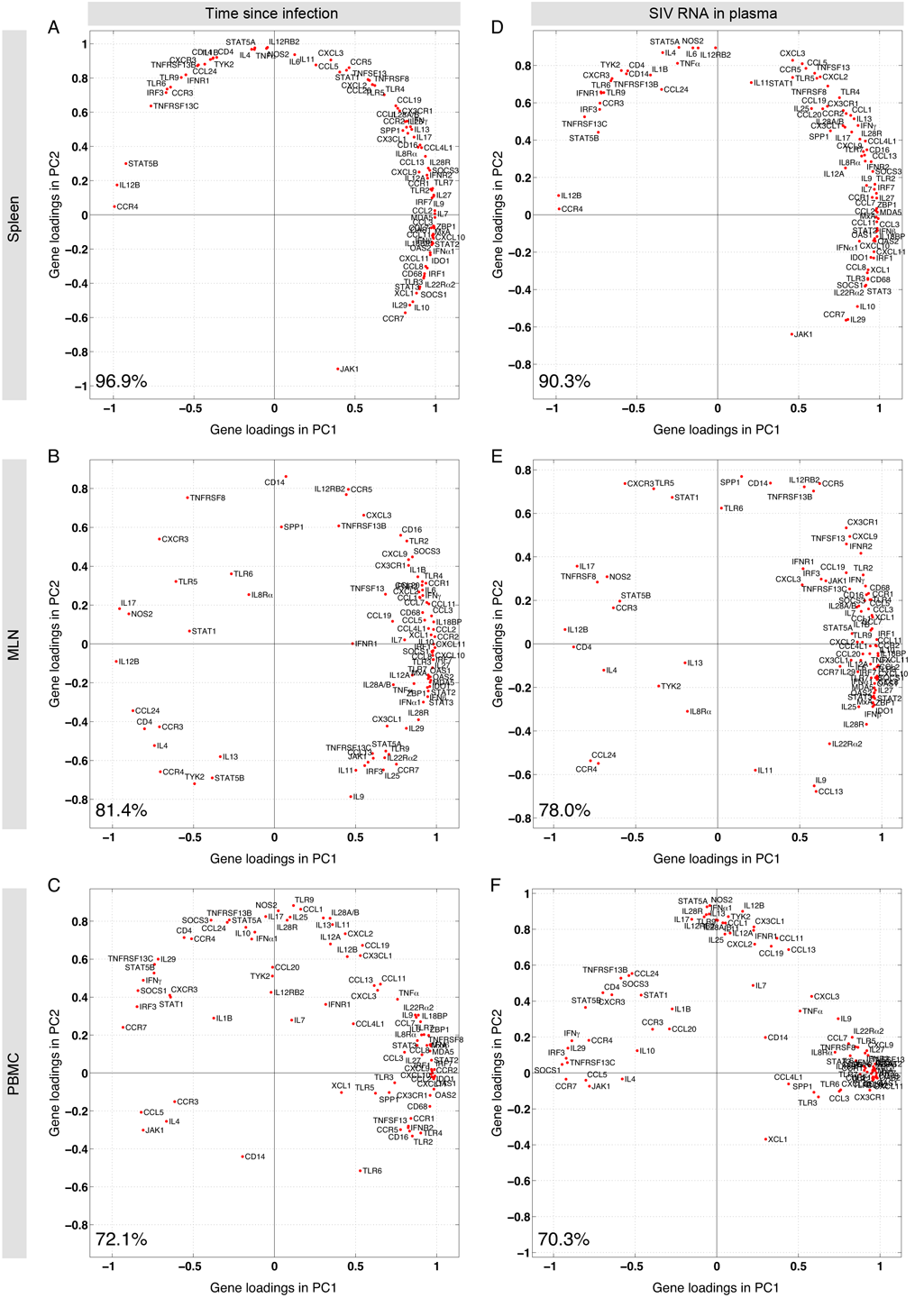
**

Supplement: S16 Information — (DOCX) [file pone.0126843.s022.docx]

# Figure S47. The correlation coefficient matrices obtained from the loading plots in Fig. S46

#
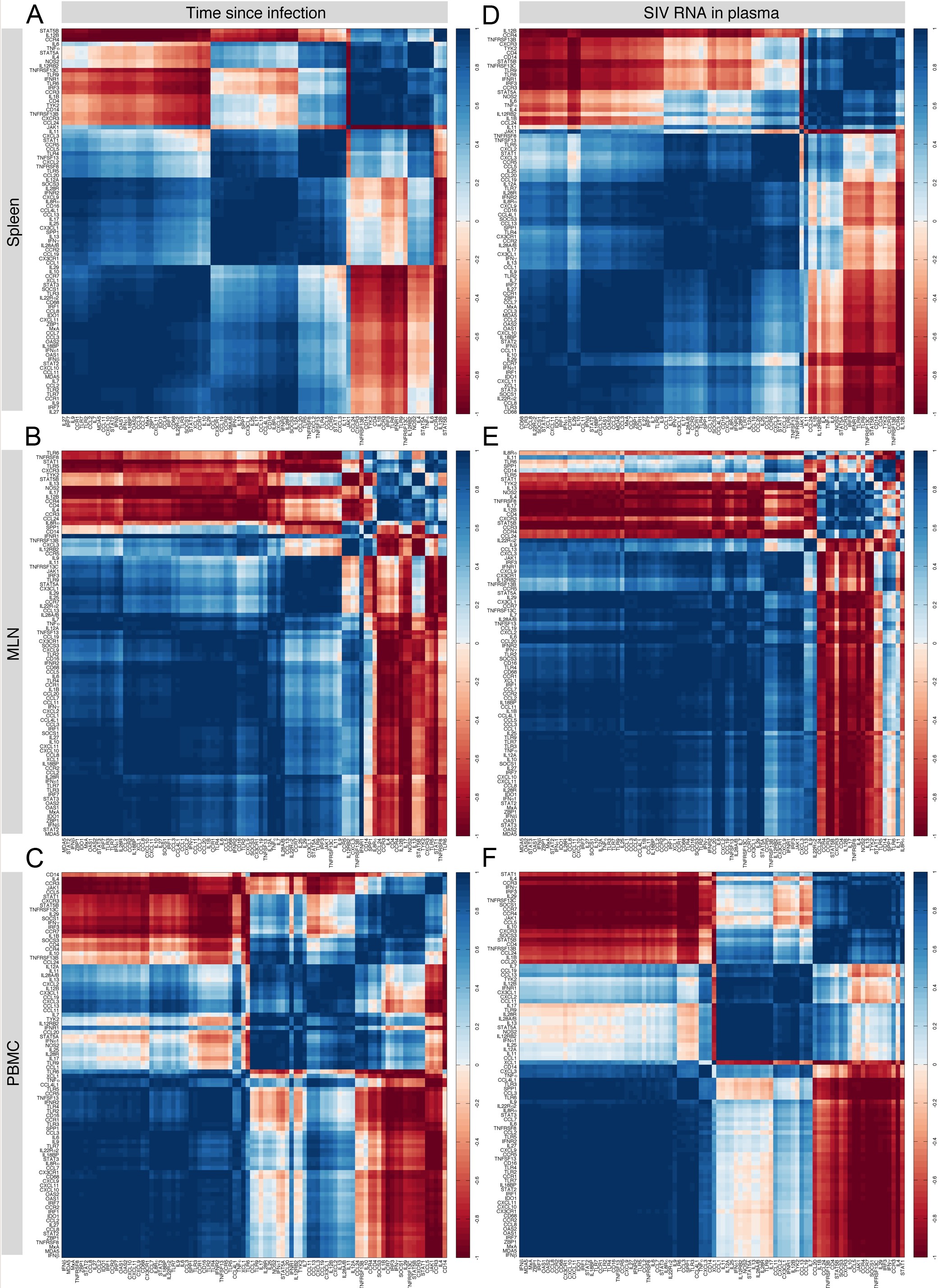

Supplement: S17 Information — (DOCX) [file pone.0126843.s023.docx]

# Figure S54. Schematic of algorithm to plot Fig. 9 (polar plots)


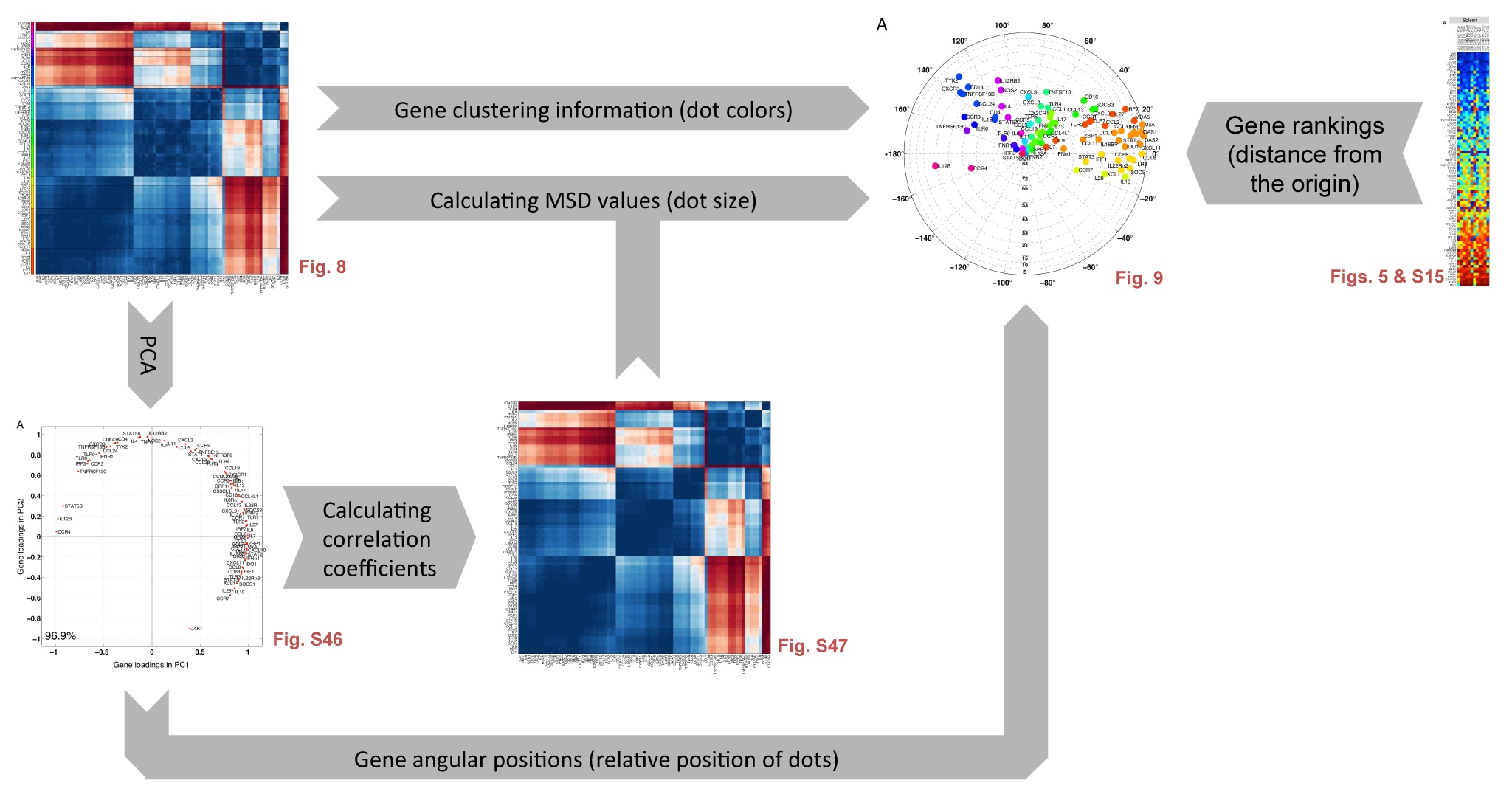

Supplement: S19 Information — (DOCX) [file pone.0126843.s025.docx]
