# Supplementary material for: Multiplexed Component Analysis to Identify Genes Contributing to the Immune Response during Acute SIV Infection
Source: PLoS One. 2015 May 18;10(5):e0126843. doi: 10.1371/journal.pone.0126843 (PMC4436129; doi:10.1371/journal.pone.0126843)

# Figure S20. Classification results and evaluation of ranking similarity for the Pearson correlation, Spearman correlation, ANOVA, SAM and MCA methods

(A and C) For each method, we selected the top five genes in each dataset and built decision trees to classify the observations using the selected genes. (B and D) We used Spearman’s rank correlation coefficients to measure the degree of similarity between the rankings of the MCA and other methods.

#
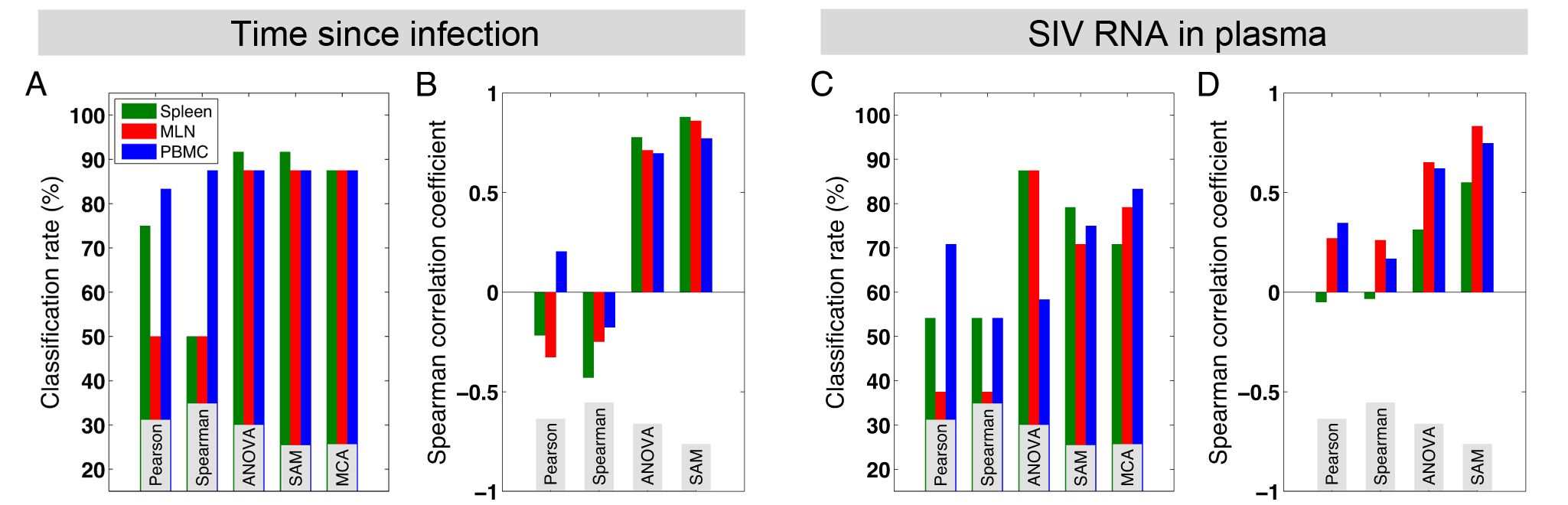

Supplement: S9 Information — (DOCX) [file pone.0126843.s015.docx]
