# Supplementary material for: Multiplexed Component Analysis to Identify Genes Contributing to the Immune Response during Acute SIV Infection
Source: PLoS One. 2015 May 18;10(5):e0126843. doi: 10.1371/journal.pone.0126843 (PMC4436129; doi:10.1371/journal.pone.0126843)

# Figure S21. Classification results for the individual *judges*

For each *judge*, we selected the top five genes in each dataset and built decision trees to classify the observations using the selected genes for (A) time since infection and (B) SIV RNA in plasma. The last column, labeled MCA, shows the results when we performed classification using the top five average-ranked genes by all the *judges*.

#
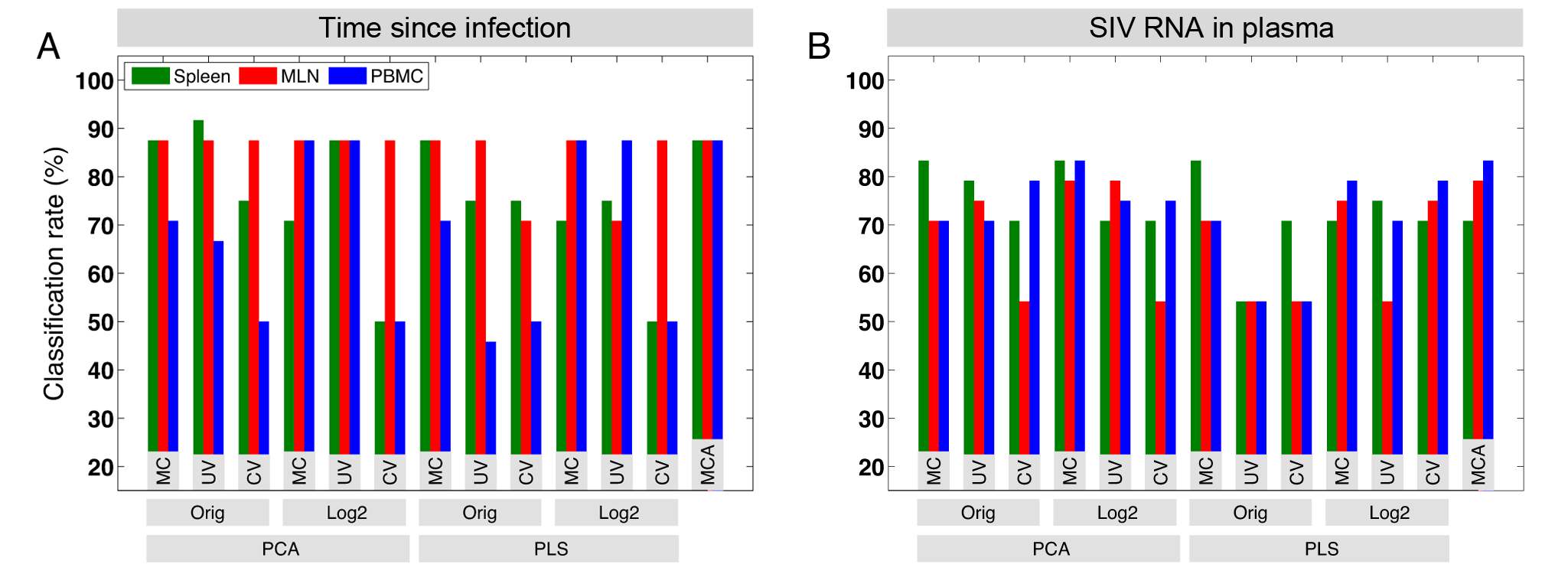

Supplement: S10 Information — (DOCX) [file pone.0126843.s016.docx]
