# Supplementary material for: Multiplexed Component Analysis to Identify Genes Contributing to the Immune Response during Acute SIV Infection
Source: PLoS One. 2015 May 18;10(5):e0126843. doi: 10.1371/journal.pone.0126843 (PMC4436129; doi:10.1371/journal.pone.0126843)

# Figure S22. Classification results for the *judges* with log2-transformation

For *judges with* log2-transformation, we selected the top five average-ranked genes in each dataset and built decision trees to classify the log-transformed observations using the selected genes for (A) time since infection and (B) SIV RNA in plasma. The second column, labeled MCA, shows the results when we performed classification using the top five average-ranked genes by all the *judges*.


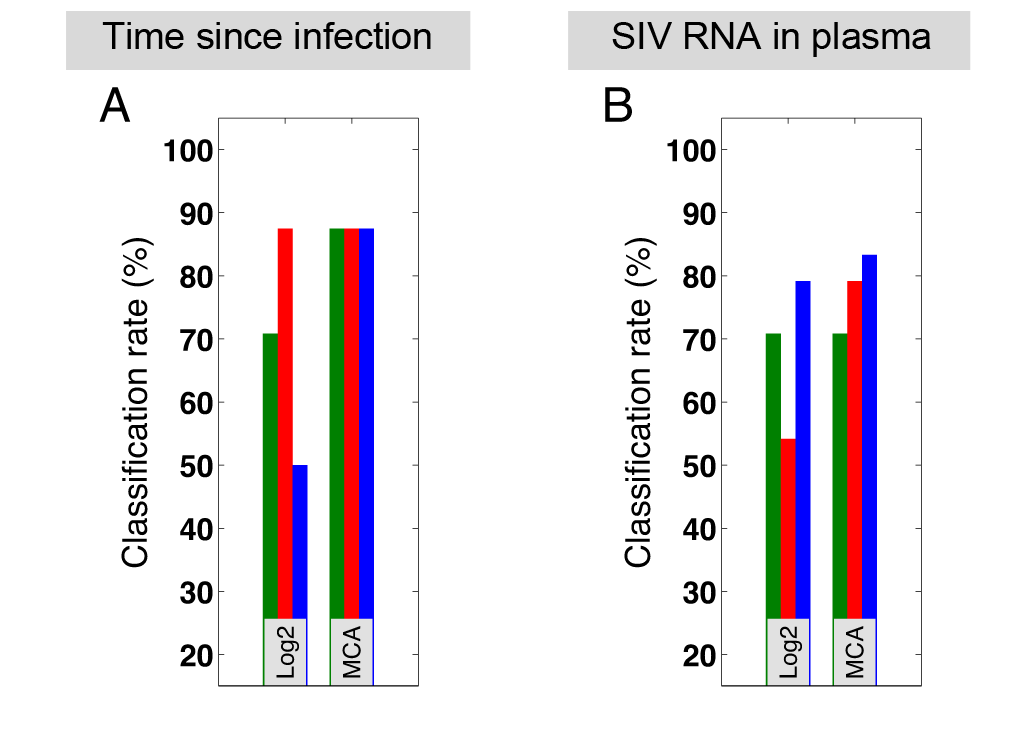

Supplement: S11 Information — (DOCX) [file pone.0126843.s017.docx]
