# Supplementary material for: Multiplexed Component Analysis to Identify Genes Contributing to the Immune Response during Acute SIV Infection
Source: PLoS One. 2015 May 18;10(5):e0126843. doi: 10.1371/journal.pone.0126843 (PMC4436129; doi:10.1371/journal.pone.0126843)

# Figures S23-S28. Consensus plots showing the degree of agreement between *judges* on the contribution of genes in all datasets and for both classification schemes

To measure the degree of agreement between *judges* on the contribution a specific gene, we calculate the range and the standard deviation of the 12 ranks for that gene. The *judges* have a high degree of agreement on the contribution of a gene when both the standard deviation and the range take low values. One should note that for a given value of the standard deviation, there is more disagreement on the contribution of a gene with a higher value for the range, i.e. as we horizontally move from left to right, the degree of disagreement from *judges* increases. Also, for a given value of the range, there is more disagreement on the contribution of a gene with a higher value of standard deviation, i.e. as we vertically move from the bottom to the top, genes experience a higher degree of disagreement from *judges*. For example in Fig. S24 (time since infection - MLN), MxA experiences a higher degree of disagreement compared to CCL8 (on the same horizontal line) and there is less consensus between *judges* on STAT1 compared to CCL3 (on the same vertical line). The color of each dot (gene) represents its overall rank in the dataset.

**Figure S23. The consensus plot showing the standard deviation and the range of the 12 ranks given to each gene for classification based on time since infection in the spleen dataset**

**
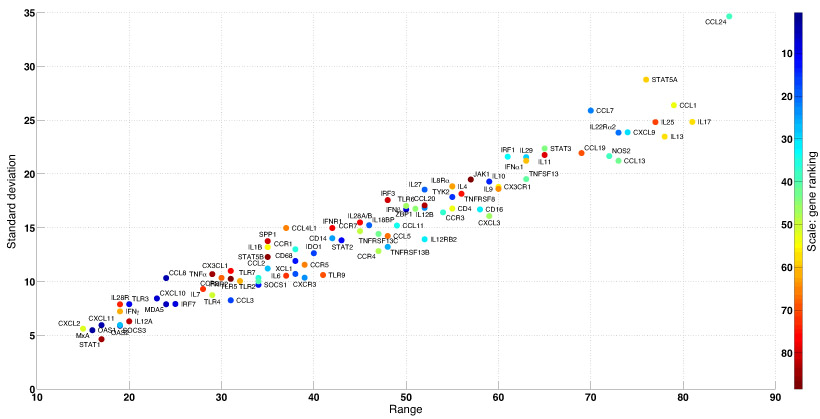
Figure S24. The consensus plot showing the standard deviation and the range of the 12 ranks given to each gene for classification based on time since infection in the MLN dataset**

**
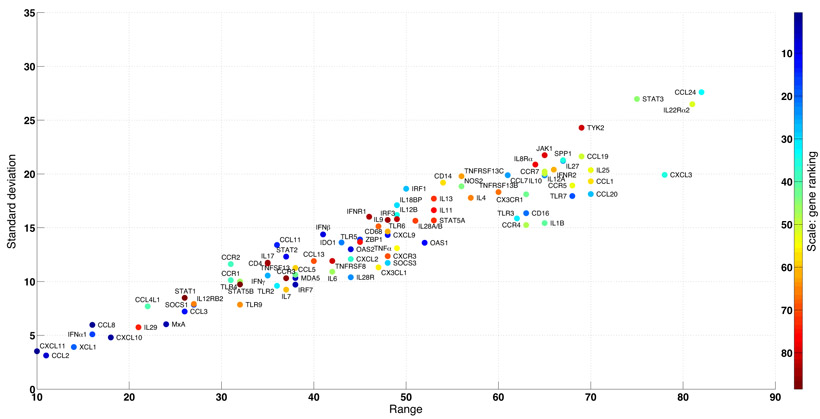
Figure S25. The consensus plot showing the standard deviation and the range of the 12 ranks given to each gene for classification based on time since infection in the PBMC dataset**

**
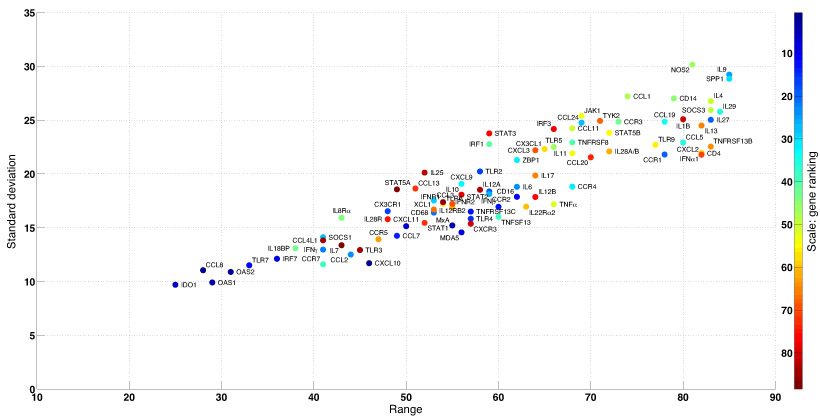
Figure S26. The consensus plot showing the standard deviation and the range of the 12 ranks given to each gene for classification based on SIV RNA in plasma in the spleen dataset**

**
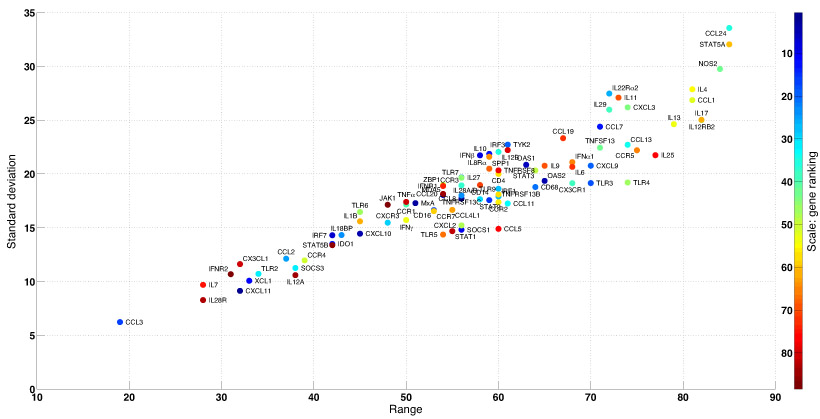
Figure S27. The consensus plot showing the standard deviation and the range of the 12 ranks given to each gene for classification based on SIV RNA in plasma in the MLN dataset**

**
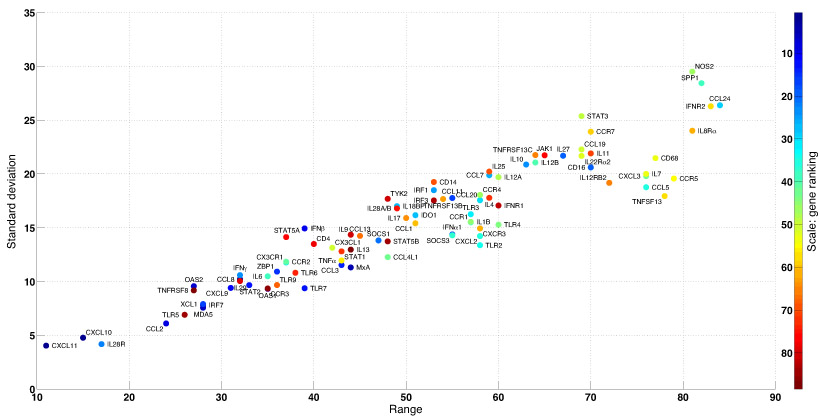
**

**Figure S28. The consensus plot showing the standard deviation and the range of the 12 ranks given to each gene for classification based on SIV RNA in plasma in the PBMC dataset**


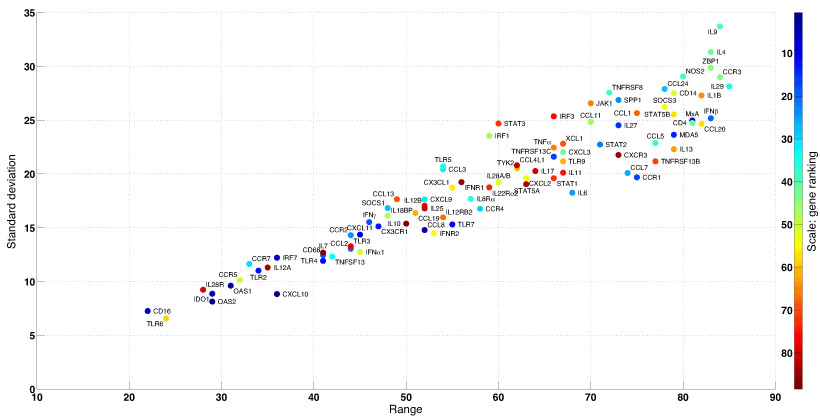

Supplement: S12 Information — (DOCX) [file pone.0126843.s018.docx]
