# Supplementary material for: Multiplexed Component Analysis to Identify Genes Contributing to the Immune Response during Acute SIV Infection
Source: PLoS One. 2015 May 18;10(5):e0126843. doi: 10.1371/journal.pone.0126843 (PMC4436129; doi:10.1371/journal.pone.0126843)

# Figures S29-S33. *Judge*-specificity of genes: relative importance of each gene using each normalization method

In each hexagonal plot, there are three main vertices representing MC-, UV-, and CV-based *judges*, shown in green, blue and red. Genes that are close to one of these vertices are more important to the corresponding class of the *judges*. Three remaining (auxiliary) vertices denote CV&UV, CV&MC, and UV&MC. Genes at the center have approximately similar importance to each class of the judges. The directions in which the relative importance of a gene decreases are from the main vertices towards the auxiliary vertices. Grid lines are drawn perpendicular to these directions and are used to calculate the coordinates for a given point. The inner color of each dot represents the average of the three ranks given by each class of the judges (obtained from Figs. 5A, 5C, and S15A-C), whereas the outer color represents the minimum of the three ranks. The congested regions in the center of the left hexagonal plots are shown on the right side.

**Figure S29. *Judge*-specificity of genes: relative importance of each gene using each normalization method, for time since infection in the spleen dataset**

**
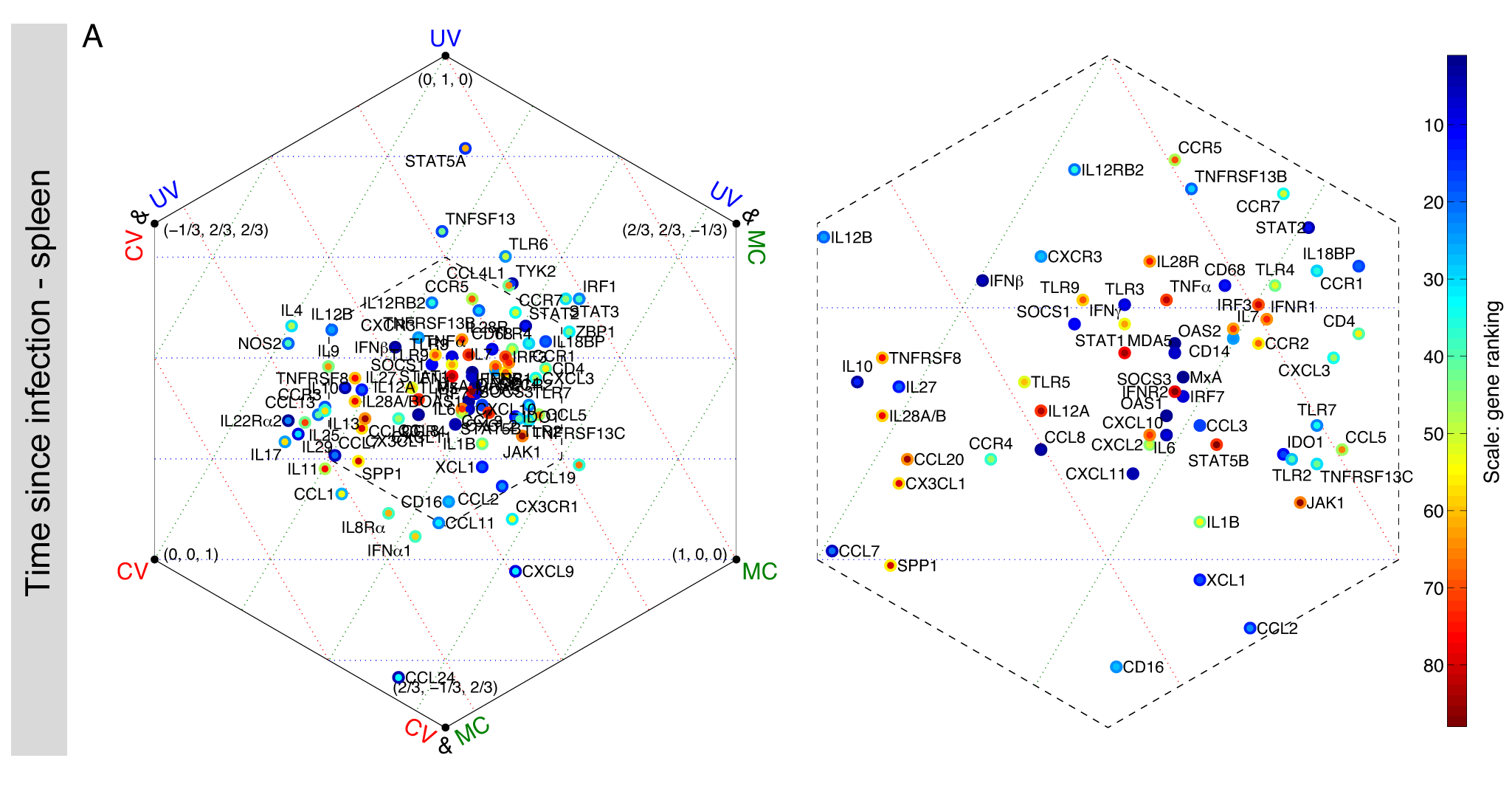
Figure S30. *Judge*-specificity of genes: relative importance of each gene using each normalization method, for time since infection in the PBMC dataset**

**
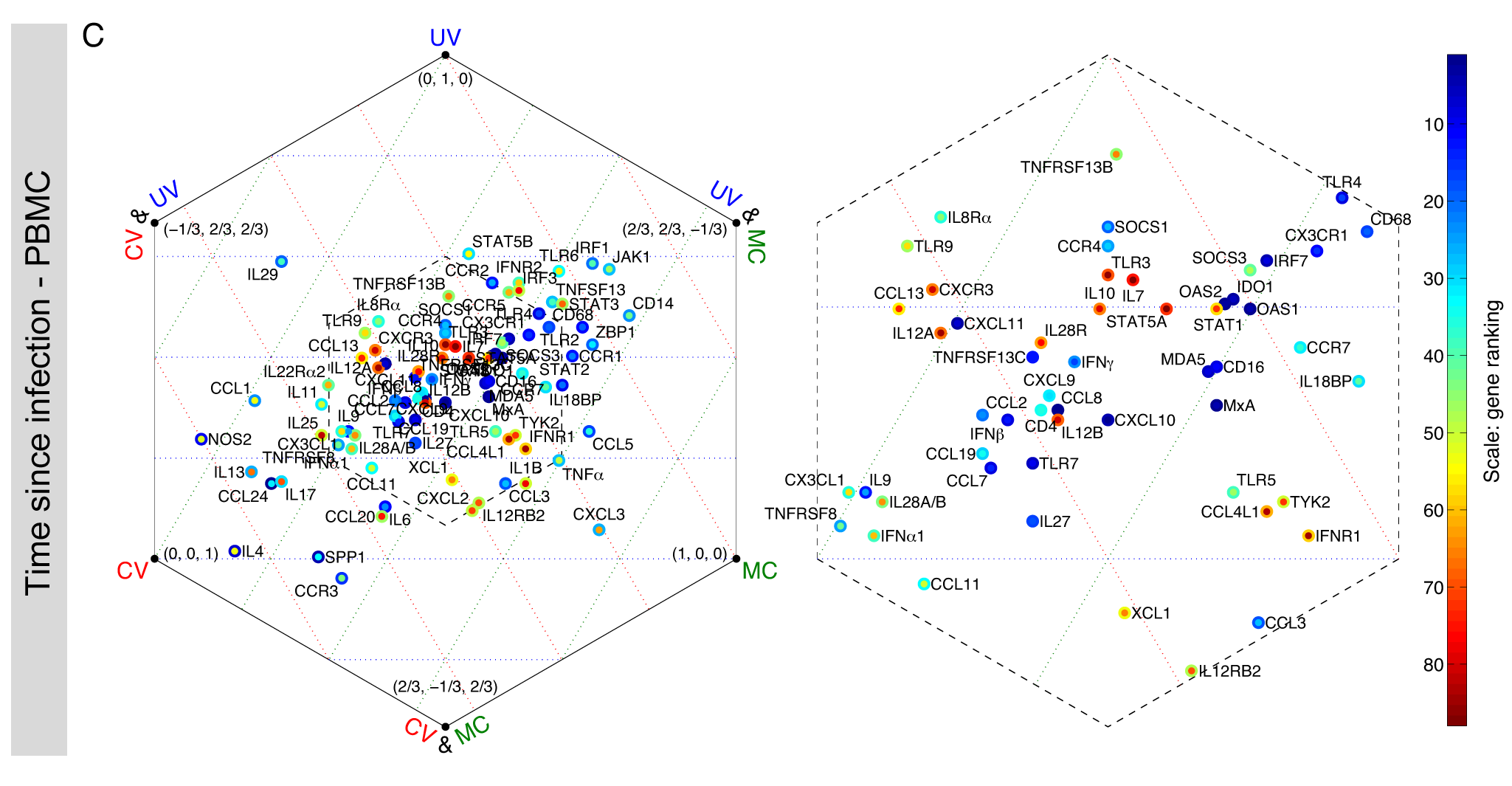
Figure S31. *Judge*-specificity of genes: relative importance of each gene using each normalization method, for SIV RNA in plasma in the spleen dataset**

**
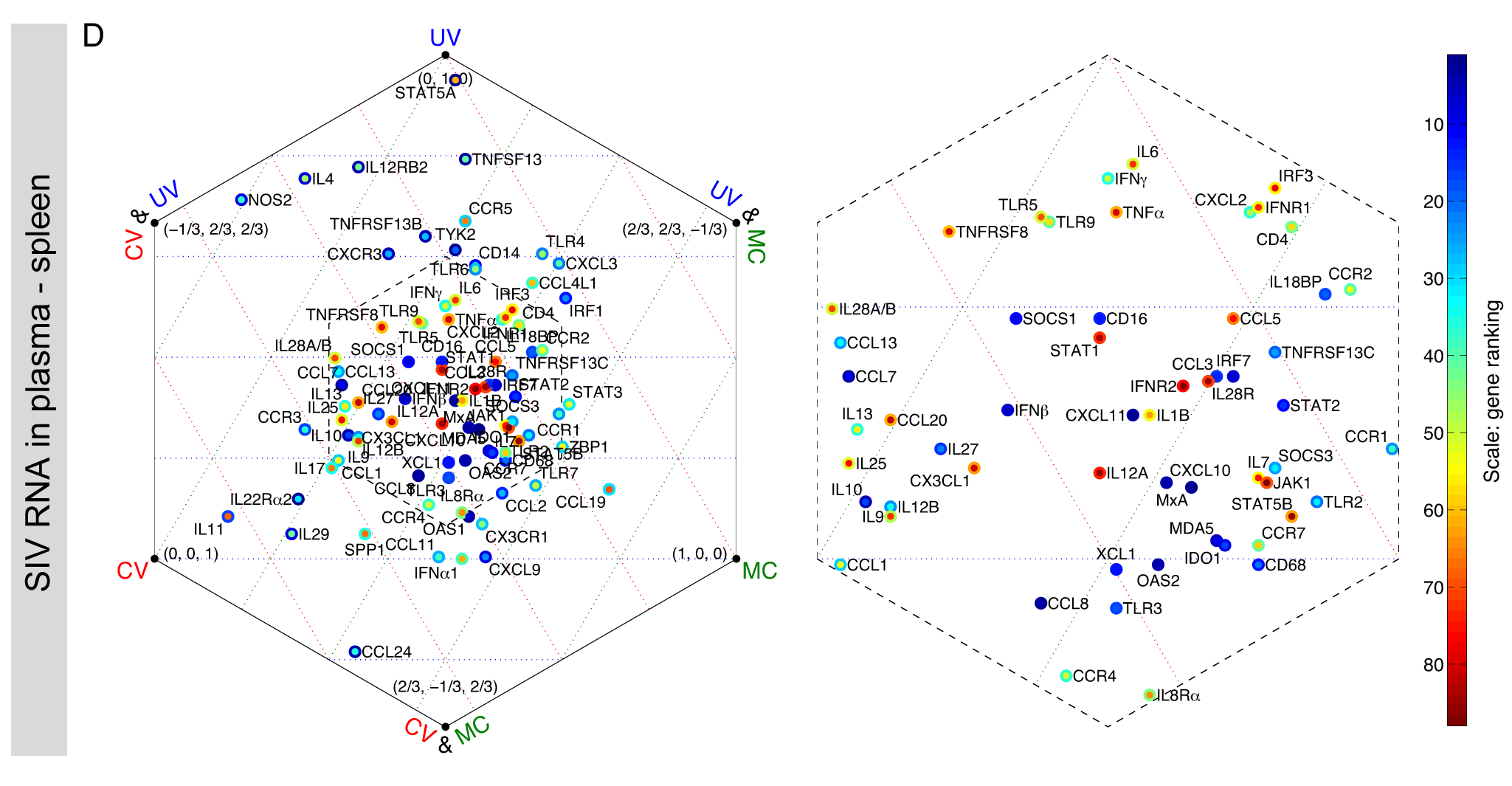
Figure S32. *Judge*-specificity of genes: relative importance of each gene using each normalization method, for SIV RNA in plasma in the MLN dataset**

**
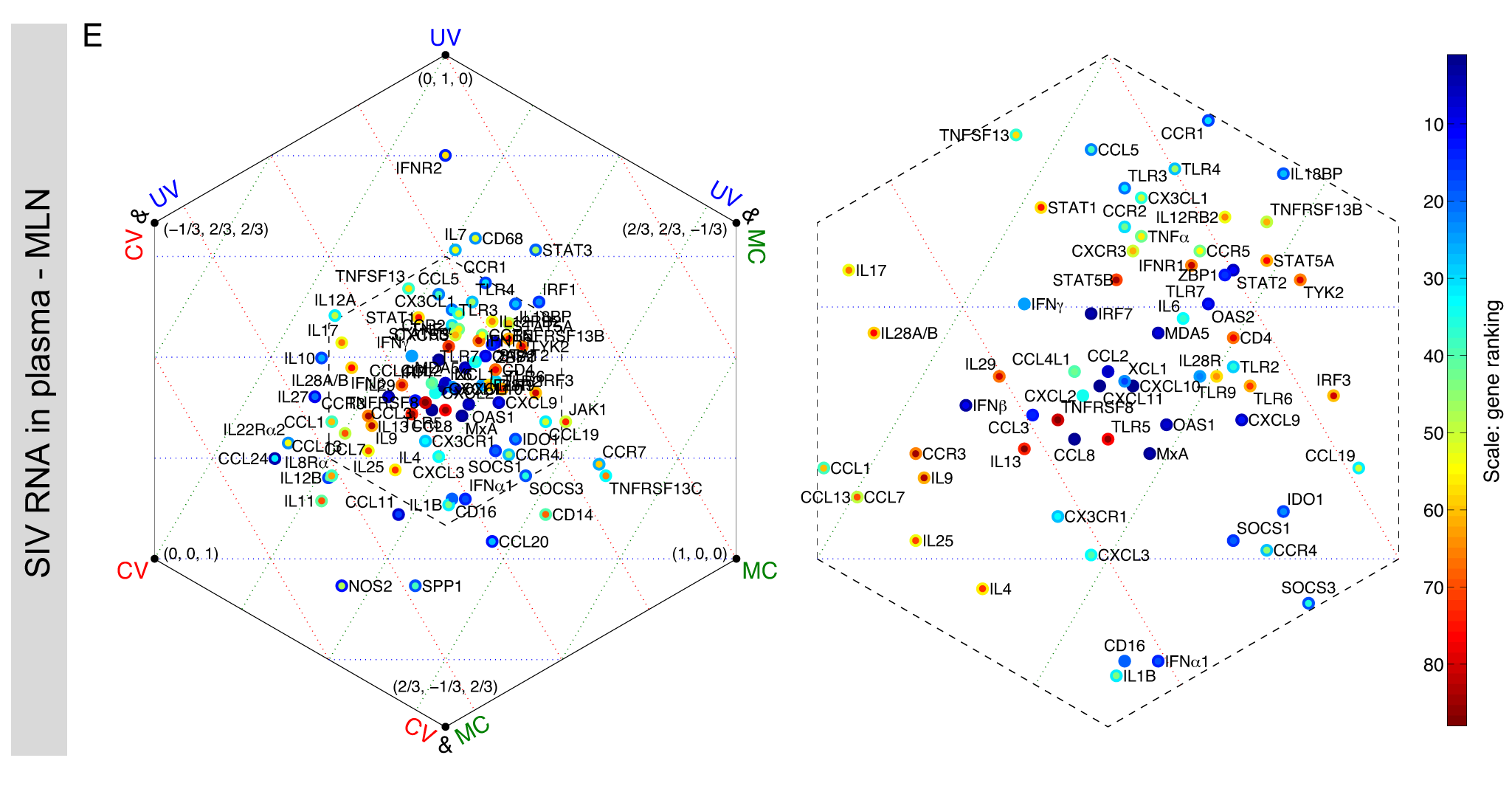
Figure S33. *Judge*-specificity of genes: relative importance of each gene using each normalization method, for SIV RNA in plasma in the PBMC dataset**

#
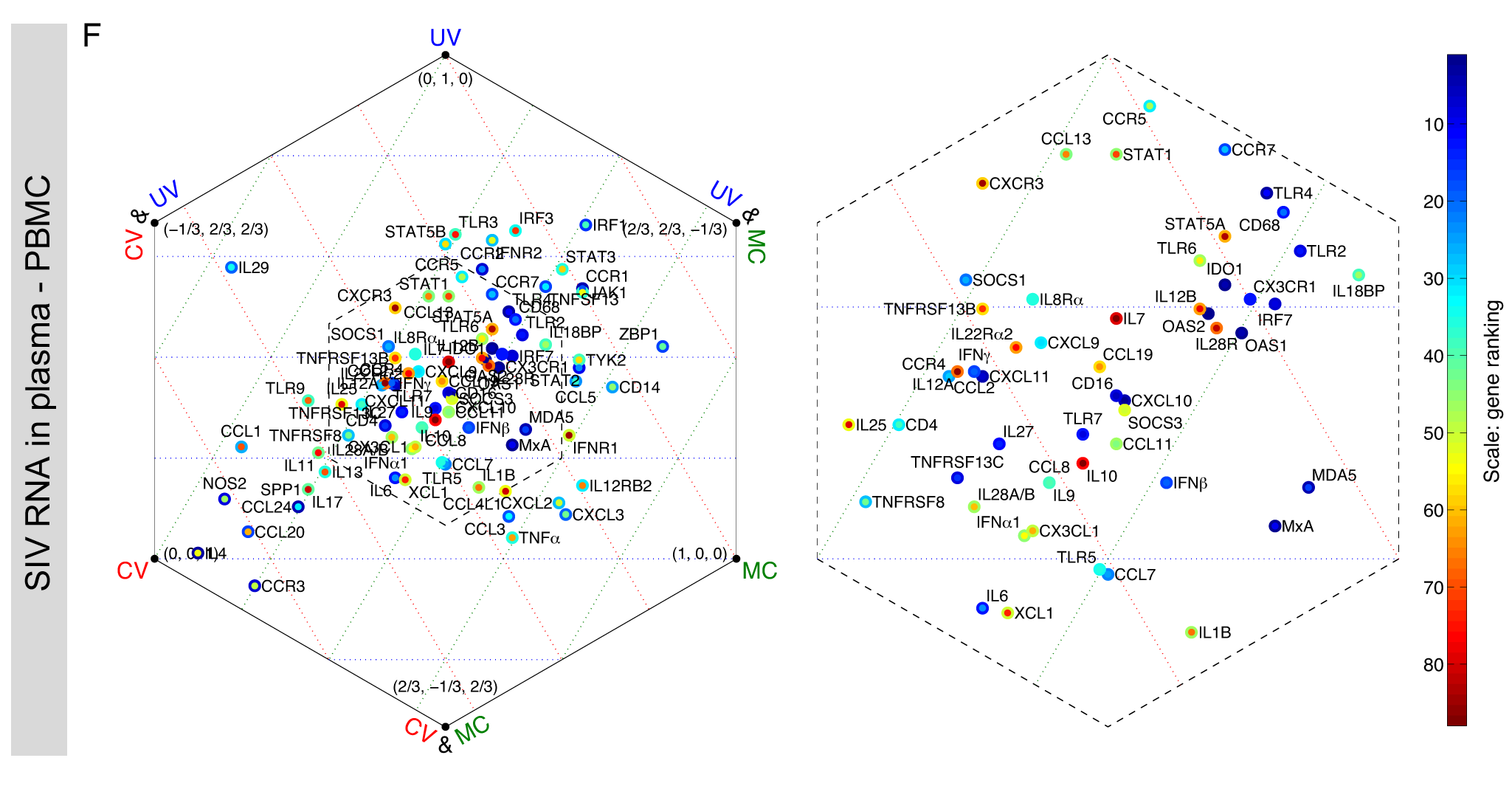

Supplement: S13 Information — (DOCX) [file pone.0126843.s019.docx]
