# Supplementary material for: Multiplexed Component Analysis to Identify Genes Contributing to the Immune Response during Acute SIV Infection
Source: PLoS One. 2015 May 18;10(5):e0126843. doi: 10.1371/journal.pone.0126843 (PMC4436129; doi:10.1371/journal.pone.0126843)
Supplement: S14 Information — (DOCX) [file pone.0126843.s020.docx]

# Figures S34-S39. *p*-value heatmap of the paired t-tests of gene rankings in all datasets and for both classification schemes (high-resolution images of the panels of Fig. 7)

In our analysis, we perform paired t-tests of gene rankings in the spleen, MLN, and PBMC datasets. The results of the tests, *p*-values, range from 1 (black) to 0 (the light copper color). Lower *p*-values suggest a more statistically significant difference between the contribution of genes. On the bottom and left axes, genes are listed from the highest average rank (the left bottom corner) to the lowest as seen in Figs. 5 and S15. The clusters, colored alternately dark and light blue along the vertical axis, determine the genes that are significantly different from genes in other clusters; the labels display the *p*-value of the paired t-tests between the cluster below the label and the cluster right to it.

**Figure S34. *p*-value heatmap of the paired t-tests of gene rankings for classification based on time since infection in the spleen dataset (Fig. 7A)**

**
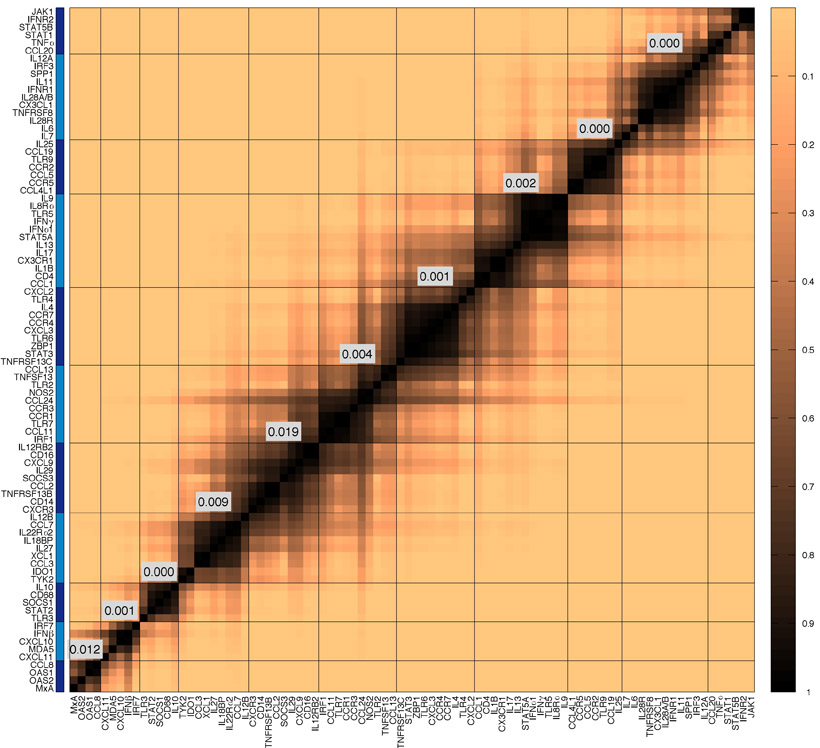
Figure S35. *p*-value heatmap of the paired t-tests of gene rankings for classification based on time since infection in the MLN dataset (Fig. 7B)**

**
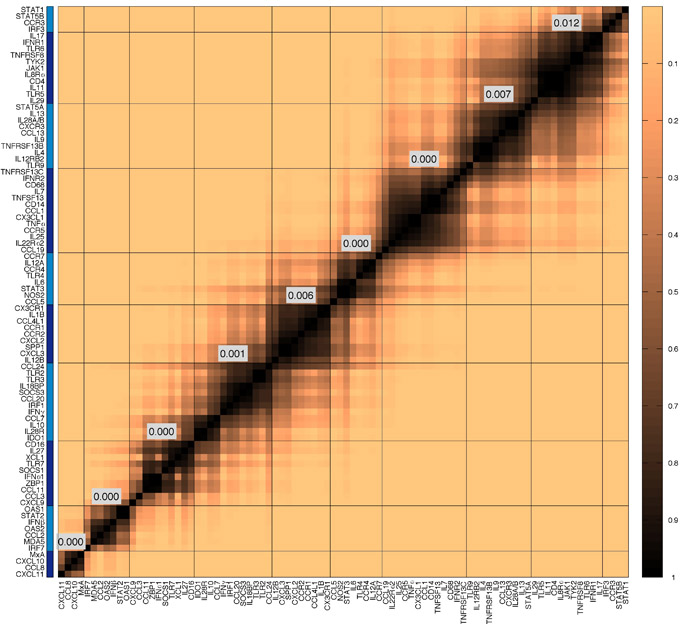
Figure S36. *p*-value heatmap of the paired t-tests of gene rankings for classification based on time since infection in the PBMC dataset (Fig. 7C)**

**
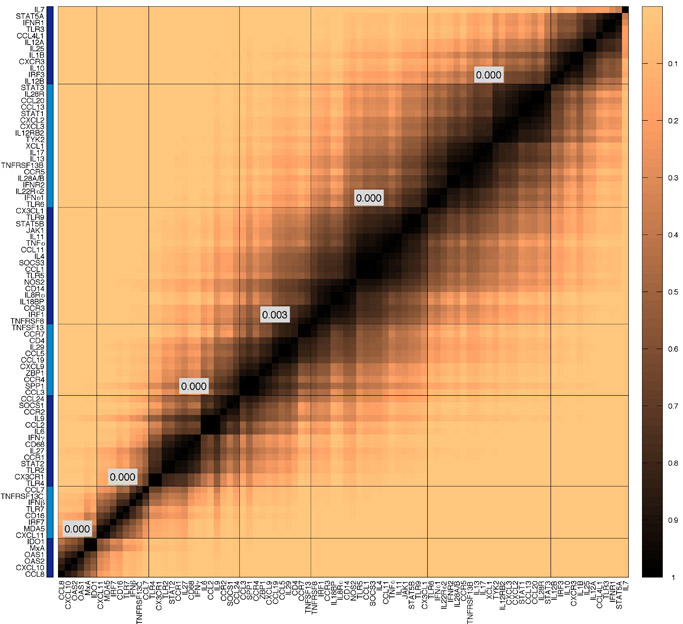
Figure S37. *p*-value heatmap of the paired t-tests of gene rankings for classification based on SIV RNA in plasma in the spleen dataset (Fig. 7D)**

**
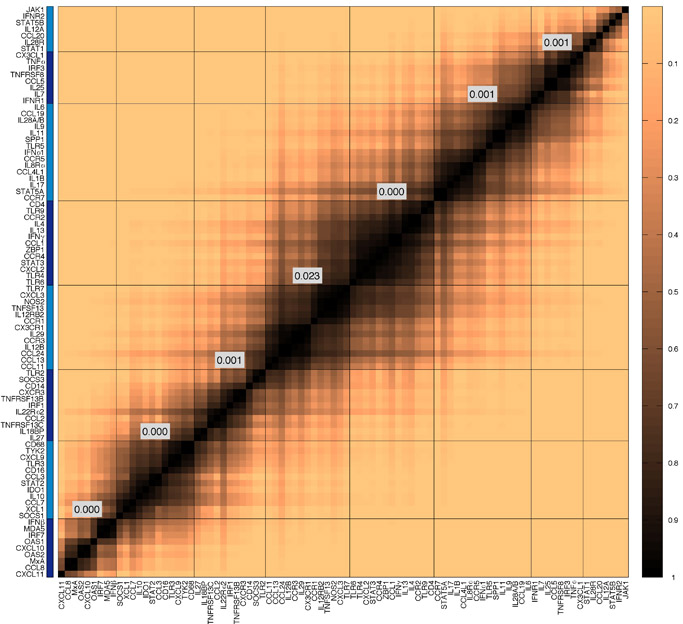
Figure S38. *p*-value heatmap of the paired t-tests of gene rankings for classification based on SIV RNA in plasma in the MLN dataset (Fig. 7E)**

**
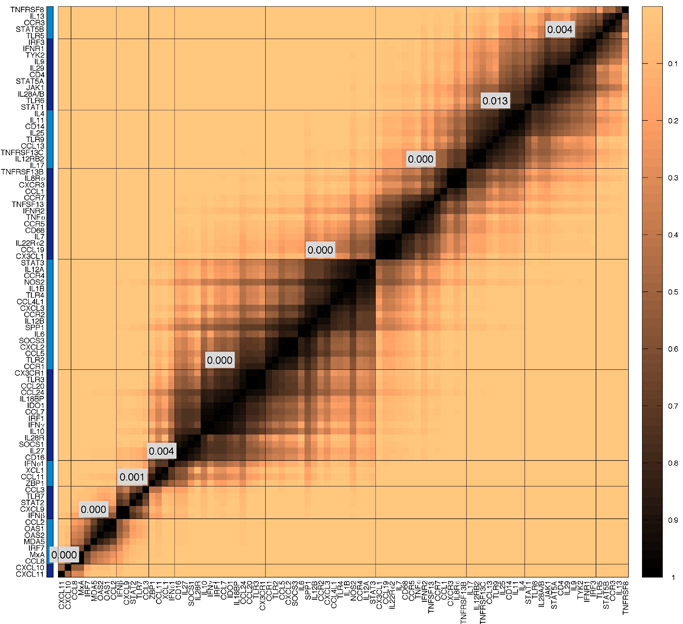
Figure S39. *p*-value heatmap of the paired t-tests of gene rankings for classification based on SIV RNA in plasma in the PBMC dataset (Fig. 7F)**

**
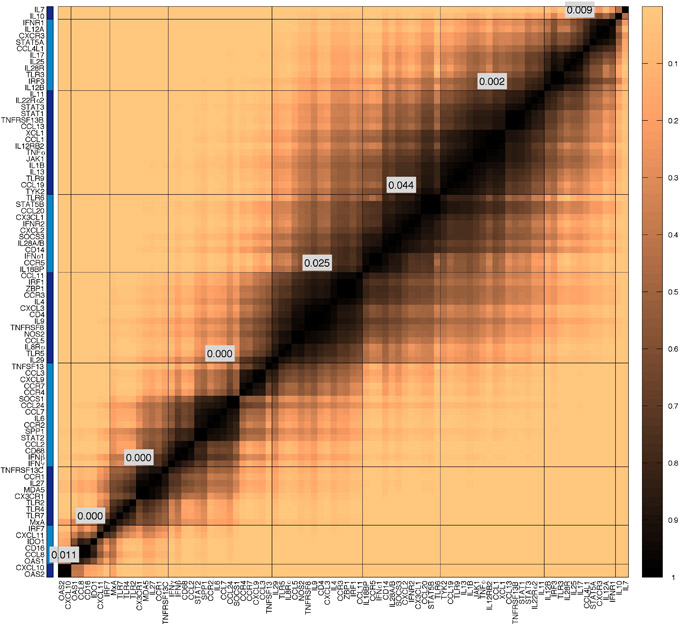
**
