# Supplementary material for: Multiplexed Component Analysis to Identify Genes Contributing to the Immune Response during Acute SIV Infection
Source: PLoS One. 2015 May 18;10(5):e0126843. doi: 10.1371/journal.pone.0126843 (PMC4436129; doi:10.1371/journal.pone.0126843)

# Figures S40-S45. Average correlation coefficient matrices in all datasets, for both classification schemes (high-resolution images of the panels of Fig. 8)

For each of the loading plots obtained from the 12 *judges*, we construct a matrix of correlation coefficients. Then, we calculate the average correlation coefficient matrix from the 12 matrices for a given dataset and a classification scheme. Dark blue and red colors represent positive and negative correlations, respectively, whereas light colors represent no correlation. For each pair of genes, we calculated the standard deviation of the 12 correlation coefficients, resulting in 88 values for each gene. The mean of these values, indicative of the level of agreement between *judges*, is shown in a bar chart on the right hand side of each panel. Smaller values suggest higher degrees of agreement between *judges* on the correlation of a gene with other genes. Genes that have approximately similar correlation patterns in the dataset are grouped into 20 gene clusters (shown in different colors along the vertical axis).

**Figure S40. Average correlation coefficient matrix for time since infection in the spleen dataset (Fig. 8A)**

**
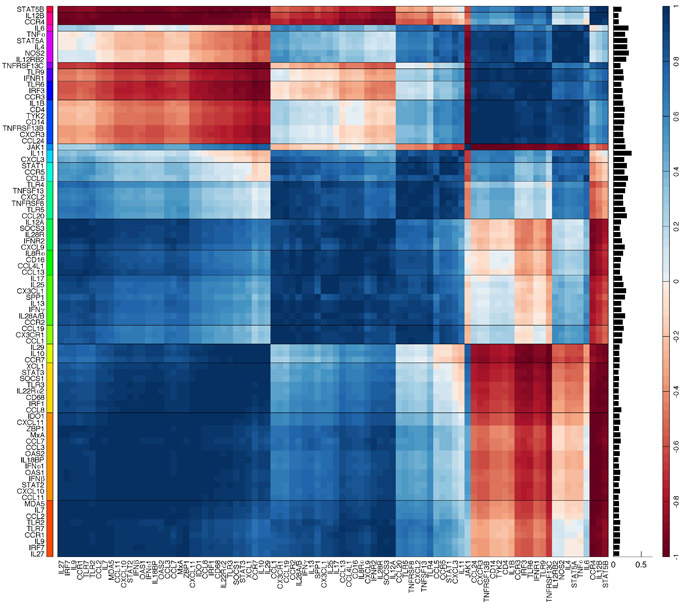
Figure S41. Average correlation coefficient matrix for time since infection in the MLN dataset (Fig. 8B)**

**
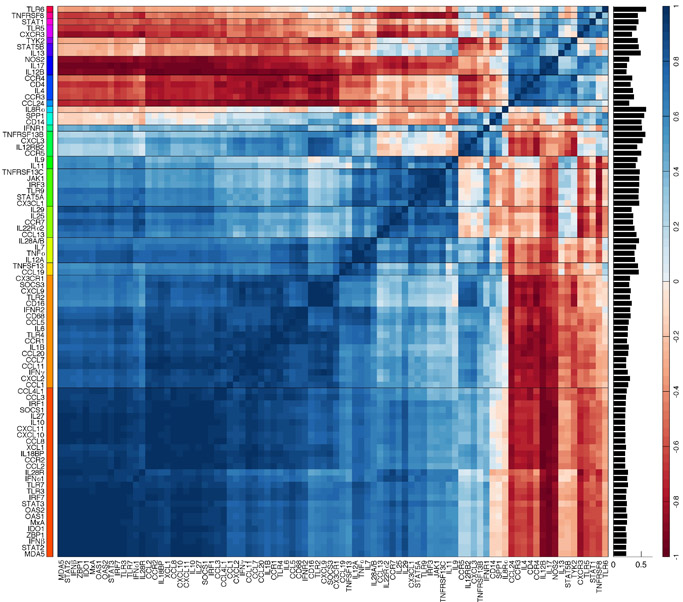
Figure S42. Average correlation coefficient matrix for time since infection in the PBMC dataset (Fig. 8C)**

**
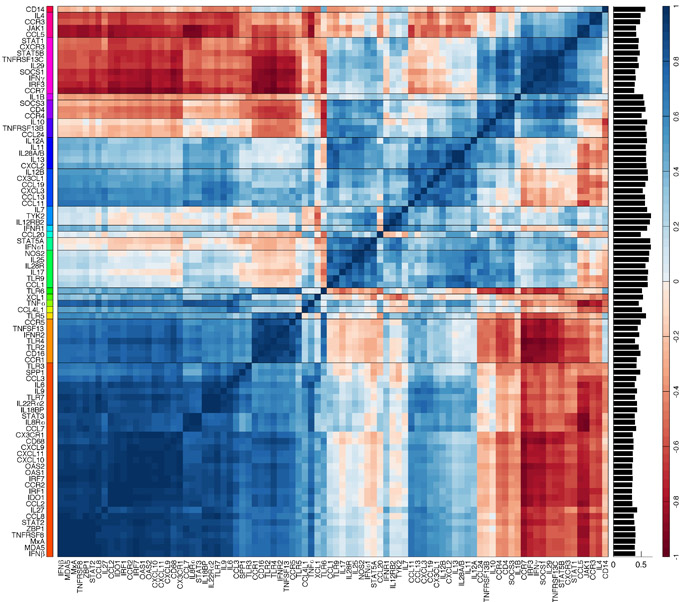
Figure S43. Average correlation coefficient matrix for SIV RNA in plasma in the spleen dataset (Fig. 8D)**

**
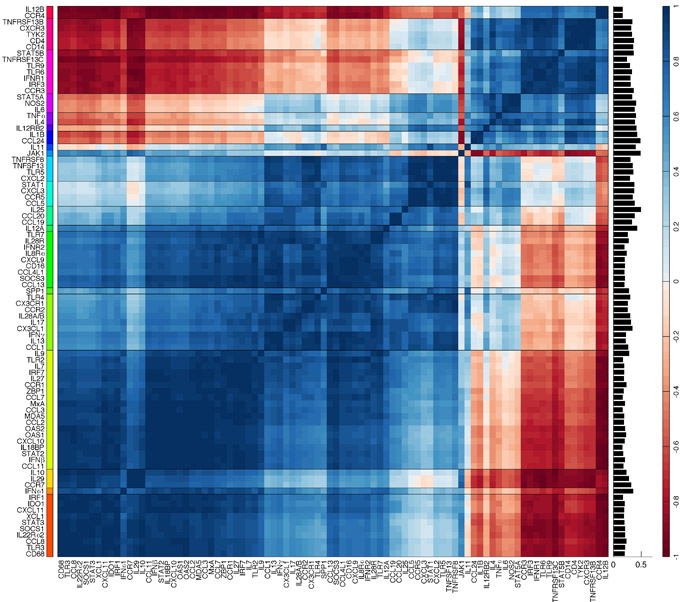
Figure S44. Average correlation coefficient matrix for SIV RNA in plasma in the MLN dataset (Fig. 8E)**

**
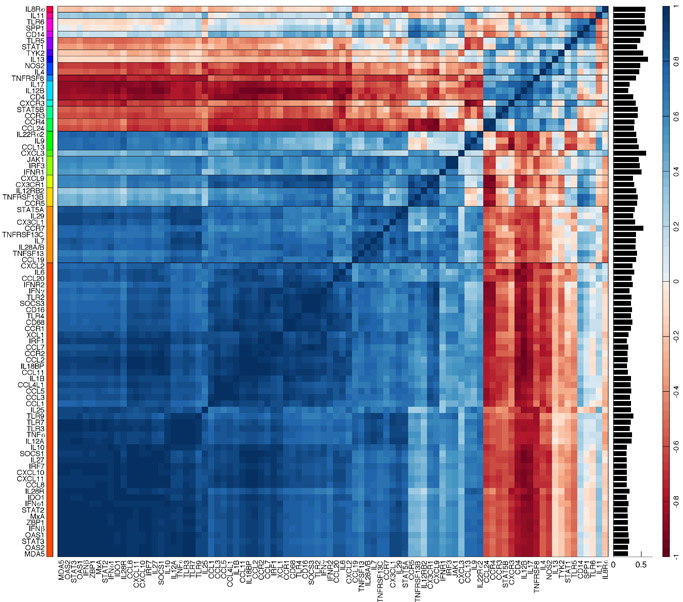
Figure S45. Average correlation coefficient matrix for SIV RNA in plasma in the PBMC dataset (Fig. 8F)**


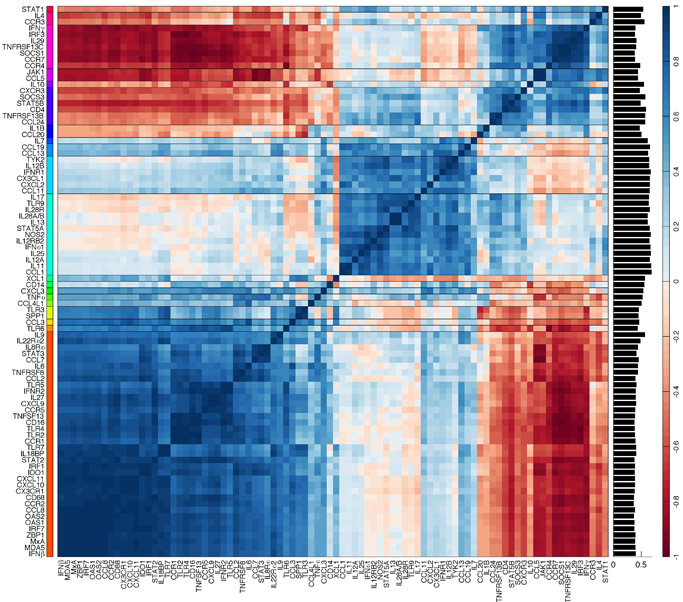

Supplement: S15 Information — (DOCX) [file pone.0126843.s021.docx]
