# Supplementary material for: Multiplexed Component Analysis to Identify Genes Contributing to the Immune Response during Acute SIV Infection
Source: PLoS One. 2015 May 18;10(5):e0126843. doi: 10.1371/journal.pone.0126843 (PMC4436129; doi:10.1371/journal.pone.0126843)
Supplement: S18 Information — (DOCX) [file pone.0126843.s024.docx]

# Figures S48-S53. Gene expression profiles in all datasets and for both classification schemes

The gene expression profiles are grouped according to the 20 clusters obtained in Fig. 8. The number shown of the right top corner of each subplot represents the cluster number. Each gene cluster consists of genes that have approximately similar correlation patterns in the dataset. The curve colors match the cluster colors in Figs. 8A-F, respectively.

**Figure S48. Gene expression profiles in the spleen dataset for classification based on time since infection**

**
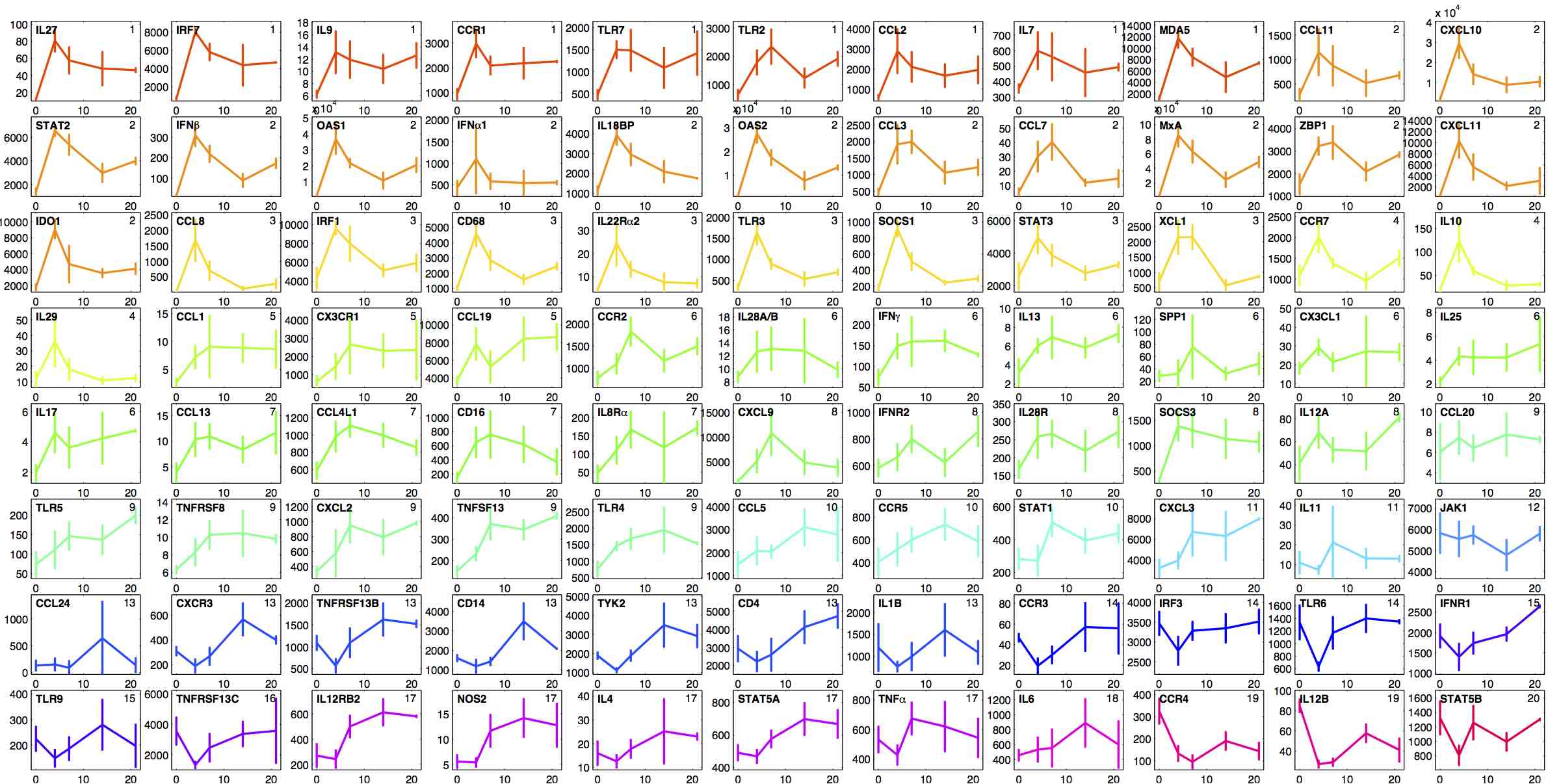
**

**Figure S49. Gene expression profiles in the MLN dataset for classification based on time since infection**

**
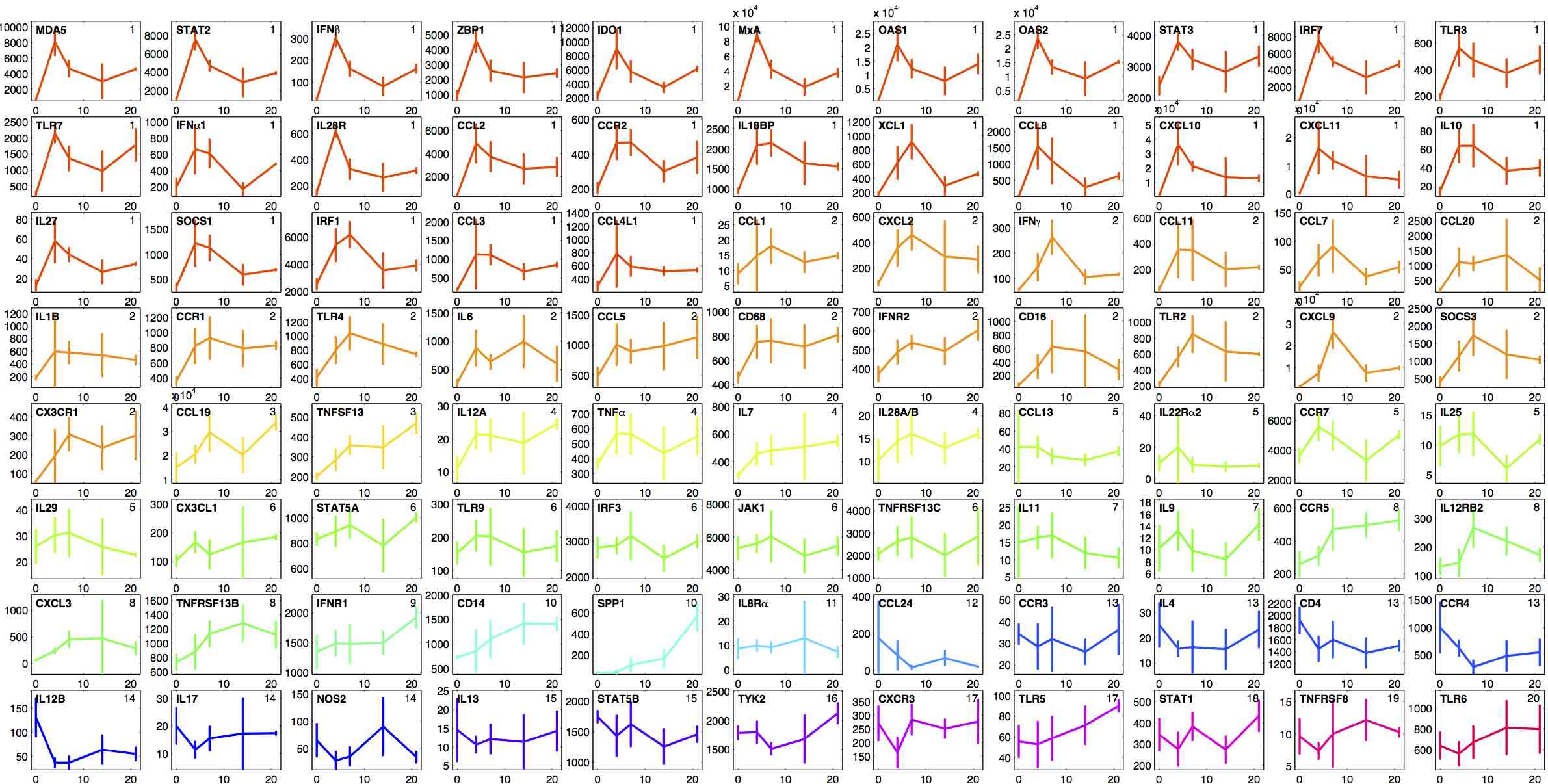
Figure S50. Gene expression profiles in the PBMC dataset for classification based on time since infection**

**
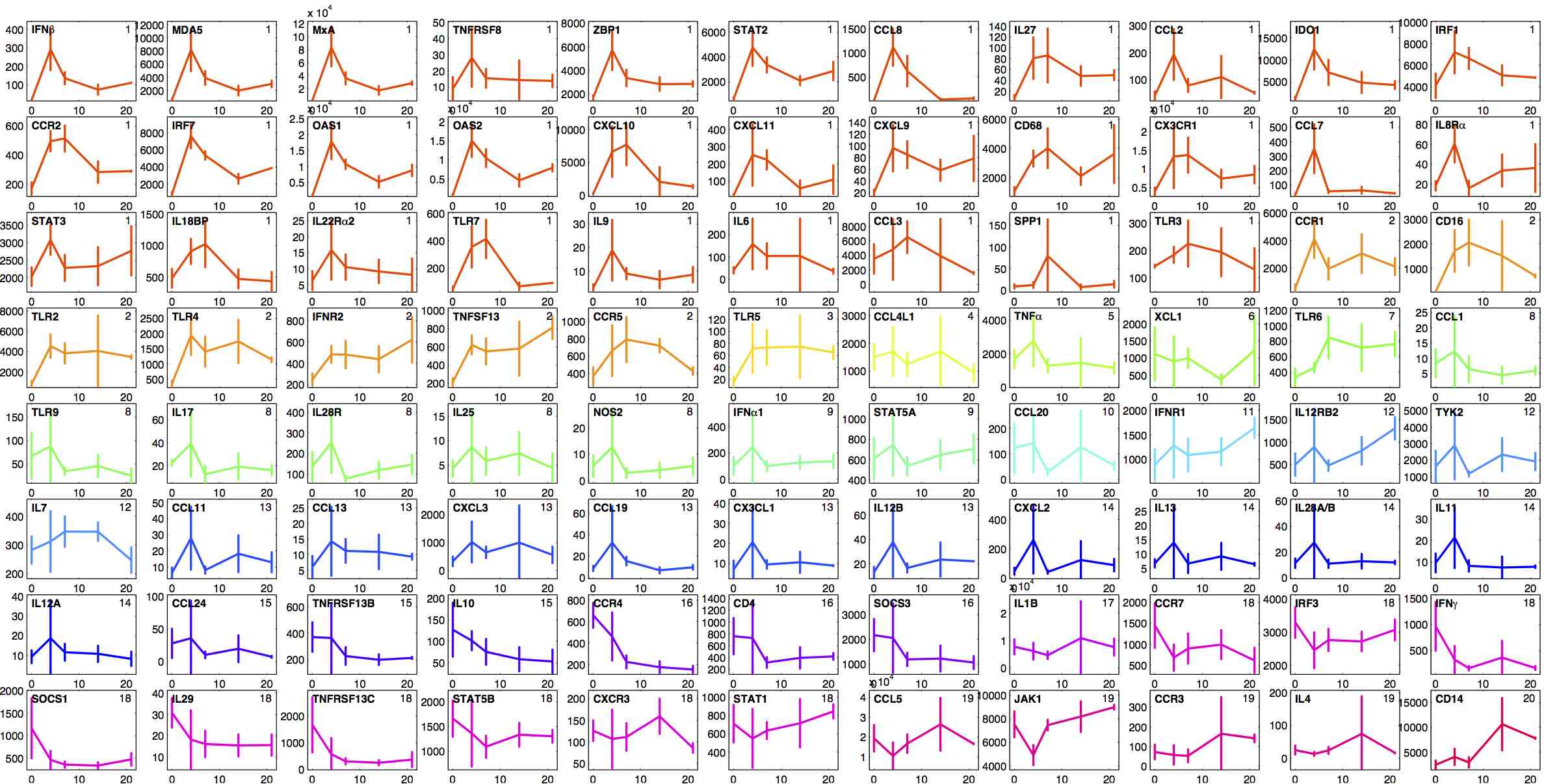
Figure S51. Gene expression profiles in the spleen dataset for classification based SIV RNA in plasma**

**
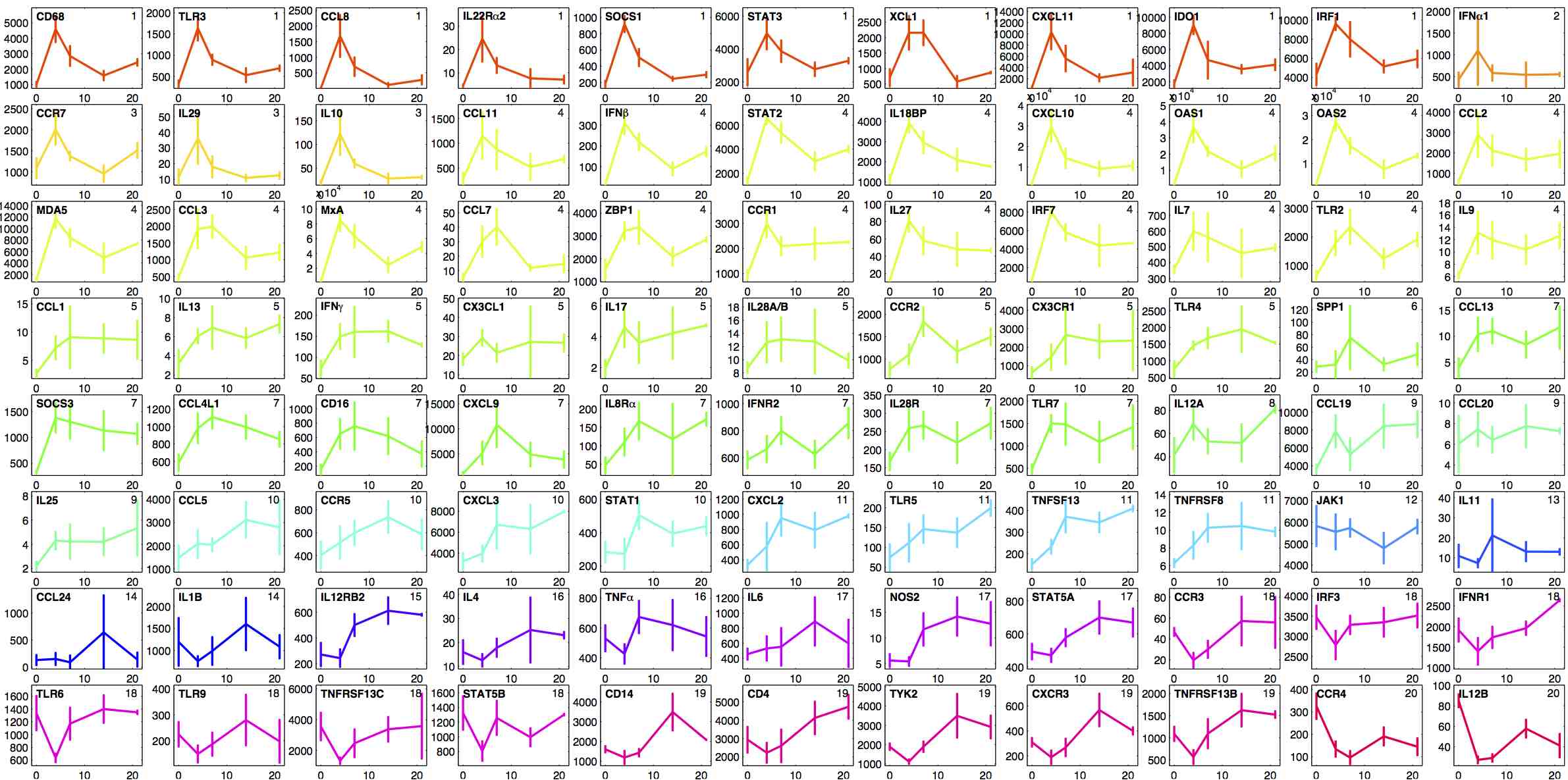
Figure S52. Gene expression profiles in the MLN dataset for classification based SIV RNA in plasma**

**
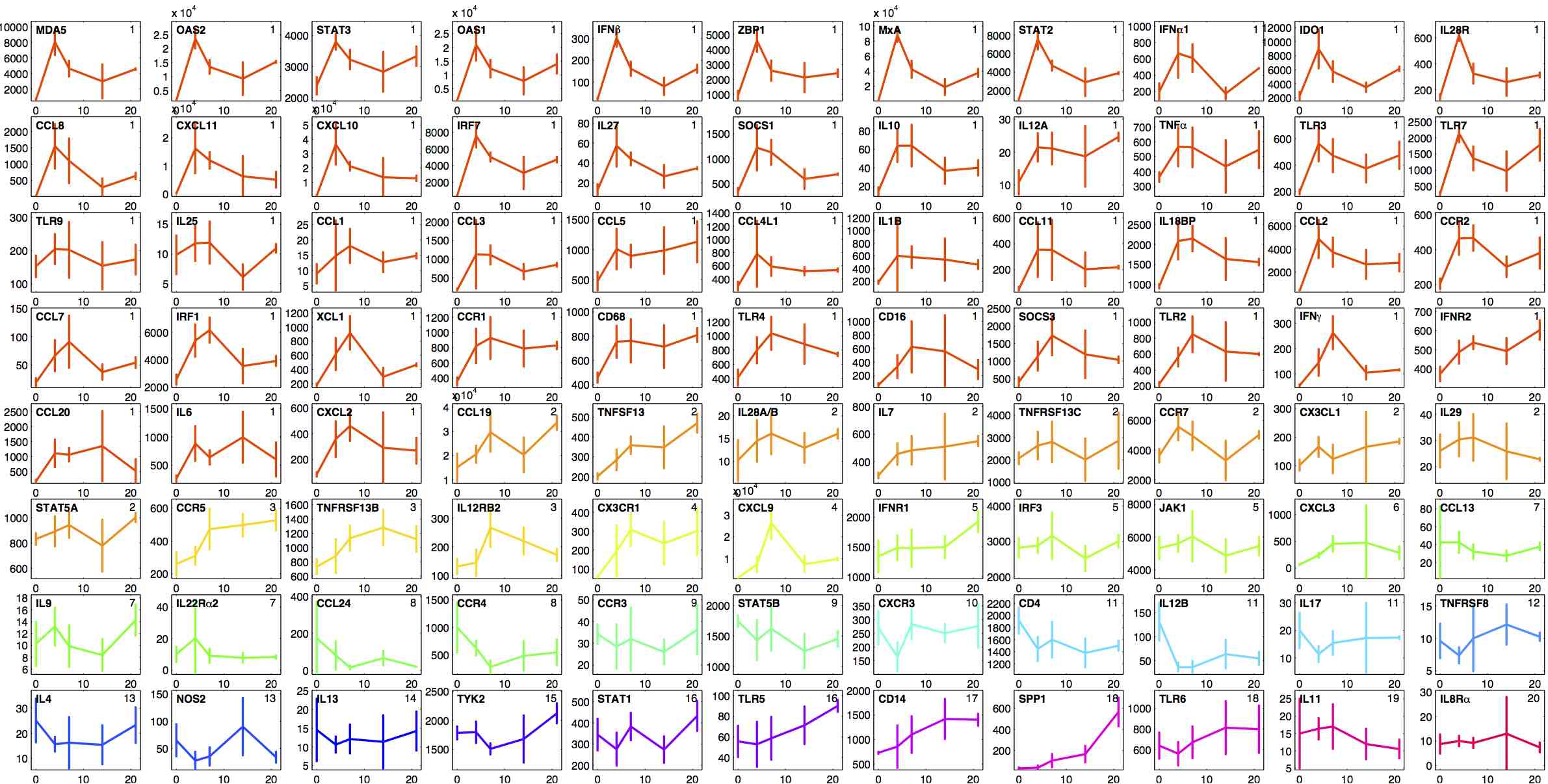
Figure S53. Gene expression profiles in the PBMC dataset for classification based SIV RNA in plasma**

**
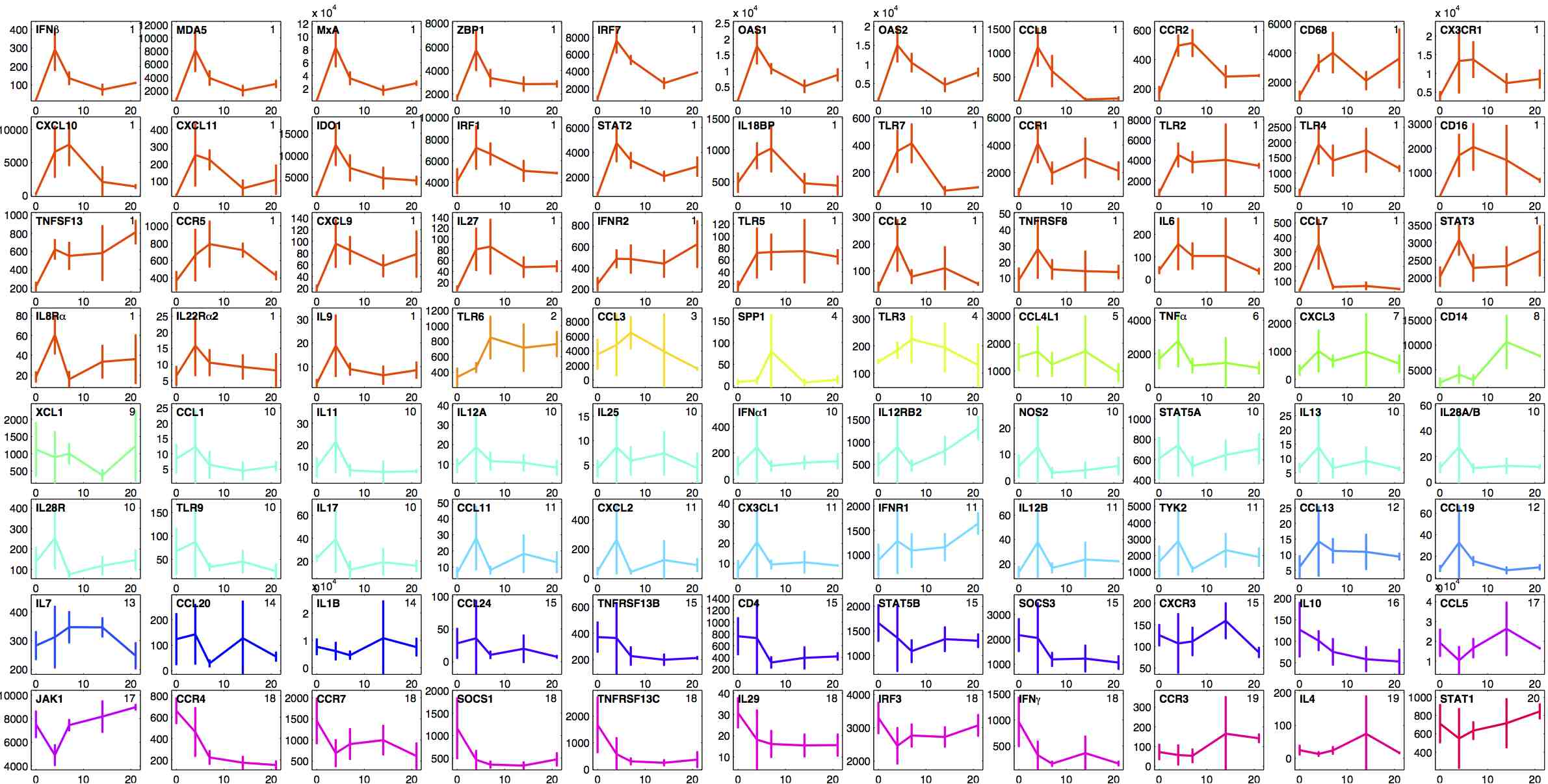
**
