# Supplementary material for: Multiplexed Component Analysis to Identify Genes Contributing to the Immune Response during Acute SIV Infection
Source: PLoS One. 2015 May 18;10(5):e0126843. doi: 10.1371/journal.pone.0126843 (PMC4436129; doi:10.1371/journal.pone.0126843)
Supplement: S1 Table — (DOCX) [file pone.0126843.s026.docx]

# Table S1. List of genes measured by Nanostring technology

| **Definition** | **GENE** | **Full name or Alias** |
| --- | --- | --- |
| **Housekeeping Genes** | Actin | Actin |
|  | GAPDH | Glyceraldehyde 3-phosphate dehydrogenase |
|  | HPRT | Hypoxanthine-guanine phosphoribosyltransferase |
|  | PBGD | Porphobilinogen deaminase |
| **Chemokines** | CCL1 | CCL1 |
|  | CCL2 | Monocyte chemotactic protein (MCP) 1 |
|  | CCL3 | Macrophage inflammatory protein (MIP) 1α |
|  | CCL4L1 | MIP-1β (similar to human CCL4) |
|  | CCL5 | RANTES |
|  | CCL7 | MCP-3 |
|  | CCL8 | MCP-2 |
|  | CCL11 | Eotaxin-1 |
|  | CCL13 | MCP-4 |
|  | CCL19 | MIP-3β |
|  | CCL20 | MIP-3α |
|  | CCL24 | Eotaxin-2 |
|  | CX3CL1 | Fractalkine |
|  | CXCL2 | MIP-2α |
|  | CXCL3 | MIP-2β |
|  | CXCL9 | Monokine induced by gamma interferon (MIG) |
|  | CXCL10 | Interferon gamma-induced protein 10 (IP10) |
|  | CXCL11 | IP-9 |
|  | XCL1 | Lymphotactin |
| **Chemokine receptors** | CCR1 | CCR1 |
|  | CCR2 | CCR2 |
|  | CCR3 | CCR3 |
|  | CCR4 | CCR4 |
|  | CCR5 | CCR5 |
|  | CCR7 | CCR7 |
|  | CXCR3 | CXCR3 |
|  | CX3CR1 | CX3CR1 |
| **Cluster of differentiation (CD)** | CD4 | CD4 |
|  | CD14 | CD14 |
|  | CD16 | CD16 |
|  | CD68 | CD68 |
| **Interferons** | IFNα1 | IFNα1 |
|  | IFNβ | IFNβ |
|  | IFNγ | IFNγ |
| **Type I Interferon receptors** | IFNR1 | IFNR1 |
|  | IFNR2 | IFNR2 |
| **Interleukines** | IL1B | IL1b |
|  | IL4 | IL4 |
|  | IL6 | IL6 |
|  | IL7 | IL7 |
|  | IL9 | IL9 |
|  | IL18BP | IL18bp |
|  | IL10 | IL10 |
|  | IL11 | IL11 |
|  | IL12A | IL12A |
|  | IL12B | IL12b |
|  | IL13 | IL13 |
|  | IL17 | IL17 |
|  | IL25 | IL25 |
|  | IL27 | IL27 |
|  | IL28A/B | IL28A/B |
|  | IL29 | IL29 |
| **Cytokine receptors** | IL8Rα | CXCR1 or CD128 |
|  | IL12RB2 | IL12Rb2 |
|  | IL22Rα2 | IL22Rα2 |
|  | IL28R | IL28R |
| **Interferon regulatory factors** | IRF1 | IRF1 |
|  | IRF3 | IRF3 |
|  | IRF7 | IRF7 |
| **Signal transducers and activators of transcription** | STAT1 | STAT1 |
|  | STAT2 | STAT2 |
|  | STAT3 | STAT3 |
|  | STAT5A | STAT5a |
|  | STAT5B | STAT5b |
| **Kinases** | JAK1 | Janus kinase 1 |
|  | TYK2 | Tyrosine kinase 2 |
| **Other enzymes** | IDO1 | indoleamine 2,3-dioxygenase 1 |
|  | NOS2 | Nitric oxide synthase 2 |
| **Interferon stimulated genes** | MxA | Myxovirus resistance 1 |
|  | OAS1 | 2'-5'-oligoadenylate synthetases |
|  | OAS2 |  |
| **Suppressor of cytokine signaling** | SOCS1 | SOCS1 |
|  | SOCS3 | SOCS3 |
| **Secreted phosphoprotein 1** | SPP1 | Osteopontin |
| **Pathogen pattern recognition receptors** | TLR2 | Toll like receptors |
|  | TLR3 |  |
|  | TLR4 |  |
|  | TLR5 |  |
|  | TLR6 |  |
|  | TLR7 |  |
|  | TLR9 |  |
|  | MDA5 | Melanoma Differentiation-Associated protein 5 |
|  | ZBP1 | Z-DNA-binding protein 1 |
| **Tumor necrosis factor-α** | TNFα | Cachexin |
| **Tumor necrosis factor ligand** | TNFSF13 | CD256 |
| **Tumor necrosis factor receptor** | TNFRSF13B | CD267 |
|  | TNFRSF13C | CD268 |
|  | TNFRSF8 | CD30 |
