# Supplementary material for: Multiplexed Component Analysis to Identify Genes Contributing to the Immune Response during Acute SIV Infection
Source: PLoS One. 2015 May 18;10(5):e0126843. doi: 10.1371/journal.pone.0126843 (PMC4436129; doi:10.1371/journal.pone.0126843)
Supplement: S2 Table — (DOCX) [file pone.0126843.s027.docx]

# Table S2. List of top two classifier PCs for each of the *judges*

|  | **Time since infection** | | | **SIV RNA in plasma** | | |
| --- | --- | --- | --- | --- | --- | --- |
|  | **Spleen** | **MLN** | **PBMC** | **Spleen** | **MLN** | **PBMC** |
| ***J1: (Orig, MC, PCA)*** | PC1-PC5 | PC1-PC2 | PC1-PC5 | PC1-PC3 | PC1-PC2 | PC1-PC5 |
| ***J2: (Orig, UV, PCA)*** | PC1-PC2 | PC1-PC3 | PC2-PC5 | PC1-PC2 | PC1-PC4 | PC2-PC5 |
| ***J3: (Orig, CV, PCA)*** | PC1-PC2 | PC1-PC6 | PC1-PC2 | PC1-PC3 | PC1-PC3 | PC2-PC3 |
| ***J4: (Log2, MC, PCA)*** | PC1-PC2 | PC1-PC2 | PC1-PC5 | PC1-PC4 | PC1-PC3 | PC1-PC5 |
| ***J5: (Log2, UV, PCA)*** | PC1-PC2 | PC1-PC6 | PC1-PC4 | PC1-PC2 | PC1-PC8 | PC1-PC4 |
| ***J6: (Log2, CV, PCA)*** | PC1-PC2 | PC1-PC6 | PC2-PC5 | PC1-PC2 | PC1-PC3 | PC2-PC5 |
| ***J7: (Orig, MC, PLS)*** | PC1-PC4 | PC1-PC3 | PC1-PC5 | PC1-PC4 | PC1-PC4 | PC1-PC4 |
| ***J8: (Orig, UV, PLS)*** | PC1-PC2 | PC2-PC3 | PC1-PC4 | PC1-PC3 | PC3-PC5 | PC3-PC4 |
| ***J9: (Orig, CV, PLS)*** | PC1-PC2 | PC1-PC2 | PC3-PC4 | PC1-PC3 | PC2-PC7 | PC3-PC8 |
| ***J10: (Log2, MC, PLS)*** | PC1-PC2 | PC1-PC2 | PC1-PC2 | PC2-PC4 | PC2-PC4 | PC2-PC5 |
| ***J11: (Log2, UV, PLS)*** | PC1-PC2 | PC1-PC2 | PC1-PC2 | PC1-PC2 | PC1-PC5 | PC2-PC8 |
| ***J12: (Log2, CV, PLS)*** | PC1-PC2 | PC1-PC2 | PC3-PC7 | PC2-PC4 | PC1-PC2 | PC4-PC7 |
