# Supplementary material for: Multiplexed Component Analysis to Identify Genes Contributing to the Immune Response during Acute SIV Infection
Source: PLoS One. 2015 May 18;10(5):e0126843. doi: 10.1371/journal.pone.0126843 (PMC4436129; doi:10.1371/journal.pone.0126843)
Supplement: S3 Table — (DOCX) [file pone.0126843.s028.docx]

# Table S3. Friedman test results for gene rankings

| **Time of Infection** | | | **Plasma SIV RNA** | | |
| --- | --- | --- | --- | --- | --- |
| **Spleen** | **MLN** | **PBMC** | **Spleen** | **MLN** | **PBMC** |
| 7.99e-83 | 4.53e-90 | 2.00e-43 | 4.78e-48 | 1.20e-78 | 1.35e-38 |
