# Supplementary material for: Multiplexed Component Analysis to Identify Genes Contributing to the Immune Response during Acute SIV Infection
Source: PLoS One. 2015 May 18;10(5):e0126843. doi: 10.1371/journal.pone.0126843 (PMC4436129; doi:10.1371/journal.pone.0126843)
Supplement: S4 Table — (DOCX) [file pone.0126843.s029.docx]

# Table S4. Classification results using the top seven highly contributing genes

| **Time of Infection** | | | **Plasma SIV RNA** | | |
| --- | --- | --- | --- | --- | --- |
| **Spleen** | **MLN** | **PBMC** | **Spleen** | **MLN** | **PBMC** |
| 87.5% | 87.5% | 87.5% | 83.33% | 79.17% | 83.33% |
